# Optimal Statistical Incorporation of Independent Feature Stability Information into Radiomics Studies

**DOI:** 10.1038/s41598-020-57739-8

**Published:** 2020-01-20

**Authors:** Michael Götz, Klaus H. Maier-Hein

**Affiliations:** 1 Division of Medical Image Computing, German Cancer Research Center (DKFZ), Heidelberg, Germany; 2 National Center for Tumor Diseases (NCT), Heidelberg, Germany

**Keywords:** Biomarkers, Cancer

## Abstract

Conducting side experiments termed robustness experiments, to identify features that are stable with respect to rescans, annotation, or other confounding effects is an important element in radiomics research. However, the matter of how to include the finding of these experiments into the model building process still needs to be explored. Three different methods for incorporating prior knowledge into a radiomics modelling process were evaluated: the naïve approach (ignoring feature quality), the most common approach consisting of removing unstable features, and a novel approach using data augmentation for information transfer (DAFIT). Multiple experiments were conducted using both synthetic and publicly available real lung imaging patient data. Ignoring additional information from side experiments resulted in significantly overestimated model performances meaning the estimated mean area under the curve achieved with a model was increased. Removing unstable features improved the performance estimation, while slightly decreasing the model performance, i.e. decreasing the area under curve achieved with the model. The proposed approach was superior both in terms of the estimation of the model performance and the actual model performance. Our experiments show that data augmentation can prevent biases in performance estimation and has several advantages over the plain omission of the unstable feature. The actual gain that can be obtained depends on the quality and applicability of the prior information on the features in the given domain. This will be an important topic of future research.

## Introduction

Radiomics signatures have recently received much attention as they are non-invasive and cost-efficient biomarkers that reflect the affected tissue as a whole. Radiomics signatures are therefore used for a wide range of different entities and at various levels of treatment^1–5^. They provide complementary information and are also used to extend other biomarker-based models and further increase prediction quality^3,6^.

Radiomics signatures represent a lesion or subject using a high-dimensional vector consisting of different quantitative features which has been extracted from the annotated regions of interest in radiographical images. These features can be learned or hand-crafted and represent the different characteristics of the selected regions, e.g. the shape of the region, the distribution of intensities or the local image texture information. Once calculated, the feature vectors can be used under the assumption that they denote specific tissue characteristics. Bearing this in mind, they can be used find meaningful signatures which are predictive for the question at hand.

The values of a radiomics feature can depend on many different variables, not solely on the desired correlation with clinically relevant parameters or outcome^7–11^, but also on variables such as imaging parameters. Imaging parameters such as resolution, presence or absence of artefacts, the actual imaging device used, the reconstruction algorithm for computed tomography (CT) images, or the sequence for magnetic resonance imaging (MRI) are some examples of possible influences. Other effects results from the annotations of the regions of interest^12–15^: inter-rater differences, time spent for each annotation, the trade-off between sensitivity and specificity, and the application of (semi-) automatic tools. In order to obtain meaningful radiomics signatures, it is therefore important to ensure that the signatures found are insensitive to all effects except the desired outcome value.

In theory, the stability of radiomics signatures should be established by systematically excluding these possible sources of variation during a study. However, this is often infeasible due to clinical limitations and time constraints. For example, patients are usually only scanned once due to increased time expenditure, increased costs, and the ethical problems which arise from scanning them multiple times. Similar, the vendor of the imaging device is usually defined by the available devices. The common use of retrospective cohorts, for example, completely prevents subsequent adaptation of imaging parameters^16^. While repeated annotations of images are possible in principle, they are often avoided due to the increase in time and cost involved^16^. In order to continue to control the imaging- and annotation- induced effects on radiomics features, additional side-studies on the stability of the different features are commonly carried out and recommended by the Radiomics Quality Score^16,17^.

**Figure 1:**
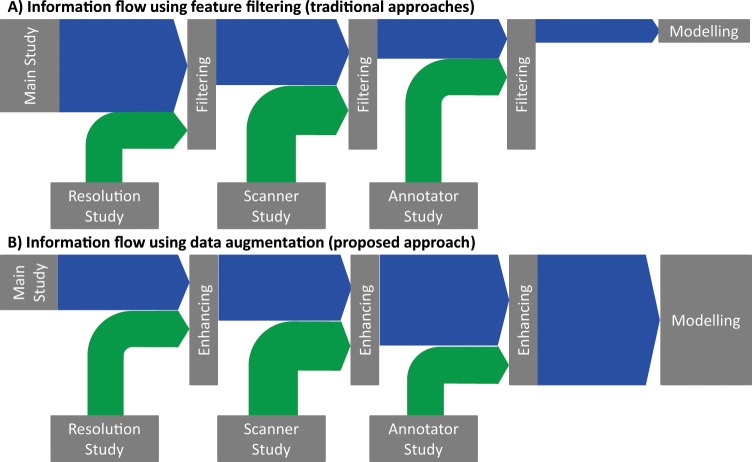
Visualization of the information flow with the traditional approach (**A**) and the proposed approach (**B**). Current approaches use the information obtained from additional experiments or studies to identify unstable features and remove those features and thus remove the information contained in the features from the main study. This effectively reduces the information available for use in the model building process. In opposition to this, we propose that the available information in the main study be augmented according to the additional information, thus enabling more advanced model building and feature selection approaches.

Typically, these side-studies follow scan-rescan or annotate-reannotate paradigms on a small patient-cohort or in a phantom study, thus reducing the cost and burden for the majority of patients included in the main studies while still providing the necessary prior information on feature stability. Currently, there is no one best way to incorporate this prior information into the subsequent modelling process. The most common approach is to measure the stability of each feature individually, for example by calculating the signal-to-noise ratio or to determine the correlations between groups obtained using different parameterizations, e.g. the same objects imaged twice using different resolutions^18^. Using this information, only the most stable features are kept and the others are removed. The obvious drawback of this approach is that it removes potentially useful information from the main study based on the information from the side experiments (see Fig. 1A): some features might be noisy but still relevant for the prediction and, on the contrary, other features might be stable but contain no relevant information. This is a common property of filtering-based feature selection and thus is independent of the applied threshold and stability criterion used in a radiomics study^19,20^.

In order to overcome this limitation, we suggest incorporating the additional side study information into the main study by creating an augmented dataset. The current approach of removing unstable features actually removes the information available in the dataset (see Fig. 1A). As an alternative, we propose that artificial samples be created based on the main study and the findings of the side experiments. The new augmented data set then naturally combines the original information with the feature stability deduced from the side experiment, thus increasing the available information (see Fig. 1B). This gives the user the option to employ more powerful embedded feature selection approaches when performing the main study.

## Methods and Materials

### Setting

In this study, each observation is represented by a feature vector ${{\boldsymbol{x}}}_{i}$ that consists of $n$ different features ${x}_{i,j};j\,\in 1\ldots n$. To a certain degree, the value of each feature is affected by a confounding variable $v$, i.e. ${x}_{i,j}(v)$. We assume that there is a main study with $m$ observations where the confounding variable has a fixed value ${{\boldsymbol{x}}}_{i}^{M}(v=\,{v}_{1})$. In order to prevent models derived from the main study from being affected by the confounding variable, a side study which includes observations ${{\boldsymbol{x}}}_{i}^{S}(v=[{v}_{1},\,\ldots {v}_{k}])$ with different values of the confounding variable is used. However, only the main study contains information about the target variable $y$. Other studies that contain information about the influence of the confounding variable $y$ are usually small and might miss the target information.

We have compared four different methods. The first one is referred to as “Oracle” and makes use of a main study that does include the information on the confounding variable. While this is often not possible for real studies, it gives a baseline for assessing the effects of the confounding variable. Second is the “Simple” approach. In this approach, the additional knowledge gained by the side study is completely ignored and models are built using the original main study data. The third approach, “Feature Filtering”, is currently considered state-of-the-art. Finally, as the fourth option, we evaluated the “Proposed” method that we have termed “Data Augmentation for Information Transfer” (DAFIT). We will discuss the last two options in the next sections in more detail.

### Feature filtering

Different filtering approaches are available and are used in radiomic studies. We selected the two approaches that are commonly used and selected the method based on the available data. The first method was applied if each observation (i.e patient) has been measured at least two times with varying confounding variable values, i.e. if paired data were available. In this setting, a feature was kept if the measurements for the groups correlated to a certain degree. We used the concordance correlation coefficient (CCC)^21^ to determine the agreement between the two groups. CCC is often used in conjunction with a threshold ${\tau }_{CCC}\,$in radiomics studies and gives values between 0 (no agreement) and 1 (complete agreement). Features with CCC-values below ${\tau }_{CCC}$ were considered unstable and were removed from further analysis. Selecting a ${\tau }_{CCC}$ value is open to interpretation. 0.8 is a common value that is used in many studies and, we have also used it in our experiments here.

CCC is only used if the data available are paired. Otherwise, it is more common for radiomics studies to be split into two groups based on the confounding variable value. Features in which both groups are statistically significantly different are discharged. For this, a significance test is conducted and all features with p-values below the threshold ${\tau }_{test}$ are removed from the analysis. A common combination is the uncorrected t-test and ${\tau }_{test}=0.05$, which we have used in our experiments. In this case, the t-test was not used to determine significance and ${\tau }_{test}\,$was seen as a probability threshold.

### Information enhancement

The fourth option that we evaluated is the proposed idea to enhance the available data from the main study by sampling augmented data. Instead of directly using the observations of the main study ${{\boldsymbol{x}}}^{M}$, augmented observations ${{\boldsymbol{x}}}^{A}=\Theta ({{\boldsymbol{x}}}^{M})$ were used. For our experiments, we chose to generate two augmented observations from each observation in the main study. The transformation function $\Theta$ mapped the information obtained from the side studies onto the available data.

The feature calculation process is often complex and the results depend on many factors. Therefore, the influence of confounding variables could be seen as additional noise added to the true value. A common assumption is that the noise follows a normal distribution. We therefore chose to model the transformation function as follows:

1 $$\Theta ({\boldsymbol{x}}\,|\mu ,\,{\sigma }^{2})\,={\boldsymbol{x}}+{\mathscr{N}}(\mu ,\,{\sigma }^{2})$$

with random observation ${\mathscr{N}}(\mu ,\,{\sigma }^{2})$ drawn from a normal distribution. The mean $\mu$ and variance ${\sigma }^{2}$ were estimated using the data from the corresponding side study. We used two different methods for this, depending on whether the observations from the side study were paired or not.

For paired data, we assumed that the difference between the groups was solely due to the confounding variables. Thus, $\mu$ and ${\sigma }^{2}$ were calculated as the mean and variance, respectively, of the difference between the paired observations. This was not possible for unpaired data. Instead, the observations were grouped into two groups based on the value of the confounding variable. Thus, $\mu$ was the set to the mean difference between the mean values of both groups. Similarly, the variance was calculated as the accumulated variance of both groups using the Bienaymé formula.

### Experiments

All experiments were performed in accordance with the relevant guidelines and regulations. We compared the influence of the four previously mentioned methods and focused on two aspects: the performance of the obtained model and the accuracy of the performance estimation measured, based on biased data. For this, we repeatedly trained multiple classifiers in the discussed methods using two datasets that contained different confounding variables. Using a test set that did not contain this bias allowed the effect of the applied method to be estimated.

Two datasets were used for the evaluation. A synthetic dataset with a known ground truth was created. The training data for the two-class problem was created by randomly generating 150 observations per class. This number of observations was chosen to simulate the typical size of a radiomics study. Each observation consisted of 4000 features with the feature value drawn from a randomly determined normal distribution. The normal distributions were only different between both classes for 20 features, i.e. only 20 of the features were truly meaningful. To simulate the effect of a confounding variable shift, the test set was created to be similar to the training data, but with 500 samples per class. The test set was bigger to reduce the influence of random effects on the evaluation. Also, each feature was shifted according to a normal distributed random value. The script used to generate this dataset can be found in the supplementary information.

**Figure 2:**
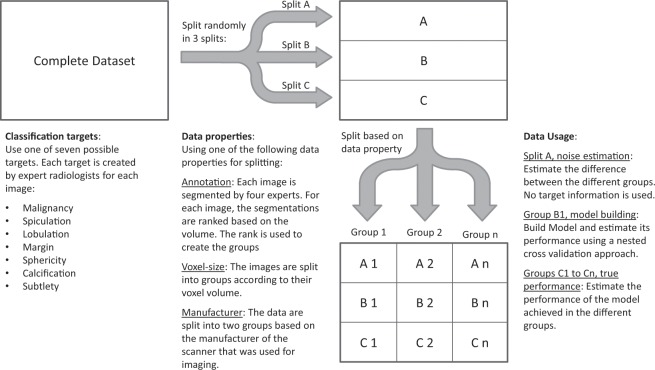
Visualization of the dataset and the usage for this paper. The dataset contains multiple possible classification targets. It is randomly split into different splits A, B, C and then grouped according to one of the three data properties which are used as the confounding variable. Split A simulates a side study, containing information about the confounding variables but not about the target. Group B1 simulates the main study, containing information about the target but not about the confounding variable. Finally, the groups obtained from Split C are used to evaluate the model on groups with different values for the confounding variable.

Besides the synthetic dataset, a publicly available dataset of 1018 CT scans from 1010 patients with lung nodules (Lung Image Database Consortium LIDC-IDRI^22^ from “The Cancer Imaging Archive”^23^) was also used. Data was collected under appropriate local IRB approvals at each of the seven academic institutions (Weill Cornell Medical College, University of California, Los Angeles, University of Chicago, University of Iowa, University of Michigan, MD Anderson Cancer Center, and Memorial Sloan-Kettering Cancer Center); more details are available on this in^22^. This dataset is freely available to browse, download, and use for commercial, scientific and educational purposes as outlined in the Creative Commons Attribution 3.0 Unported License. It is unique in its size and heterogeneity in terms of imaging parameters as well as in tis availability of multiple spatial annotations from different experts for each lesion. Various characteristics that can be used as prediction targets (see Fig. 2) are provided for each lesion. A more detailed description of the classification targets that were used is given in the supplementary information. Based on the spatial annotations, we calculated 435 radiomics features using MITK Phenotyping^24,25^, thus obtaining a feature vector for each nodule (feature list is shown in the supplementary information). The data were split into three groups for noise estimation, model building, and validation and were grouped according to their confounding variables (see Fig. 2). The artificial reduction of this rich dataset allows common situations where information is spread over multiple datasets to be simulated. In order to be able to increase the size of the data set for additional follow-up experiments, we only used approximately 2/3 of the nodules which had four spatial annotations available (1100 observations) for most experiments.

We used three different classification algorithms for each experiment, namely Random Forest (RF), Gradient Boosting Machine (GBM), and logistic regression (LASSO). Using a cross-validation approach, we split the available training data into test sets and training sets. The former was used to estimate the performance of the model trained on the latter. Nested cross-validation of the training set was used for classifier tuning. The whole procedure was repeated multiple times. We measured the prediction quality of the obtained model using the area under the Receiver Operating Characteristic curve (AUC). Besides the actual model performance, the accuracy of the prediction of the model performance was assessed by reporting the difference between the estimated model performance obtained on the test set and the lowest performance obtained from groups C1 to Cn.

The “Oracle” and “Simple” approaches were created by ignoring split A. For the “Oracle” approach, the whole split B was used during model building, meaning that the training data included the confounding effect. Therefore, there were more training observations during model building for the “Oracle” approach, which could give this approach a slight advantage. We decided to take this route because this approach is included as a reference rather than as alternative approach. Only group B1 was used as raining data for the “Simple” approach, so there is no information whatsoever on the confounding effect during the training process. The evaluations of these approaches did not differ from those of the others; the model and estimation performance was also determined on the C1 to Cn subgroups.

## Results

### Model accuracy and prediction quality

**Figure 3:**
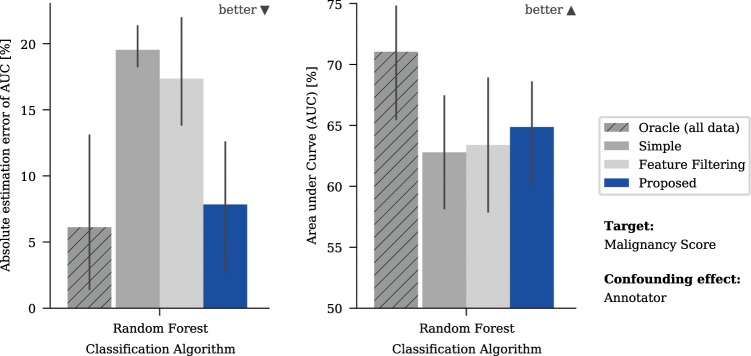
Mean absolute estimation errors (AEE) and mean performance predicting malignancy with annotation as a confounding variable measured using area under curve (AUC). The absolute estimation error is defined as the absolute difference between the performance estimated with five-fold cross-validation and the minimum performance obtained on any left-out test set. The error bars give the 95% confidence interval based on bootstrapping. Note the y-axis for the AUC-plots has an offset to show the relevant areas.

**Figure 4:**
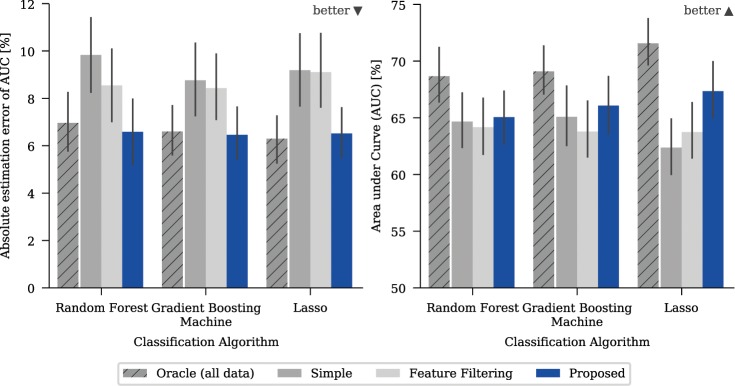
Mean absolute estimation errors (AEE) and mean performance measured with area under curve (AUC) for different strategies and a classifier on a real-world dataset. The absolute estimation error is defined as the absolute difference between the performance estimated with five-fold cross-validation and the minimum performance obtained on any left-out test set. The error bars give the 95% confidence interval based on bootstrapping. Note that the y-axis for the AUC-plots has an offset to show the relevant areas.

We first evaluated the accuracy of the model and the quality of the prediction made on the training data for different methods in order to incorporate the additional knowledge using the available real dataset. Figure 3 shows the results for the confounding variable “Annotation” and “Malignancy” as prediction target, Fig. 4 shows the aggregated results of multiple runs for the various targets and confounding variables listed in Fig. 2.

For the confounding variable “Annotation” we tested whether the differences between the features were normally distributed using the Shapiro-Wilk test and corrected for multiple testing using the Bonferroni adjustment. Out of 435 features, 322 (74%) were normally distributed (p < 0.00011).

The method used to incorporate the information from side studies does have a significant effect on both the model and the prediction accuracy. Naturally, the best option is to have the confounding variable included in the main study (“Oracle”) which gave the optimum baseline for all cases. The naïve approach of ignoring the confounding variable (“Simple”) tended to give stronger models than limiting the available features (“Feature Filtering”) (Fig. 4, right) but could lead to significantly overestimated model accuracy (Fig. 4, left). For the confounding variable “Annotation”, which has the most impact on the model quality, the predicted model accuracy was nearly 20% off the true performance for “Simple” in some cases, for example when predicting malignancy with random forest (Fig. 3). The proposed algorithm allows prediction of the model accuracy to be achieved that is close to that obtained for the “Oracle” (Fig. 4, left). It also elicits the strongest models of the three evaluated methods for all targets, except for “Calcification” (Fig. 4, right). The reported effects were independent of the amount of data that had been used and were also observed when more data was used. In additional experiments, we tried to vary the thresholds used for “Feature Filtering”. This influenced the trade-off between model power and accuracy of the model power estimation, however quantifying the tradeoff is difficult and highly dependent on the actual task. (for more detailed results, see the supplementary information)

We tested which features were used for the “Oracle” model given the confounding variable “Annotation”. The feature importance was taken from the random forest model and the cut-off for the relevant features, distinguishing between important and unimportant features, was manually determined based on the importance plot. Between 18 and 28 features were important for each classification target. Of those features, between 0% and 85% were stable according to the “Filtering” approach. A closer inspection for the target “Sphericity”, where none of the important features were considered stable, showed that the features selected in the “Oracle” are meaningful, with most of them being volumetric features. Consequently, the “Feature Filtering” approach mainly relies on textural features, resulting in an impairment in performance of more than 5% and a reduced prediction of the model performance compared to the oracle approach.

**Figure 5:**
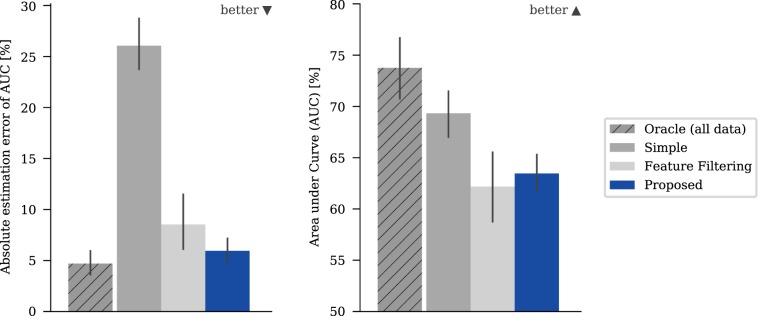
Mean absolute estimation errors (AEE) and mean performance measured with area under curve (AUC) for different strategies on a synthetic dataset. The results of the different classifiers (Random Forests and Gradient Boosting Machine) have been fused. The error bars give the 95% confidence interval based on bootstrapping. Note that the y-axis for the AUC-plots has an offset to show the relevant areas.

The experiments were repeated using the synthetic generated dataset, this time using the known distribution of the feature noise. We limited the experiments and only used the tree-based classifiers (RF and GBM). The results were similar to those obtained from the previous experiment (Fig. 5) but showed an even stronger overestimation of the classifier performance for the “Simple” approach. While “Simple” outperformed “Proposed”, using “Simple” is dangerous due to the high level of estimation error. We believe that the improved performance of the “Simple” approach was achieved by ignoring the feature stability completely, thus enabling us to select the most informative features even if they were unstable. Further analysis showed that on average, 676.5 remaining features (16.9% of all features) were used in the “Feature Filtering” approach. Only 3.7 (18.4%) of 20 defined meaningful features were selected on average, indicating that a large number of meaningful features had been ignored. Based on the feature importance, on average 2.7 and 9.3 meaningful features were found within the 20 most important features in the “Feature Filtering” and “Proposed” approaches respectively. Similarly, on average the first 2.2 and 6.4 most important features were meaningful for the “Feature Filtering” and “Proposed” approaches, respectively. A closer inspection of feature importance showed that models based on the “Feature Filtering” approach tended to rely on one or two features, resulting in a higher level of noise sensitivity within these features. Repeating the experiments again using a skewed normal distribution instead of a normal one showed that the proposed approach is not sensitive to skewed data distributions (see supplementary information for more details).

### Effect of the artificially increased sample size

**Figure 6:**
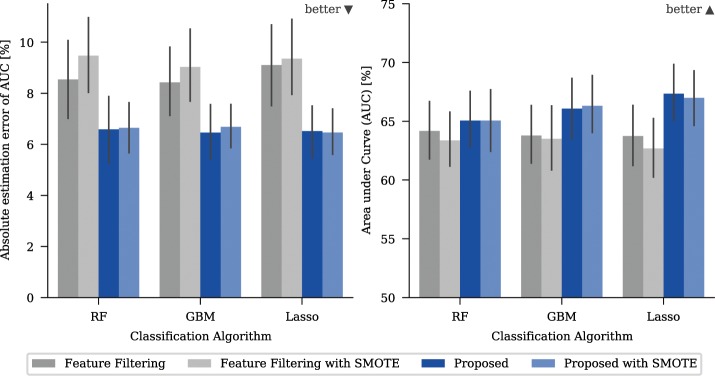
Mean absolute estimation error (AEE) and mean performance measured with area under curve (AUC). The plots show the results obtained for different strategies with and without using additional random data generation with SMOTE algorithm. The error bars give the 95% confidence interval based on bootstrapping. Note that the y-axis for the AUC-plots has an offset to show the relevant areas.

The enhancing strategy of the proposed method is increasing the number of samples available for the training. To ensure that the improved performance of the proposed method is due to the successful inclusion of information in the modelling process rather than the increased sample count, we evaluated whether performance improved if more artificial samples were generated. In order to do this, we compared the previous results to those of a second run where the data had been oversampled using the SMOTE algorithm^26^ prior to the training. This is a common approach for radiomics studies and the best oversampling technique according to Gabyris *et al*.^27^ (Fig. 6).

The results showed that the model and prediction performance of the filtering approach was worse if additional artificial samples were used. SMOTE improved the feature filtering results in only one of the nine combinations of possible confounding variables and classifiers. The results for the proposed method are less conclusive, although the model power and estimation accuracy improved in six of nine cases when SMOTE was not used. Even if additional, artificial samples were used, the proposed method still gave better results compared to the filtering approach. The results obtained from using synthetic datasets of different sizes gave similar results.

### Effect of combining filtering and augmentation

**Figure 7:**
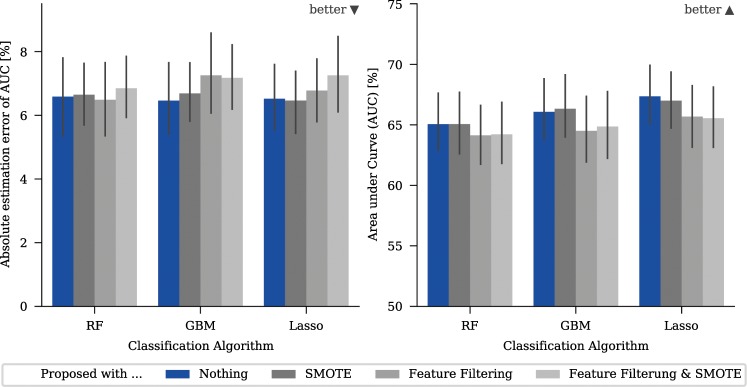
Mean absolute estimation error (AEE) and mean performance measured with area under curve (AUC) for real-world datasets. The diagrams show the effect of combining the filtering approach with the proposed algorithm. Note that the y-axis for the AUC-plots has an offset to show the relevant areas.

The proposed method and filtering can be used together. Since both are useful and help to reduce the unwanted effects of confounding variables, it is useful to look at whether combining both is more powerful than using a single approach. To test this, we implemented this approach and compared the results obtained with the original version of the proposed algorithm (Fig. 7), both with and without additional sampling. To achieve this, we filtered the features according to the “Filtering” approach first and then used the remaining features for the “Proposed” approach.

Using a combination of feature filtering and the proposed algorithm elicited better results in terms of model accuracy and prediction accuracy compared to a pure filtering approach. However, the results were slightly worse than those obtained using the proposed algorithm alone (see Fig. 7). Repeating the results using the synthetic dataset showed similar results; again, the combination of both approaches outperformed pure feature filtering, but showed no clear improvement compared to the proposed algorithm alone.

## Discussion

Side studies can help us to understand the stability of radiomics features and are therefore used in various advanced radiomics studies in order to obtain more stable signatures. Our experiments have demonstrated the effects of side information on the performance and stability of the models obtained. The quality of the model and the quality of the model performance prediction were demonstrably shown to be improved by the proposed “Data Augmentation for Information Transfer” (DAFIT) approach. This held true when the approach was compared to common oversampling strategies and different dataset sizes. The results of this study also show the importance of controlling for confounding variables in radiomics studies. While the model performances were roughly on par with models built from filtered data, the actual estimation of model performance could be off by nearly 20% without correction for confounding variables. This is especially alarming because no constant offset was observed – some estimates were close to the achieved performance and others were not, leading to a situation wherein the estimated model performance could not be used as an indicator for the final performance. As a consequence, inferior models could be selected, reducing the final performance. Even worse, overoptimistic estimates could lead to clinical decisions that are unduly influenced by the radiomics model.

The main advantage of the proposed augmentation-based technique in comparison to the filtering-based methods is that it can be combined with feature selection methods that are not agnostic towards the actual target variable of the study. Filtering-based approaches can only consider the level of noise in a feature, not its information content in terms of the target. Features that contain relevant information for the model may therefore no longer be available in the modelling process. As reported in the experiment section, the combination of the confounding variable “Annotation” and the target “Sphericity” is an example of such a case. Here, most volumetric features are considered unstable; however, these are the features that describe the shape and are therefore best suited for predicting the sphericity of the lesion. Consequently, the filtering-based model relies on texture features to describe the shape of the lesion and performs far worse than the oracle. With data augmentation, it is not only possible to use target-aware filtering methods, but also to use wrapping or embedded feature selection methods which combine feature selection and model building, which tend to yield better performance^19,20^. As a consequence, the DAFIT-model used several of the volumetric features and outperformed the filtering model for the previously described case.

It seems that LASSO-based classification methods benefit the most from the proposed approach. While the reasons for this are not completely clear, we believe that this is because the LASSO classifier enables better adaptation for the uncertainty in the training data by weighting the individual features. On the contrary, tree-based classifiers usually use hard thresholds for their decisions. We believe that this makes it more difficult to represent data uncertainty, but further research is needed to clarify this in detail.

The stability of features can be affected to a great extent by various confounding variables. Recent research has shown that some confounding variables have a greater impact on feature stability. For example, the placement of a region of interest has a bigger effect than imaging parameters^7,9^, and this has been validated by our experiments. However, the actual influence of a confounding variable on a specific feature depends on many parameters such as imaging modality, imaging parameters, the cancer entity, or the feature family.

While there is a significant amount of research on the magnitude of an effect caused by confounding effects, we do not know of any study that has looked at the distribution of these individual effects. Without knowing the true distribution of the effects, we decided to model them as normally distributed. In fact, when looking at the effects caused by different annotations, we found that this is true for many features but not for all features. Ergo, the assumption we used for generating the artificial samples may be invalid for some features, but we found that this approach is still beneficial in the cases that we tested. However, DAFIT can be further improved by adapting the noise model to the true distribution. This requires additional research on the distribution of the effects – not solely on the magnitude.

In general, our study confirmed the current interest in radiomics feature stability and the recommendation of the Radiomics Quality Score^16^. Although the models and predictions of the proposed method are better than those obtained with feature filtering, we found that feature filtering can still be an effective method in preventing fitting to confounding variables. This emphasis the importance of external validation datasets for radiomics studies. Additional datasets reduce the chance that a reported performance is biased by a confounding variable that is still unknown or has been forgotten in the current study.

We intentionally designed our study to be general and reflect common situations in radiomics research. For this, we used a unique dataset that allowed us to generate 21 sub-datasets with different combinations of prediction targets and confounding variables, preventing overfitting of our finding to a single situation. Further, each experiment was repeated multiple times, leading to hundreds of different results. While this design allows both the effect of different confounding variable and the influence of methods to reduce this effect to be quantified, it is clearly not suited for developing a radiomics marker. For example, each model obtained for this study was only optimized for a single confounding variable and some important confounding variables such as reconstruction kernel are not considered at all. As a consequence, the reported models or model performances should not be interpreted as performances for clinically applicable models. This is also true for the performance of the oracle approach, which is neither evaluated on an independent test set nor corrected for important confounding variables.

A limitation of this study is the use of two datasets: one with patient data and one that was synthetically created. The three real confounding effects were analyzed based on these datasets – other confounding variables with strong effects such as reconstruction kernel and contrast concentration are not examined within this paper. The main findings of this study, that is the importance of controlling for confounding variables and the effectiveness of the proposed DAFIT approach, are not specific for individual confounding variables. We therefore believe that the findings will hold true for other confounding variables as well, however further research is needed to validate this assumption.

In addition, it is possible to use DAFIT to correct for different side effects at once. However, we did not evaluate this in this paper but are planning to execute this separately. Another limitation is that we only compared target-agnostic filtering methods with embedded feature filtering methods. Possible extensions could include combining the proposed augmentation with target-aware filtering methods, transferring target information to the side studies instead of extending the size of the main study or using a different classifier for the model building process. Furthermore, a combination with batch normalization methods that account for batch-wide differences, e.g. ComBat^28,29^, could be evaluated since these elements address different challenges and do not contradict the current study design.

## Conclusions

Our study highlights the importance of controlling for confounding variable in radiomics studies. Not doing so can lead to significantly overestimated model performances. We also propose a novel method called “Data Augmentation for Information Transfer” (DAFIT) for controlling for confounding variables. Our experiments, based on three confounding variables and 21 sub-datasets, show evidence that the proposed method has compelling advantages over simply ignoring confounding variables or filtering out unstable features when feature stability information from an independent experiment is incorporated into a radiomics study.

### Ethics approval and consent to participate

This work makes only use of publicly available data for which appropriate local IRB approval has been obtained^22^.

## Supplementary information

Supplementary Information.
