# Supplementary material for: Optimal Statistical Incorporation of Independent Feature Stability Information into Radiomics Studies
Source: Sci Rep. 2020 Jan 20;10:737. doi: 10.1038/s41598-020-57739-8 (PMC6971266; doi:10.1038/s41598-020-57739-8)
Supplement: Supplementary file 1 — Supplementary Information. [file 41598_2020_57739_MOESM1_ESM.pdf]

Optimal statistical incorporation of independent feature stability information into radiomics studies

Dr. Michael Götz* [1,2] , PD. Dr. Klaus H. Maier-Hein[1, 2]

1. Division of Medical Image Computing, German Cancer Research Center (DKFZ) Heidelberg, Germany
2. National Center for Tumor Diseases (NCT), Heidelberg, Germany

Corresponding Author:

Michael Götz
E230, Medical Image Computing
German Cancer Research Center (DKFZ) Heidelberg
Im Neuenheimer Feld 280
69120 Heidelberg
Germany

E-Mail: m.goetz@dkfz-heidelberg.de

Inhalt

Optimal statistical incorporation of independent feature stability information into radiomics studies 1

A0 Experiment on different skew factors 3

A1 Result listed for different confounding variables 3

Annotation: 3

Manufacturer: 4

Voxelsize: 4

A2 Result listed for different targets 4

Malignancy: 4

Spiculation: 5

Lobulation: 5

Margin: 5

Sphericity: 5

Calcification: 6

Subtlety: 6

A3 Grid of estimation accuracy w.r.t. confounding effect and target 6

A4 Grid of area under curve w.r.t. confounding effect and target 8

A5: Script to generate the artificial dataset 10

A6 Description of targets: 11

A7 List of calculated features 11

A8 Feature Noise Distributions for Annotator 20

A9 Feature Noise Distributions for Manufacturer 24

A10 Feature Noise Distributions for Voxelsize 28

A11 Distribution of features w.r.t. subclasses 33

A0 Experiment on different skew factors

In order to research the impact of skewed distributions on the proposed method DaFIT, we repeated the synthetic experiments. This time we added noise drawn from a skewed normal distribution instead of normally distributed noise. The noise is calculated using the package “skewnorm” from “scipy.stats”, and we tested two different values for the skew factor, namely two and four. We did not change the noise model used for DaFIT, still assuming an unskewed normal distribution for the noise. The experiments were carried out multiple times to account for the randomness in the experiments.

The results are shown in the figure below. No clear trend for better/worse prediction due to the skewewing can be seen, however it seems that the resulting classifier are slightly better if trained on the skewed data.

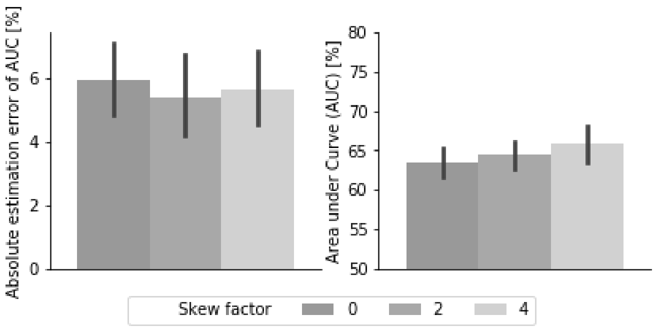

A1 Result listed for different confounding variables

Mean absolute estimation errors (AEE) and mean performance measure with Area Under Curve (AUC) for different strategies and classifier on a real-world dataset. The absolute estimation error is defined as the absolute difference between the performance estimated with five-fold cross-validation and the minimum performance obtained on any left-out test set. The error bars give the 95% confidence interval based on bootstrapping. Be aware that the y-axis for the AUC-plots has an offset to show relevant areas.

Annotation:

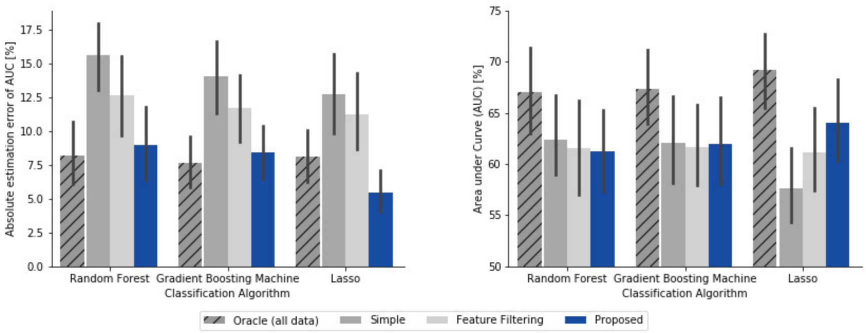

Manufacturer:

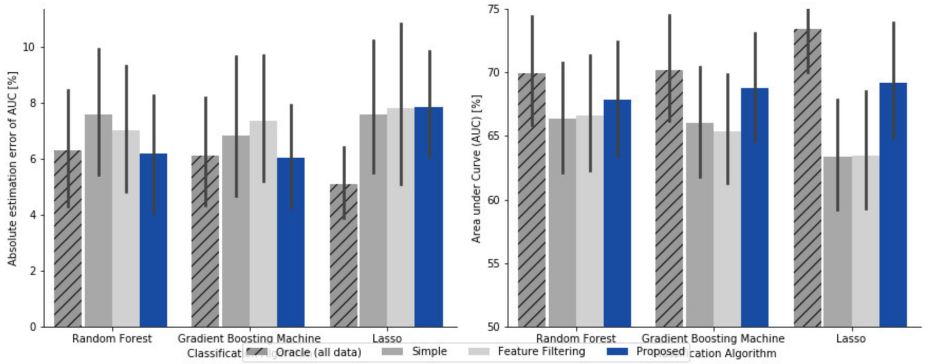

Voxelsize:

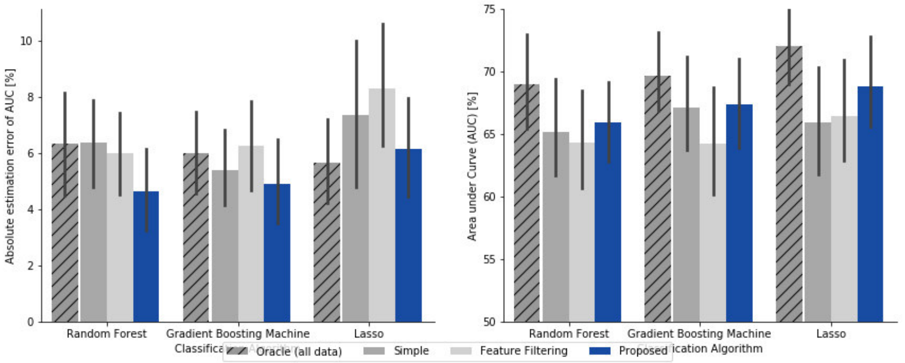

A2 Result listed for different targets

Mean absolute estimation errors (AEE) and mean performance measure with Area Under Curve (AUC) for different strategies and classifier on a real-world dataset. The absolute estimation error is defined as the absolute difference between the performance estimated with five-fold cross-validation and the minimum performance obtained on any left-out test set. The error bars give the 95% confidence interval based on bootstrapping. Be aware that the y-axis for the AUC-plots has an offset to show relevant areas.

Malignancy:

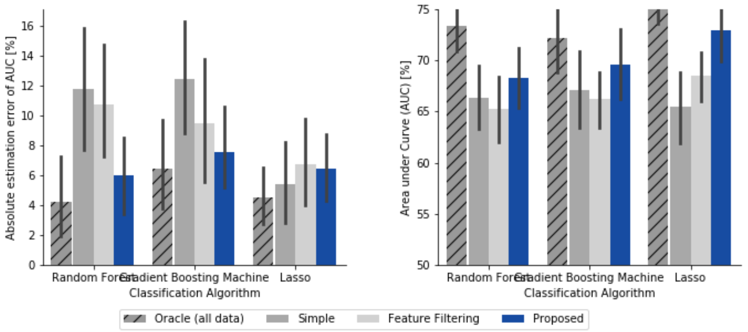

Spiculation:

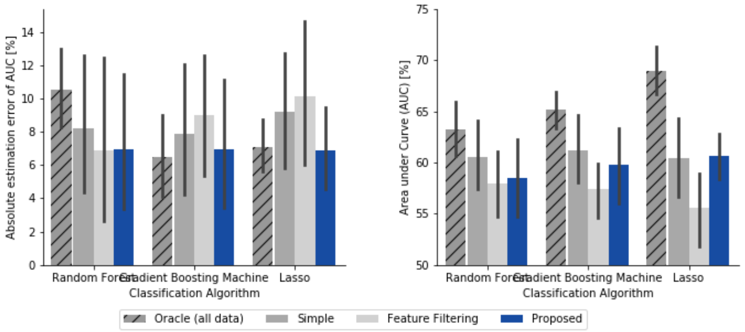

Lobulation:

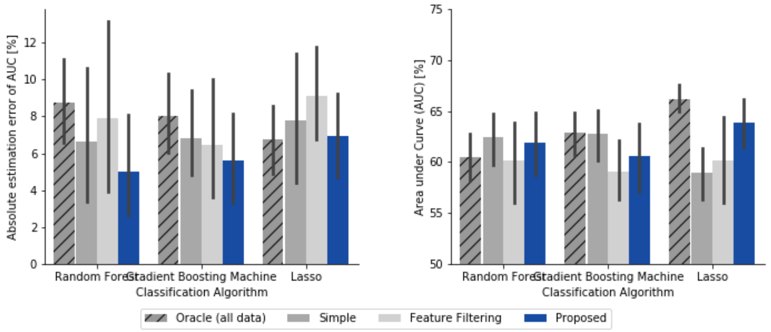

Margin:

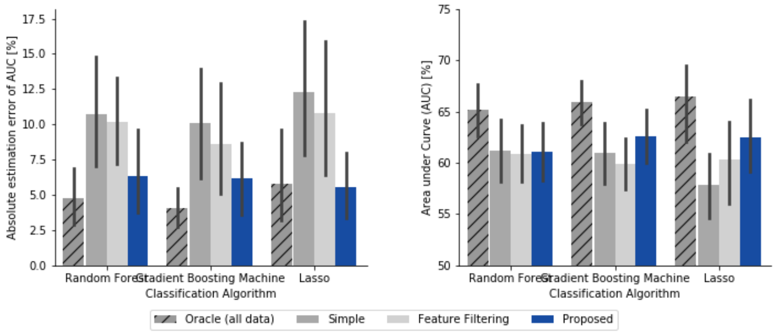

Sphericity:

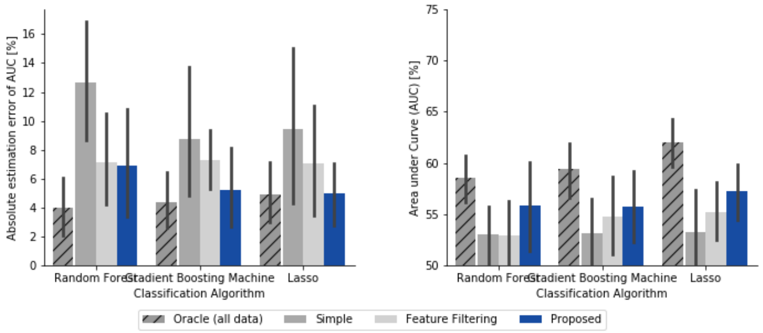

Calcification:

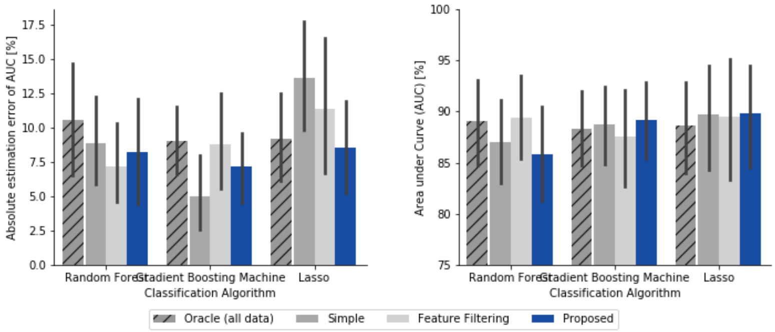

Subtlety:

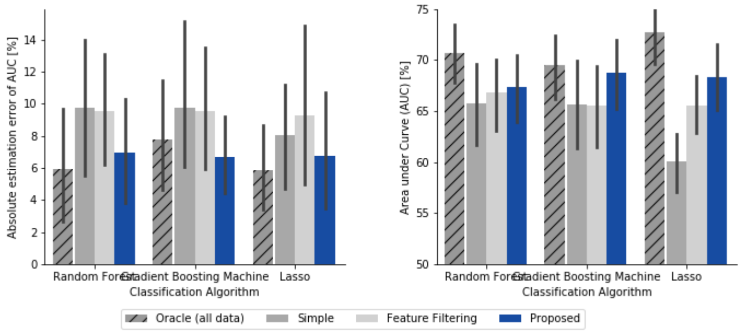

A3 Grid of estimation accuracy w.r.t. confounding effect and target

Mean absolute estimation errors (AEE) for different strategies and classifier on a real-world dataset. The absolute estimation error is defined as the absolute difference between the performance estimated with five-fold cross-validation and the minimum performance obtained on any left-out test set. The error bars give the 95% confidence interval based on bootstrapping.

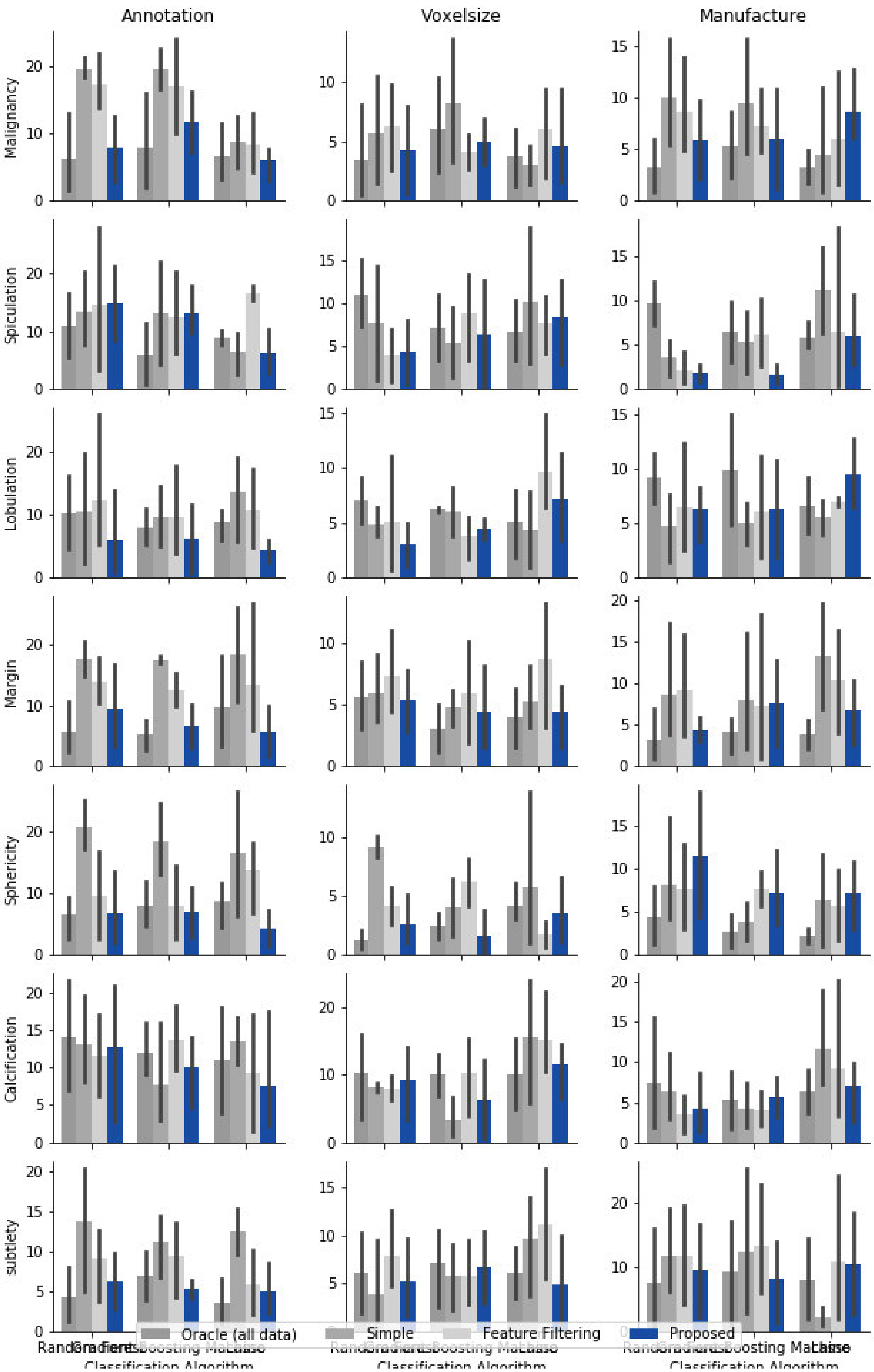

Annotation
Voxelsize
Manufacture
Malignancy
Spiculation
Lobulation
Margin
Sphericity
Calcification
subtlety
Oracle (all data)
Simple
Feature Filtering
Proposed
Classification Algorithm

A4 Grid of area under curve w.r.t. confounding effect and target

Mean performance measure with Area Under Curve (AUC) for different strategies and classifier on a real-world dataset. The error bars give the 95% confidence interval based on bootstrapping. Be aware that the y-axis for the AUC-plots has an offset to show relevant areas.

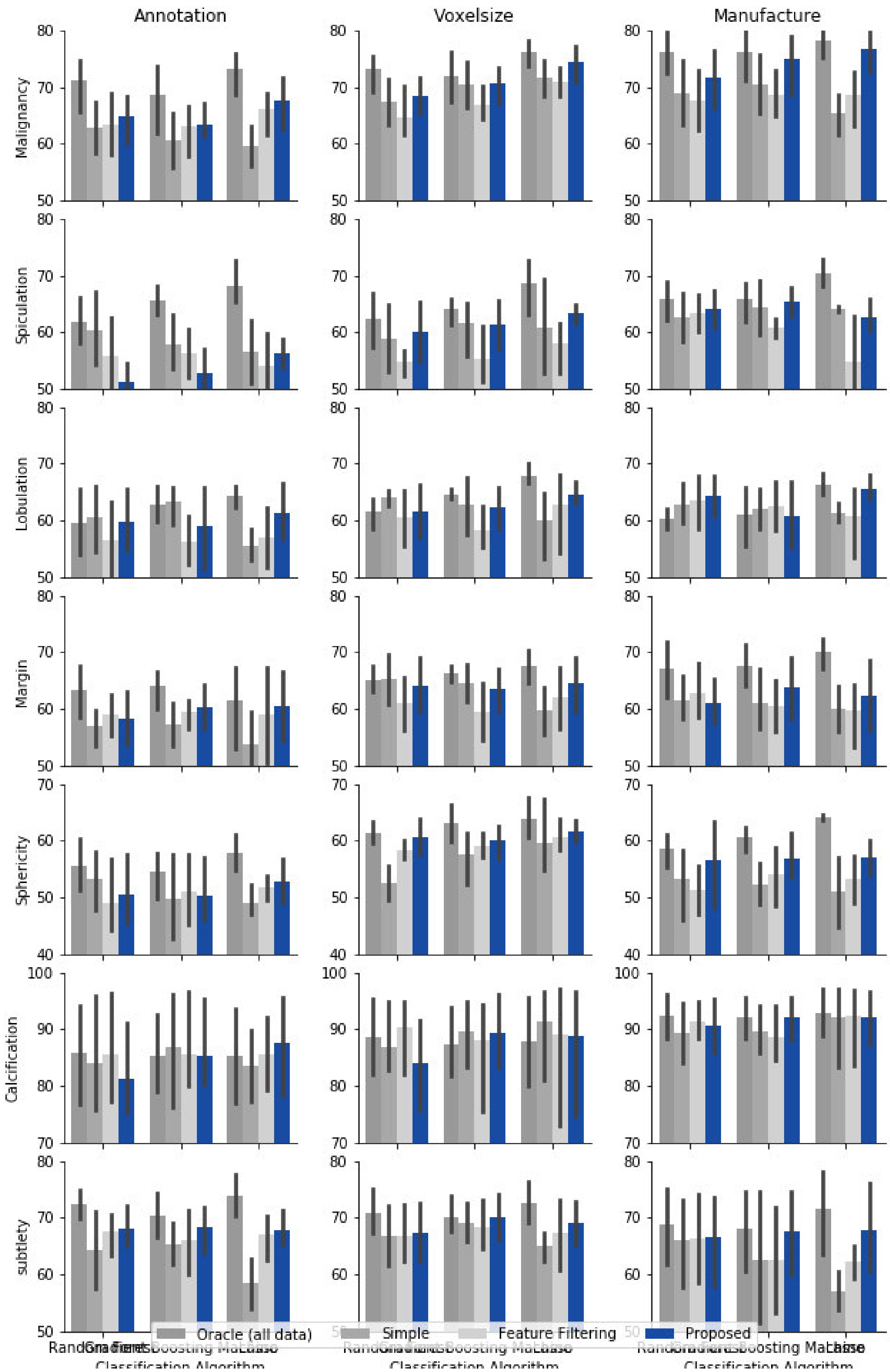
Annotation
Voxelsize
Manufacture
Malignancy
Spiculation
Lobulation
Margin
Sphericity
Calcification
subtlety
Oracle (all data)
Simple
Feature Filtering
Proposed
Classification Algorithm

A5: Script to generate the artificial dataset

```
# -*- coding: utf-8 -*-
"""
Created on Tue Sep 17 10:59:35 2019

@author: Michael Goetz

This script generates the synthetic dataset used for testing DaFIT.
The important variables are:

trining_samples_noisy
training_samples_oracle_noisy
training_labels

rescan_samples1
rescan_samples2

validation_samples_noisy
validation_labels
"""

import numpy.random as rand
import numpy as np
from scipy.stats import skewnorm

################################################################
#
#  Parameter
#
################################################################
no_of_training_samples=150
no_of_validation_samples=500
meaningfull_variables=20
overall_variables=4000
skew_factor_a=2
range_of_feature_mean=[-.5,.5]
range_of_feature_std=[0.3,2]
range_of_noise_std=[0.3,2]
seed=None
skew_factor=0


if seed is not None:
    rand.seed(seed)
loc=skewnorm.mean(skew_factor)
scale=skewnorm.std(skew_factor)

class_A_means=rand.uniform(range_of_feature_mean[0], range_of_feature_mean[1], meaningfull_variables)
class_B_means=rand.uniform(range_of_feature_mean[0], range_of_feature_mean[1], meaningfull_variables)
class_A_std=rand.uniform(range_of_feature_std[0], range_of_feature_std[1], meaningfull_variables)
class_B_std=rand.uniform(range_of_feature_std[0], range_of_feature_std[1], meaningfull_variables)
class_C_means=rand.uniform(range_of_feature_mean[0], range_of_feature_mean[1], overall_variables-
meaningfull_variables)
class_C_std=rand.uniform(range_of_feature_std[0], range_of_feature_std[1], overall_variables-
meaningfull_variables)

noise_mean=np.zeros(overall_variables)
noise_std=rand.uniform(range_of_noise_std[0], range_of_noise_std[1], overall_variables)

############ Generate Noisy Training Data #################################

training_samples_A=rand.normal(class_A_means, class_A_std, (no_of_training_samples,meaningfull_variables))
training_samples_B=rand.normal(class_B_means, class_B_std, (no_of_training_samples,meaningfull_variables))
training_samples_C1=rand.normal(class_C_means, class_C_std, (no_of_training_samples,overall_variables-
meaningfull_variables))
training_samples_C2=rand.normal(class_C_means, class_C_std, (no_of_training_samples,overall_variables-
meaningfull_variables))

training_samples_A=np.concatenate((training_samples_A,training_samples_C1),axis=1)
training_samples_B=np.concatenate((training_samples_B,training_samples_C2),axis=1)
training_samples=np.concatenate((training_samples_A, training_samples_B))
training_labels=np.concatenate((np.zeros(no_of_training_samples),np.ones(no_of_training_samples)))

training_noise_data=np.zeros((no_of_training_samples*2,overall_variables))
for i in range(overall_variables):
    training_noise_data[:,i]=skewnorm.rvs(skew_factor, size=no_of_training_samples*2, loc=-
loc+noise_mean[i], scale=noise_std[i]/scale)
training_samples_noisy=training_samples.copy()+training_noise_data[0,:]
training_samples_oracle_noisy=training_samples.copy()+training_noise_data


############ Generate Validation Data #####################################

validation_samples_A=rand.normal(class_A_means, class_A_std,
(no_of_validation_samples,meaningfull_variables))
```

```
validation_samples_B=rand.normal(class_B_means, class_B_std,
(no_of_validation_samples,meaningfull_variables))
validation_samples_C1=rand.normal(class_C_means, class_C_std, (no_of_validation_samples,overall_variables-
meaningfull_variables))
validation_samples_C2=rand.normal(class_C_means, class_C_std, (no_of_validation_samples,overall_variables-
meaningfull_variables))

validation_samples_A=np.concatenate((validation_samples_A,validation_samples_C1),axis=1)
validation_samples_B=np.concatenate((validation_samples_B,validation_samples_C2),axis=1)
validation_samples=np.concatenate((validation_samples_A, validation_samples_B))
validation_labels=np.concatenate((np.zeros(no_of_validation_samples),np.ones(no_of_validation_samples)))

validation_noise_data=np.zeros((no_of_validation_samples*2,overall_variables))
for i in range(overall_variables):
    validation_noise_data[:,i]=skewnorm.rvs(skew_factor, size=no_of_validation_samples*2, loc=-
loc+noise_mean[i], scale=noise_std[i]/scale)
validation_samples_noisy=validation_samples.copy()+validation_noise_data


################# Simulated Scan-Rescan Data #############################
rescan_noise_data1=np.zeros((no_of_training_samples*2,overall_variables))
rescan_noise_data2=np.zeros((no_of_training_samples*2,overall_variables))
for i in range(overall_variables):
    rescan_noise_data1[:,i]=skewnorm.rvs(skew_factor, size=no_of_training_samples*2, loc=-
loc+noise_mean[i], scale=noise_std[i]/scale)
    rescan_noise_data2[:,i]=skewnorm.rvs(skew_factor, size=no_of_training_samples*2, loc=-
loc+noise_mean[i], scale=noise_std[i]/scale)
rescan_samples1=training_samples.copy()+rescan_noise_data1
rescan_samples2=training_samples.copy()+rescan_noise_data2
```

A6 Description of targets:

A slightly more detailed description of the classification targets. Those targets were chosen because they are publicly available in conjunction with the LIDC-IDRI dataset.

- Subtlety: How difficult is the lesion to detect?
- Calcification: What is the pattern of calcification, if it is present?
- Sphericity: Describing the shape of the nodule in terms of roundness.
- Margin: Rates how well-defined the margin is.
- Lobulation: The degree of lublation ranging from none to marked
- Spiculation: The extend of speculation
- Malignancy: Subjective guess how likely it is that the nodule is malignant, assumed that the scan originated from a 60-year-old male smoker.

The information about the classification targets is taken from the publication describing the creation process of the LIDC-IDRI dataset.

(Michael F. McNitt-Gray et al. “The Lung Image Database Consortium (LIDC) Data Collection Process for Nodule Detection and Annotation”, in Academic Radiology 2007, doi: 10.1016/j.acra.2007.07.021.)

A7 List of calculated features

Here is full list of all features that are calulcated. The name of each feature follows a fixed set-up: The first part gives the family of the features, for example “Volumetric_Features”. The second part is separated by two double-points: “::” and gives the parameter, if applicable. The third part, after two additional double points gives the actual feature name, for example “Voxel_Volume”.

A more detailed description of each feature can be found in the documentation of MITK Phenotyping: http://mitk.org/wiki/Phenotyping#Documentation_and_Help in the documentation of the corresponding feature family class.

List of all features:

1. Volumetric_Features::::Voxel_Volume
2. Volumetric_Features::::Volume_(voxel_based)
3. Volumetric_Features::::Maximum_3D_diameter
4. Volumetric_Features::::Surface_(voxel_based)
5. Volumetric_Features::::Centre_of_mass_shift
6. Volumetric_Features::::Centre_of_mass_shift_(uncorrected)
7. Volumetric_Features::::Bounding_Box_Volume
8. Volumetric_Features::::Volume_(mesh_based)
9. Volumetric_Features::::Surface_(mesh_based)
10. Volumetric_Features::::Surface_to_volume_ratio_(mesh_based)
11. Volumetric_Features::::Sphericity_(mesh_based)
12. Volumetric_Features::::Asphericity_(mesh_based)
13. Volumetric_Features::::Compactness_1_(mesh_based)
14. Volumetric_Features::::Compactness_1_old_(mesh_based)
15. Volumetric_Features::::Compactness_2_(mesh_based)
16. Volumetric_Features::::Spherical_disproportion_(mesh_based)
17. Volumetric_Features::::Surface_to_volume_ratio_(voxel_based)
18. Volumetric_Features::::Sphericity_(voxel_based)
19. Volumetric_Features::::Asphericity_(voxel_based)
20. Volumetric_Features::::Compactness_1_(voxel_based)
21. Volumetric_Features::::Compactness_1_old_(voxel_based)
22. Volumetric_Features::::Compactness_2_(voxel_based)
23. Volumetric_Features::::Spherical_disproportion_(voxel_based)
24. Volumetric_Features::::PCA_Major_axis_length
25. Volumetric_Features::::PCA_Minor_axis_length
26. Volumetric_Features::::PCA_Least_axis_length
27. Volumetric_Features::::PCA_Elongation
28. Volumetric_Features::::PCA_Flatness
29. Volumetric_Features::::PCA_Major_axis_length_(uncorrected)
30. Volumetric_Features::::PCA_Minor_axis_length_(uncorrected)
31. Volumetric_Features::::PCA_Least_axis_length_(uncorrected)
32. Volumetric_Features::::PCA_Elongation_(uncorrected)
33. Volumetric_Features::::PCA_Flatness_(uncorrected)
34. Curvature_Feature::::Minimum_Mean_Curvature
35. Curvature_Feature::::Maximum_Mean_Curvature
36. Curvature_Feature::::Mean_Mean_Curvature
37. Curvature_Feature::::Standard_Deviation_Mean_Curvature
38. Curvature_Feature::::Skewness_Mean_Curvature
39. Curvature_Feature::::Mean_Positive_Mean_Curvature
40. Curvature_Feature::::Standard_Deviation_Positive_Mean_Curvature
41. Curvature_Feature::::Skewness_Positive_Mean_Curvature
42. Curvature_Feature::::Minimum_Gaussian_Curvature
43. Curvature_Feature::::Maximum_Gaussian_Curvature
44. Curvature_Feature::::Mean_Gaussian_Curvature
45. Curvature_Feature::::Standard_Deviation_Gaussian_Curvature
46. Curvature_Feature::::Skewness_Gaussian_Curvature
47. Curvature_Feature::::Mean_Positive_Gaussian_Curvature
48. Curvature_Feature::::Standard_Deviation_Positive_Gaussian_Curvature
49. Curvature_Feature::::Skewness_Positive_Gaussian_Curvature
50. Curvature_Feature::::Mean_Negative_Gaussian_Curvature
51. Curvature_Feature::::Standard_Deviation_Negative_Gaussian_Curvature
52. Curvature_Feature::::Skewness_Negative_Gaussian_Curvature
53. Curvature_Feature::::Minimum_Minimum_Curvature
54. Curvature_Feature::::Maximum_Minimum_Curvature

55. Curvature_Feature::::Mean_Minimum_Curvature
56. Curvature_Feature::::Standard_Deviation_Minimum_Curvature
57. Curvature_Feature::::Skewness_Minimum_Curvature
58. Curvature_Feature::::Mean_Positive_Minimum_Curvature
59. Curvature_Feature::::Standard_Deviation_Positive_Minimum_Curvature
60. Curvature_Feature::::Skewness_Positive_Minimum_Curvature
61. Curvature_Feature::::Mean_Negative_Minimum_Curvature
62. Curvature_Feature::::Standard_Deviation_Negative_Minimum_Curvature
63. Curvature_Feature::::Skewness_Negative_Minimum_Curvature
64. Curvature_Feature::::Minimum_Maximum_Curvature
65. Curvature_Feature::::Maximum_Maximum_Curvature
66. Curvature_Feature::::Mean_Maximum_Curvature
67. Curvature_Feature::::Standard_Deviation_Maximum_Curvature
68. Curvature_Feature::::Skewness_Maximum_Curvature
69. Curvature_Feature::::Mean_Positive_Maximum_Curvature
70. Curvature_Feature::::Standard_Deviation_Positive_Maximum_Curvature
71. Curvature_Feature::::Skewness_Positive_Maximum_Curvature
72. First_Order::Bins-128_FullImage::Mean
73. First_Order::Bins-128_FullImage::Unbiased_Variance
74. First_Order::Bins-128_FullImage::Biased_Variance
75. First_Order::Bins-128_FullImage::Skewness
76. First_Order::Bins-128_FullImage::Kurtosis
77. First_Order::Bins-128_FullImage::Median
78. First_Order::Bins-128_FullImage::Minimum
79. First_Order::Bins-128_FullImage::Maximum
80. First_Order::Bins-128_FullImage::Range
81. First_Order::Bins-128_FullImage::Mean_Absolute_Deviation
82. First_Order::Bins-128_FullImage::Robust_Mean_Absolute_Deviation
83. First_Order::Bins-128_FullImage::Median_Absolute_Deviation
84. First_Order::Bins-128_FullImage::Coefficient_Of_Variation
85. First_Order::Bins-128_FullImage::Quantile_Coefficient_Of_Dispersion
86. First_Order::Bins-128_FullImage::Energy
87. First_Order::Bins-128_FullImage::Root_Mean_Square
88. First_Order::Bins-128_FullImage::Robust_Mean
89. First_Order::Bins-128_FullImage::Uniformity
90. First_Order::Bins-128_FullImage::Entropy
91. First_Order::Bins-128_FullImage::Excess_Kurtosis
92. First_Order::Bins-128_FullImage::Covered_Image_Intensity_Range
93. First_Order::Bins-128_FullImage::Sum
94. First_Order::Bins-128_FullImage::Mode
95. First_Order::Bins-128_FullImage::Mode_Probability
96. First_Order::Bins-128_FullImage::Unbiased_Standard_deviation
97. First_Order::Bins-128_FullImage::Biased_Standard_deviation
98. First_Order::Bins-128_FullImage::Number_Of_Voxels
99. First_Order::Bins-128_FullImage::05th_Percentile
100. First_Order::Bins-128_FullImage::10th_Percentile
101. First_Order::Bins-128_FullImage::15th_Percentile
102. First_Order::Bins-128_FullImage::20th_Percentile
103. First_Order::Bins-128_FullImage::25th_Percentile
104. First_Order::Bins-128_FullImage::30th_Percentile
105. First_Order::Bins-128_FullImage::35th_Percentile
106. First_Order::Bins-128_FullImage::40th_Percentile
107. First_Order::Bins-128_FullImage::45th_Percentile
108. First_Order::Bins-128_FullImage::50th_Percentile
109. First_Order::Bins-128_FullImage::55th_Percentile
110. First_Order::Bins-128_FullImage::60th_Percentile

111. First_Order::Bins-128_FullImage::65th_Percentile
112. First_Order::Bins-128_FullImage::70th_Percentile
113. First_Order::Bins-128_FullImage::75th_Percentile
114. First_Order::Bins-128_FullImage::80th_Percentile
115. First_Order::Bins-128_FullImage::85th_Percentile
116. First_Order::Bins-128_FullImage::90th_Percentile
117. First_Order::Bins-128_FullImage::95th_Percentile
118. First_Order::Bins-128_FullImage::Interquartile_Range
119. First_Order::Bins-128_FullImage::Image_Dimension
120. First_Order::Bins-128_FullImage::Voxel_Space
121. First_Order::Bins-128_FullImage::Voxel_Volume
122. First_Order_Numeric::Bins-128_FullImage::Mean
123. First_Order_Numeric::Bins-128_FullImage::Variance
124. First_Order_Numeric::Bins-128_FullImage::Skewness
125. First_Order_Numeric::Bins-128_FullImage::Excess_kurtosis
126. First_Order_Numeric::Bins-128_FullImage::Median
127. First_Order_Numeric::Bins-128_FullImage::Minimum
128. First_Order_Numeric::Bins-128_FullImage::05th_Percentile
129. First_Order_Numeric::Bins-128_FullImage::10th_Percentile
130. First_Order_Numeric::Bins-128_FullImage::15th_Percentile
131. First_Order_Numeric::Bins-128_FullImage::20th_Percentile
132. First_Order_Numeric::Bins-128_FullImage::25th_Percentile
133. First_Order_Numeric::Bins-128_FullImage::30th_Percentile
134. First_Order_Numeric::Bins-128_FullImage::35th_Percentile
135. First_Order_Numeric::Bins-128_FullImage::40th_Percentile
136. First_Order_Numeric::Bins-128_FullImage::45th_Percentile
137. First_Order_Numeric::Bins-128_FullImage::50th_Percentile
138. First_Order_Numeric::Bins-128_FullImage::55th_Percentile
139. First_Order_Numeric::Bins-128_FullImage::60th_Percentile
140. First_Order_Numeric::Bins-128_FullImage::65th_Percentile
141. First_Order_Numeric::Bins-128_FullImage::70th_Percentile
142. First_Order_Numeric::Bins-128_FullImage::75th_Percentile
143. First_Order_Numeric::Bins-128_FullImage::80th_Percentile
144. First_Order_Numeric::Bins-128_FullImage::85th_Percentile
145. First_Order_Numeric::Bins-128_FullImage::90th_Percentile
146. First_Order_Numeric::Bins-128_FullImage::95th_Percentile
147. First_Order_Numeric::Bins-128_FullImage::Maximum
148. First_Order_Numeric::Bins-128_FullImage::Interquantile_range
149. First_Order_Numeric::Bins-128_FullImage::Range
150. First_Order_Numeric::Bins-128_FullImage::Mean_absolute_deviation
151. First_Order_Numeric::Bins-128_FullImage::Robust_mean_absolute_deviation
152. First_Order_Numeric::Bins-128_FullImage::Median_absolute_deviation
153. First_Order_Numeric::Bins-128_FullImage::Coefficient_of_variation
154. First_Order_Numeric::Bins-128_FullImage::Quantile_coefficient_of_dispersion
155. First_Order_Numeric::Bins-128_FullImage::Energy
156. First_Order_Numeric::Bins-128_FullImage::Root_mean_square
157. First_Order_Numeric::Bins-128_FullImage::Standard_Deviation
158. First_Order_Numeric::Bins-128_FullImage::Kurtosis
159. First_Order_Numeric::Bins-128_FullImage::Robust_mean
160. First_Order_Numeric::Bins-128_FullImage::Robust_variance
161. First_Order_Numeric::Bins-128_FullImage::Covered_image_intensity_range
162. First_Order_Numeric::Bins-128_FullImage::Mode_index
163. First_Order_Numeric::Bins-128_FullImage::Mode_value
164. First_Order_Numeric::Bins-128_FullImage::Mode_probability
165. First_Order_Numeric::Bins-128_FullImage::Entropy
166. First_Order_Numeric::Bins-128_FullImage::Uniformtiy

167. First_Order_Numeric::Bins-128_FullImage::Number_of_voxels
168. First_Order_Numeric::Bins-128_FullImage::Sum_of_voxels
169. First_Order_Numeric::Bins-128_FullImage::Voxel_space
170. First_Order_Numeric::Bins-128_FullImage::Voxel_volume
171. First_Order_Numeric::Bins-128_FullImage::Image_Dimension
172. First_Order_Histogram::Bins-802_FullImage::Mean_Value
173. First_Order_Histogram::Bins-802_FullImage::Variance_Value
174. First_Order_Histogram::Bins-802_FullImage::Skewness_Value
175. First_Order_Histogram::Bins-802_FullImage::Excess_Kurtosis_Value
176. First_Order_Histogram::Bins-802_FullImage::Median_Value
177. First_Order_Histogram::Bins-802_FullImage::Minimum_Value
178. First_Order_Histogram::Bins-802_FullImage::Percentile_10_Value
179. First_Order_Histogram::Bins-802_FullImage::Percentile_90_Value
180. First_Order_Histogram::Bins-802_FullImage::Maximum_Value
181. First_Order_Histogram::Bins-802_FullImage::Mode_Value
182. First_Order_Histogram::Bins-802_FullImage::Interquantile_Range_Value
183. First_Order_Histogram::Bins-802_FullImage::Range_Value
184. First_Order_Histogram::Bins-802_FullImage::Mean_Absolute_Deviation_Value
185. First_Order_Histogram::Bins-802_FullImage::Robust_Mean_Absolute_Deviation_Value
186. First_Order_Histogram::Bins-802_FullImage::Median_Absolute_Deviation_Value
187. First_Order_Histogram::Bins-802_FullImage::Coefficient_of_Variation_Value
188. First_Order_Histogram::Bins-802_FullImage::Quantile_coefficient_of_Dispersion_Value
189. First_Order_Histogram::Bins-802_FullImage::Entropy_Value
190. First_Order_Histogram::Bins-802_FullImage::Uniformity_Value
191. First_Order_Histogram::Bins-802_FullImage::Robust_Mean_Value
192. First_Order_Histogram::Bins-802_FullImage::Mean_Index
193. First_Order_Histogram::Bins-802_FullImage::Variance_Index
194. First_Order_Histogram::Bins-802_FullImage::Skewness_Index
195. First_Order_Histogram::Bins-802_FullImage::Excess_Kurtosis_Index
196. First_Order_Histogram::Bins-802_FullImage::Median_Index
197. First_Order_Histogram::Bins-802_FullImage::Minimum_Index
198. First_Order_Histogram::Bins-802_FullImage::Percentile_10_Index
199. First_Order_Histogram::Bins-802_FullImage::Percentile_90_Index
200. First_Order_Histogram::Bins-802_FullImage::Maximum_Index
201. First_Order_Histogram::Bins-802_FullImage::Mode_Index
202. First_Order_Histogram::Bins-802_FullImage::Interquantile_Range_Index
203. First_Order_Histogram::Bins-802_FullImage::Range_Index
204. First_Order_Histogram::Bins-802_FullImage::Mean_Absolute_Deviation_Index
205. First_Order_Histogram::Bins-802_FullImage::Robust_Mean_Absolute_Deviation_Index
206. First_Order_Histogram::Bins-802_FullImage::Median_Absolute_Deviation_Index
207. First_Order_Histogram::Bins-802_FullImage::Coefficient_of_Variation_Index
208. First_Order_Histogram::Bins-802_FullImage::Quantile_coefficient_of_Dispersion_Index
209. First_Order_Histogram::Bins-802_FullImage::Entropy_Index
210. First_Order_Histogram::Bins-802_FullImage::Uniformity_Index
211. First_Order_Histogram::Bins-802_FullImage::Maximum_Gradient
212. First_Order_Histogram::Bins-802_FullImage::Maximum_Gradient_Index
213. First_Order_Histogram::Bins-802_FullImage::Minimum_Gradient
214. First_Order_Histogram::Bins-802_FullImage::Minimum_Gradient_Index
215. First_Order_Histogram::Bins-802_FullImage::Robust_Mean_Index
216. First_Order_Histogram::Bins-802_FullImage::Number_of_Bins
217. First_Order_Histogram::Bins-802_FullImage::Bin_Size
218. Intensity_Volume_Histogram::Bins-1000_FullImage::Volume_fraction_at_0.10_intensity
219. Intensity_Volume_Histogram::Bins-1000_FullImage::Volume_fraction_at_0.90_intensity
220. Intensity_Volume_Histogram::Bins-1000_FullImage::Intensity_at_0.10_volume
221. Intensity_Volume_Histogram::Bins-1000_FullImage::Intensity_at_0.90_volume
222. Intensity_Volume_Histogram::Bins-1000_FullImage::Difference_volume_fraction_at_0.10_and_0.90_intensity

223. Intensity_Volume_Histogram::Bins-1000_FullImage::Difference_intensity_at_0.10_and_0.90_volume
224. Intensity_Volume_Histogram::Bins-1000_FullImage::Area_under_IVH_curve
225. Local_Intensity::Range-6.2::Local_Intensity_Peak
226. Local_Intensity::Range-6.2::Global_Intensity_Peak
227. Co-occurenced_Based_Features::Bins-128_FullImage_Range-1::Overall_Joint_Maximum
228. Co-occurenced_Based_Features::Bins-128_FullImage_Range-1::Overall_Joint_Average
229. Co-occurenced_Based_Features::Bins-128_FullImage_Range-1::Overall_Joint_Variance
230. Co-occurenced_Based_Features::Bins-128_FullImage_Range-1::Overall_Joint_Entropy
231. Co-occurenced_Based_Features::Bins-128_FullImage_Range-1::Overall_Difference_Average
232. Co-occurenced_Based_Features::Bins-128_FullImage_Range-1::Overall_Difference_Variance
233. Co-occurenced_Based_Features::Bins-128_FullImage_Range-1::Overall_Difference_Entropy
234. Co-occurenced_Based_Features::Bins-128_FullImage_Range-1::Overall_Sum_Average
235. Co-occurenced_Based_Features::Bins-128_FullImage_Range-1::Overall_Sum_Variance
236. Co-occurenced_Based_Features::Bins-128_FullImage_Range-1::Overall_Sum_Entropy
237. Co-occurenced_Based_Features::Bins-128_FullImage_Range-1::Overall_Angular_Second_Moment
238. Co-occurenced_Based_Features::Bins-128_FullImage_Range-1::Overall_Contrast
239. Co-occurenced_Based_Features::Bins-128_FullImage_Range-1::Overall_Dissimilarity
240. Co-occurenced_Based_Features::Bins-128_FullImage_Range-1::Overall_Inverse_Difference
241. Co-occurenced_Based_Features::Bins-128_FullImage_Range-1::Overall_Inverse_Difference_Normalized
242. Co-occurenced_Based_Features::Bins-128_FullImage_Range-1::Overall_Inverse_Difference_Moment
243. Co-occurenced_Based_Features::Bins-128_FullImage_Range-1::Overall_Inverse_Difference_Moment_Normalized
244. Co-occurenced_Based_Features::Bins-128_FullImage_Range-1::Overall_Inverse_Variance
245. Co-occurenced_Based_Features::Bins-128_FullImage_Range-1::Overall_Correlation
246. Co-occurenced_Based_Features::Bins-128_FullImage_Range-1::Overall_Autocorrelation
247. Co-occurenced_Based_Features::Bins-128_FullImage_Range-1::Overall_Cluster_Tendency
248. Co-occurenced_Based_Features::Bins-128_FullImage_Range-1::Overall_Cluster_Shade
249. Co-occurenced_Based_Features::Bins-128_FullImage_Range-1::Overall_Cluster_Prominence
250. Co-occurenced_Based_Features::Bins-128_FullImage_Range-1::Overall_First_Measure_of_Information_Correlation
251. Co-occurenced_Based_Features::Bins-128_FullImage_Range-1::Overall_Second_Measure_of_Information_Correlation
252. Co-occurenced_Based_Features::Bins-128_FullImage_Range-1::Overall_Row_Maximum
253. Co-occurenced_Based_Features::Bins-128_FullImage_Range-1::Overall_Row_Average
254. Co-occurenced_Based_Features::Bins-128_FullImage_Range-1::Overall_Row_Variance
255. Co-occurenced_Based_Features::Bins-128_FullImage_Range-1::Overall_Row_Entropy
256. Co-occurenced_Based_Features::Bins-128_FullImage_Range-1::Overall_First_Row-Column_Entropy
257. Co-occurenced_Based_Features::Bins-128_FullImage_Range-1::Overall_Second_Row-Column_Entropy
258. Co-occurenced_Based_Features::Bins-128_FullImage_Range-1::Mean_Joint_Maximum
259. Co-occurenced_Based_Features::Bins-128_FullImage_Range-1::Mean_Joint_Average
260. Co-occurenced_Based_Features::Bins-128_FullImage_Range-1::Mean_Joint_Variance
261. Co-occurenced_Based_Features::Bins-128_FullImage_Range-1::Mean_Joint_Entropy
262. Co-occurenced_Based_Features::Bins-128_FullImage_Range-1::Mean_Difference_Average
263. Co-occurenced_Based_Features::Bins-128_FullImage_Range-1::Mean_Difference_Variance
264. Co-occurenced_Based_Features::Bins-128_FullImage_Range-1::Mean_Difference_Entropy
265. Co-occurenced_Based_Features::Bins-128_FullImage_Range-1::Mean_Sum_Average
266. Co-occurenced_Based_Features::Bins-128_FullImage_Range-1::Mean_Sum_Variance
267. Co-occurenced_Based_Features::Bins-128_FullImage_Range-1::Mean_Sum_Entropy
268. Co-occurenced_Based_Features::Bins-128_FullImage_Range-1::Mean_Angular_Second_Moment
269. Co-occurenced_Based_Features::Bins-128_FullImage_Range-1::Mean_Contrast
270. Co-occurenced_Based_Features::Bins-128_FullImage_Range-1::Mean_Dissimilarity
271. Co-occurenced_Based_Features::Bins-128_FullImage_Range-1::Mean_Inverse_Difference
272. Co-occurenced_Based_Features::Bins-128_FullImage_Range-1::Mean_Inverse_Difference_Normalized
273. Co-occurenced_Based_Features::Bins-128_FullImage_Range-1::Mean_Inverse_Difference_Moment
274. Co-occurenced_Based_Features::Bins-128_FullImage_Range-1::Mean_Inverse_Difference_Moment_Normalized

275. Co-occurenced_Based_Features::Bins-128_FullImage_Range-1::Mean_Inverse_Variance
276. Co-occurenced_Based_Features::Bins-128_FullImage_Range-1::Mean_Autocorrelation
277. Co-occurenced_Based_Features::Bins-128_FullImage_Range-1::Mean_Cluster_Tendency
278. Co-occurenced_Based_Features::Bins-128_FullImage_Range-1::Mean_Cluster_Shade
279. Co-occurenced_Based_Features::Bins-128_FullImage_Range-1::Mean_Cluster_Prominence
280. Co-occurenced_Based_Features::Bins-128_FullImage_Range-1::Mean_Second_Measure_of_Information_Correlation
281. Co-occurenced_Based_Features::Bins-128_FullImage_Range-1::Mean_Row_Maximum
282. Co-occurenced_Based_Features::Bins-128_FullImage_Range-1::Mean_Row_Average
283. Co-occurenced_Based_Features::Bins-128_FullImage_Range-1::Mean_Row_Variance
284. Co-occurenced_Based_Features::Bins-128_FullImage_Range-1::Mean_Row_Entropy
285. Co-occurenced_Based_Features::Bins-128_FullImage_Range-1::Mean_First_Row-Column_Entropy
286. Co-occurenced_Based_Features::Bins-128_FullImage_Range-1::Mean_Second_Row-Column_Entropy
287. Co-occurenced_Based_Features::Bins-128_FullImage_Range-1::Std.Dev._Joint_Maximum
288. Co-occurenced_Based_Features::Bins-128_FullImage_Range-1::Std.Dev._Joint_Average
289. Co-occurenced_Based_Features::Bins-128_FullImage_Range-1::Std.Dev._Joint_Variance
290. Co-occurenced_Based_Features::Bins-128_FullImage_Range-1::Std.Dev._Joint_Entropy
291. Co-occurenced_Based_Features::Bins-128_FullImage_Range-1::Std.Dev._Difference_Average
292. Co-occurenced_Based_Features::Bins-128_FullImage_Range-1::Std.Dev._Difference_Variance
293. Co-occurenced_Based_Features::Bins-128_FullImage_Range-1::Std.Dev._Difference_Entropy
294. Co-occurenced_Based_Features::Bins-128_FullImage_Range-1::Std.Dev._Sum_Average
295. Co-occurenced_Based_Features::Bins-128_FullImage_Range-1::Std.Dev._Sum_Variance
296. Co-occurenced_Based_Features::Bins-128_FullImage_Range-1::Std.Dev._Sum_Entropy
297. Co-occurenced_Based_Features::Bins-128_FullImage_Range-1::Std.Dev._Angular_Second_Moment
298. Co-occurenced_Based_Features::Bins-128_FullImage_Range-1::Std.Dev._Contrast
299. Co-occurenced_Based_Features::Bins-128_FullImage_Range-1::Std.Dev._Dissimilarity
300. Co-occurenced_Based_Features::Bins-128_FullImage_Range-1::Std.Dev._Inverse_Difference
301. Co-occurenced_Based_Features::Bins-128_FullImage_Range-1::Std.Dev._Inverse_Difference_Normalized
302. Co-occurenced_Based_Features::Bins-128_FullImage_Range-1::Std.Dev._Inverse_Difference_Moment
303. Co-occurenced_Based_Features::Bins-128_FullImage_Range-1::Std.Dev._Inverse_Difference_Moment_Normalized
304. Co-occurenced_Based_Features::Bins-128_FullImage_Range-1::Std.Dev._Inverse_Variance
305. Co-occurenced_Based_Features::Bins-128_FullImage_Range-1::Std.Dev._Autocorrelation
306. Co-occurenced_Based_Features::Bins-128_FullImage_Range-1::Std.Dev._Cluster_Tendency
307. Co-occurenced_Based_Features::Bins-128_FullImage_Range-1::Std.Dev._Cluster_Shade
308. Co-occurenced_Based_Features::Bins-128_FullImage_Range-1::Std.Dev._Cluster_Prominence
309. Co-occurenced_Based_Features::Bins-128_FullImage_Range-1::Std.Dev._Second_Measure_of_Information_Correlation
310. Co-occurenced_Based_Features::Bins-128_FullImage_Range-1::Std.Dev._Row_Maximum
311. Co-occurenced_Based_Features::Bins-128_FullImage_Range-1::Std.Dev._Row_Average
312. Co-occurenced_Based_Features::Bins-128_FullImage_Range-1::Std.Dev._Row_Variance
313. Co-occurenced_Based_Features::Bins-128_FullImage_Range-1::Std.Dev._Row_Entropy
314. Co-occurenced_Based_Features::Bins-128_FullImage_Range-1::Std.Dev._First_Row-Column_Entropy
315. Co-occurenced_Based_Features::Bins-128_FullImage_Range-1::Std.Dev._Second_Row-Column_Entropy
316. Neighbouring_Grey_Level_Dependence::Bins-128_FullImage_Range-1::Low_Dependence_Emphasis
317. Neighbouring_Grey_Level_Dependence::Bins-128_FullImage_Range-1::High_Dependence_Emphasis
318. Neighbouring_Grey_Level_Dependence::Bins-128_FullImage_Range-1::Low_Grey_Level_Count_Emphasis
319. Neighbouring_Grey_Level_Dependence::Bins-128_FullImage_Range-1::High_Grey_Level_Count_Emphasis
320. Neighbouring_Grey_Level_Dependence::Bins-128_FullImage_Range-1::Low_Dependence_Low_Grey_Level_Emphasis
321. Neighbouring_Grey_Level_Dependence::Bins-128_FullImage_Range-1::Low_Dependence_High_Grey_Level_Emphasis
322. Neighbouring_Grey_Level_Dependence::Bins-128_FullImage_Range-1::High_Dependence_Low_Grey_Level_Emphasis
323. Neighbouring_Grey_Level_Dependence::Bins-128_FullImage_Range-1::High_Dependence_High_Grey_Level_Emphasis

324. Neighbouring_Grey_Level_Dependence::Bins-128_FullImage_Range-1::Grey_Level_Non-Uniformity
325. Neighbouring_Grey_Level_Dependence::Bins-128_FullImage_Range-1::Grey_Level_Non-Uniformity_Normalised
326. Neighbouring_Grey_Level_Dependence::Bins-128_FullImage_Range-1::Dependence_Count_Non-Uniformity
327. Neighbouring_Grey_Level_Dependence::Bins-128_FullImage_Range-1::Dependence_Count_Non-Uniformity_Normalised
328. Neighbouring_Grey_Level_Dependence::Bins-128_FullImage_Range-1::Dependence_Count_Percentage
329. Neighbouring_Grey_Level_Dependence::Bins-128_FullImage_Range-1::Grey_Level_Mean
330. Neighbouring_Grey_Level_Dependence::Bins-128_FullImage_Range-1::Grey_Level_Variance
331. Neighbouring_Grey_Level_Dependence::Bins-128_FullImage_Range-1::Dependence_Count_Mean
332. Neighbouring_Grey_Level_Dependence::Bins-128_FullImage_Range-1::Dependence_Count_Variance
333. Neighbouring_Grey_Level_Dependence::Bins-128_FullImage_Range-1::Dependence_Count_Entropy
334. Neighbouring_Grey_Level_Dependence::Bins-128_FullImage_Range-1::Dependence_Count_Energy
335. Neighbouring_Grey_Level_Dependence::Bins-128_FullImage_Range-1::Expected_Neighbourhood_Size
336. Neighbouring_Grey_Level_Dependence::Bins-128_FullImage_Range-1::Average_Neighbourhood_Size
337. Neighbouring_Grey_Level_Dependence::Bins-128_FullImage_Range-1::Average_Incomplete_Neighbourhood_Size
338. Neighbouring_Grey_Level_Dependence::Bins-128_FullImage_Range-1::Percentage_of_complete_Neighbourhoods
339. Neighbouring_Grey_Level_Dependence::Bins-128_FullImage_Range-1::Percentage_of_Dependence_Neighbour_Voxels
340. Run_Length::Bins-128_FullImage::Short_run_emphasis_Means
341. Run_Length::Bins-128_FullImage::Short_run_emphasis_Std.
342. Run_Length::Bins-128_FullImage::Short_run_emphasis_Comb.
343. Run_Length::Bins-128_FullImage::Long_run_emphasis_Means
344. Run_Length::Bins-128_FullImage::Long_run_emphasis_Std.
345. Run_Length::Bins-128_FullImage::Long_run_emphasis_Comb.
346. Run_Length::Bins-128_FullImage::Grey_level_nonuniformity_Means
347. Run_Length::Bins-128_FullImage::Grey_level_nonuniformity_Std.
348. Run_Length::Bins-128_FullImage::Grey_level_nonuniformity_Comb.
349. Run_Length::Bins-128_FullImage::Grey_level_nonuniformity_normalized_Means
350. Run_Length::Bins-128_FullImage::Grey_level_nonuniformity_normalized_Std.
351. Run_Length::Bins-128_FullImage::Grey_level_nonuniformity_normalized_Comb.
352. Run_Length::Bins-128_FullImage::Run_length_nonuniformity_Means
353. Run_Length::Bins-128_FullImage::Run_length_nonuniformity_Std.
354. Run_Length::Bins-128_FullImage::Run_length_nonuniformity_Comb.
355. Run_Length::Bins-128_FullImage::Run_length_nonuniformity_normalized_Means
356. Run_Length::Bins-128_FullImage::Run_length_nonuniformity_normalized_Std.
357. Run_Length::Bins-128_FullImage::Run_length_nonuniformity_normalized_Comb.
358. Run_Length::Bins-128_FullImage::Low_grey_level_run_emphasis_Means
359. Run_Length::Bins-128_FullImage::Low_grey_level_run_emphasis_Std.
360. Run_Length::Bins-128_FullImage::Low_grey_level_run_emphasis_Comb.
361. Run_Length::Bins-128_FullImage::High_grey_level_run_emphasis_Means
362. Run_Length::Bins-128_FullImage::High_grey_level_run_emphasis_Std.
363. Run_Length::Bins-128_FullImage::High_grey_level_run_emphasis_Comb.
364. Run_Length::Bins-128_FullImage::Short_run_low_grey_level_emphasis_Means
365. Run_Length::Bins-128_FullImage::Short_run_low_grey_level_emphasis__Std.
366. Run_Length::Bins-128_FullImage::Short_run_low_grey_level_emphasis__Comb.
367. Run_Length::Bins-128_FullImage::Short_run_high_grey_level_emphasis_Means
368. Run_Length::Bins-128_FullImage::Short_run_high_grey_level_emphasis_Std.
369. Run_Length::Bins-128_FullImage::Short_run_high_grey_level_emphasis_Comb.
370. Run_Length::Bins-128_FullImage::Long_run_low_grey_level_emphasis_Means
371. Run_Length::Bins-128_FullImage::Long_run_low_grey_level_emphasis_Std.
372. Run_Length::Bins-128_FullImage::Long_run_low_grey_level_emphasis_Comb.
373. Run_Length::Bins-128_FullImage::Long_run_high_grey_level_emphasis_Means
374. Run_Length::Bins-128_FullImage::Long_run_high_grey_level_emphasis_Std.
375. Run_Length::Bins-128_FullImage::Long_run_high_grey_level_emphasis_Comb.

376. Run_Length::Bins-128_FullImage::Run_percentage_Means
377. Run_Length::Bins-128_FullImage::Run_percentage_Std.
378. Run_Length::Bins-128_FullImage::Run_percentage_Comb.
379. Run_Length::Bins-128_FullImage::Number_of_runs_Means
380. Run_Length::Bins-128_FullImage::Number_of_runs_Std.
381. Run_Length::Bins-128_FullImage::Number_of_runs_Comb.
382. Run_Length::Bins-128_FullImage::Grey_level_variance_Means
383. Run_Length::Bins-128_FullImage::Grey_level_variance_Std.
384. Run_Length::Bins-128_FullImage::Grey_level_variance_Comb.
385. Run_Length::Bins-128_FullImage::Run_length_variance_Means
386. Run_Length::Bins-128_FullImage::Run_length_variance_Std.
387. Run_Length::Bins-128_FullImage::Run_length_variance_Comb.
388. Run_Length::Bins-128_FullImage::Run_length_entropy_Means
389. Run_Length::Bins-128_FullImage::Run_length_entropy_Std.
390. Run_Length::Bins-128_FullImage::Run_length_entropy_Comb.
391. Grey_Level_Size_Zone::Bins-128_FullImage::Small_Zone_Emphasis
392. Grey_Level_Size_Zone::Bins-128_FullImage::Large_Zone_Emphasis
393. Grey_Level_Size_Zone::Bins-128_FullImage::Low_Grey_Level_Emphasis
394. Grey_Level_Size_Zone::Bins-128_FullImage::High_Grey_Level_Emphasis
395. Grey_Level_Size_Zone::Bins-128_FullImage::Small_Zone_Low_Grey_Level_Emphasis
396. Grey_Level_Size_Zone::Bins-128_FullImage::Small_Zone_High_Grey_Level_Emphasis
397. Grey_Level_Size_Zone::Bins-128_FullImage::Large_Zone_Low_Grey_Level_Emphasis
398. Grey_Level_Size_Zone::Bins-128_FullImage::Large_Zone_High_Grey_Level_Emphasis
399. Grey_Level_Size_Zone::Bins-128_FullImage::Grey_Level_Non-Uniformity
400. Grey_Level_Size_Zone::Bins-128_FullImage::Grey_Level_Non-Uniformity_Normalized
401. Grey_Level_Size_Zone::Bins-128_FullImage::Zone_Size_Non-Uniformity
402. Grey_Level_Size_Zone::Bins-128_FullImage::Zone_Size_Non-Uniformity_Normalized
403. Grey_Level_Size_Zone::Bins-128_FullImage::Zone_Percentage
404. Grey_Level_Size_Zone::Bins-128_FullImage::Grey_Level_Mean
405. Grey_Level_Size_Zone::Bins-128_FullImage::Grey_Level_Variance
406. Grey_Level_Size_Zone::Bins-128_FullImage::Zone_Size_Mean
407. Grey_Level_Size_Zone::Bins-128_FullImage::Zone_Size_Variance
408. Grey_Level_Size_Zone::Bins-128_FullImage::Zone_Size_Entropy
409. Diagnostic::::Image_Dimension_X
410. Diagnostic::::Image_Dimension_Y
411. Diagnostic::::Image_Dimension_Z
412. Diagnostic::::Image_Spacing_X
413. Diagnostic::::Image_Spacing_Y
414. Diagnostic::::Image_Spacing_Z
415. Diagnostic::::Image_Mean_intensity
416. Diagnostic::::Image_Minimum_intensity
417. Diagnostic::::Image_Maximum_intensity
418. Diagnostic::::Mask_Dimension_X
419. Diagnostic::::Mask_Dimension_Y
420. Diagnostic::::Mask_Dimension_Z
421. Diagnostic::::Mask_bounding_box_X
422. Diagnostic::::Mask_bounding_box_Y
423. Diagnostic::::Mask_bounding_box_Z
424. Diagnostic::::Mask_Spacing_X
425. Diagnostic::::Mask_Spacing_Y
426. Diagnostic::::Mask_Spacing_Z
427. Diagnostic::::Mask_Voxel_Count
428. Diagnostic::::Mask_Mean_intensity
429. Diagnostic::::Mask_Minimum_intensity
430. Diagnostic::::Mask_Maximum_intensity
431. Neighbourhood_Grey_Tone_Difference::Bins-128_FullImage_Range-1::Coarsness

432. Neighbourhood_Grey_Tone_Difference::Bins-128_FullImage_Range-1::Contrast
433. Neighbourhood_Grey_Tone_Difference::Bins-128_FullImage_Range-1::Busyness
434. Neighbourhood_Grey_Tone_Difference::Bins-128_FullImage_Range-1::Complexity
435. Neighbourhood_Grey_Tone_Difference::Bins-128_FullImage_Range-1::Strength

A8 Feature Noise Distributions for Annotator

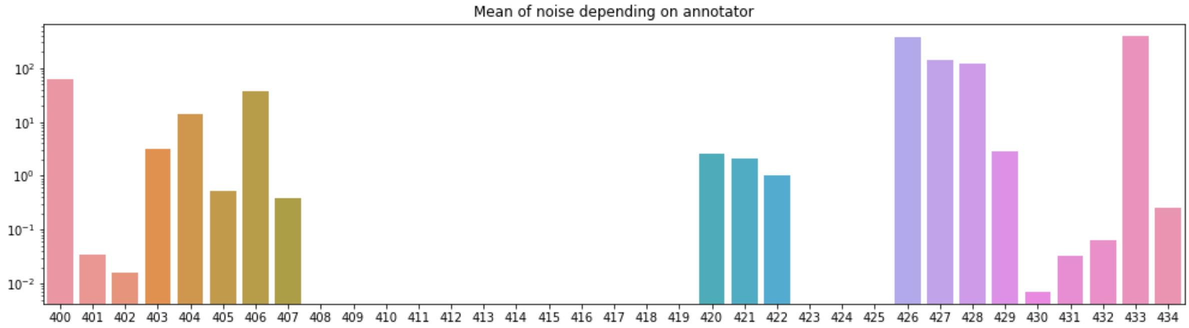

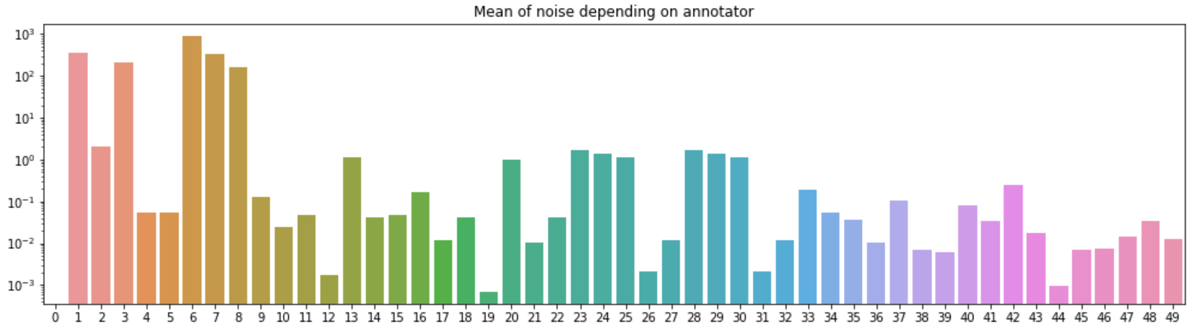

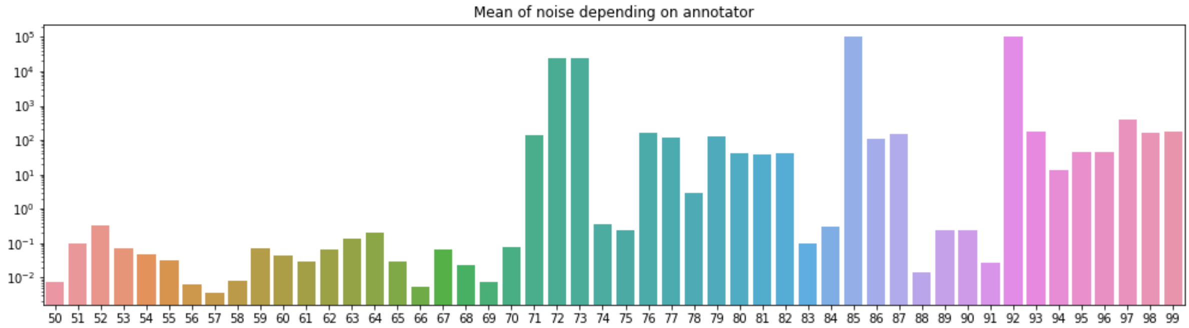

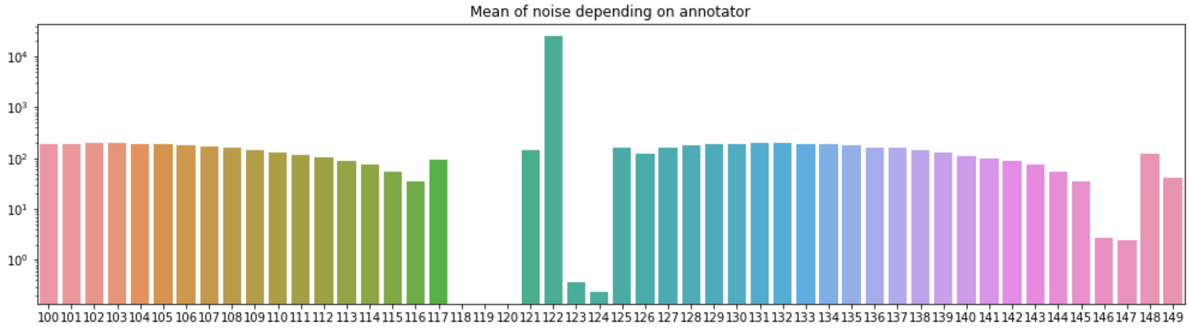

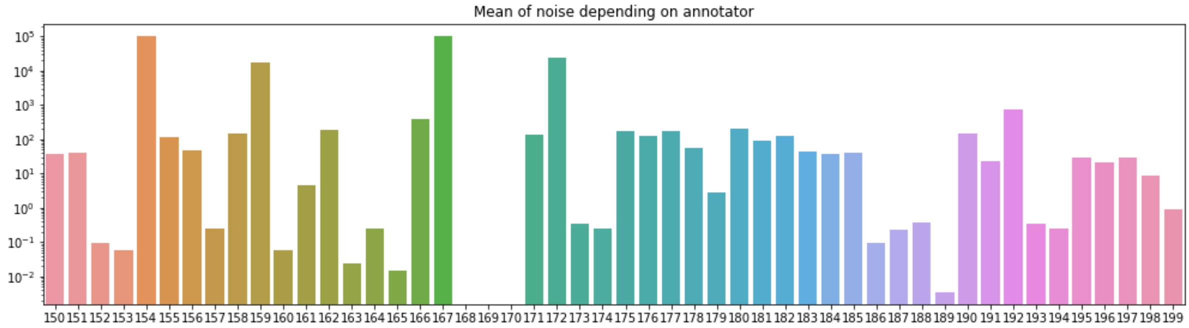
Mean of noise depending on annotator

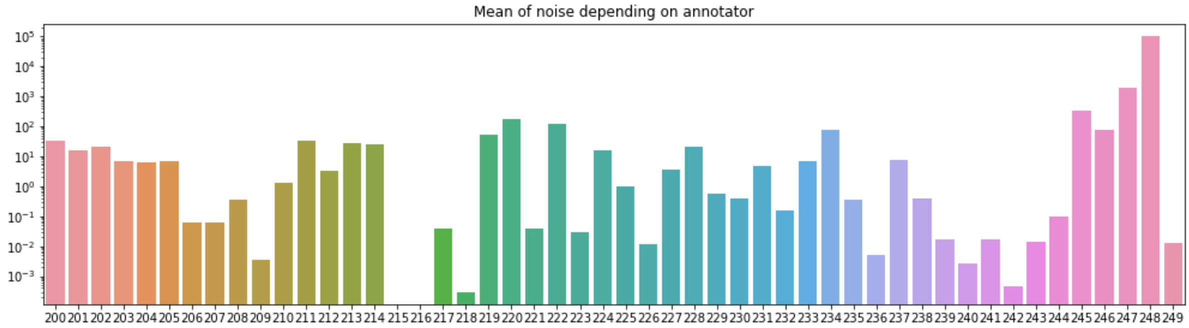
Mean of noise depending on annotator

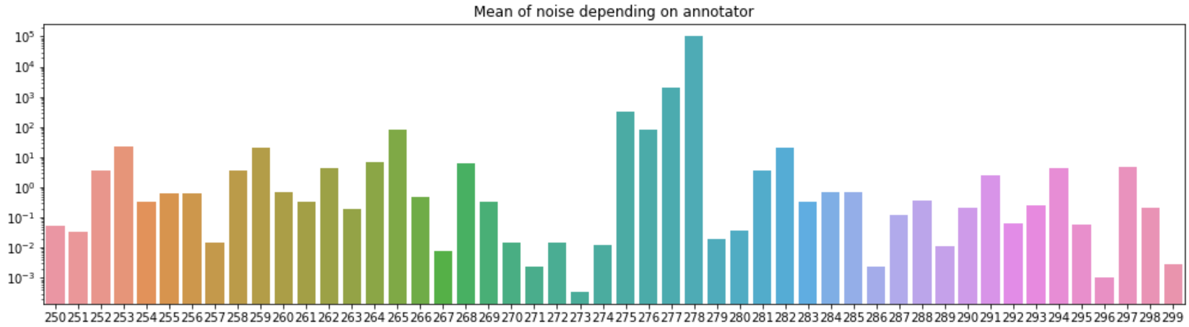
Mean of noise depending on annotator

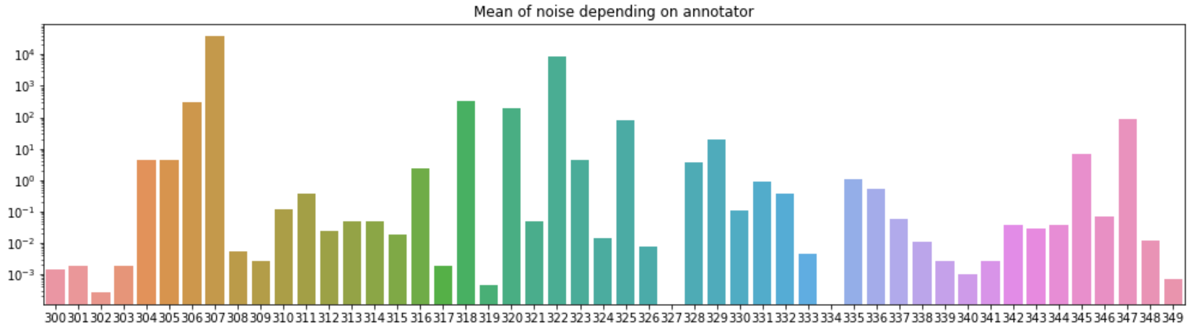
Mean of noise depending on annotator

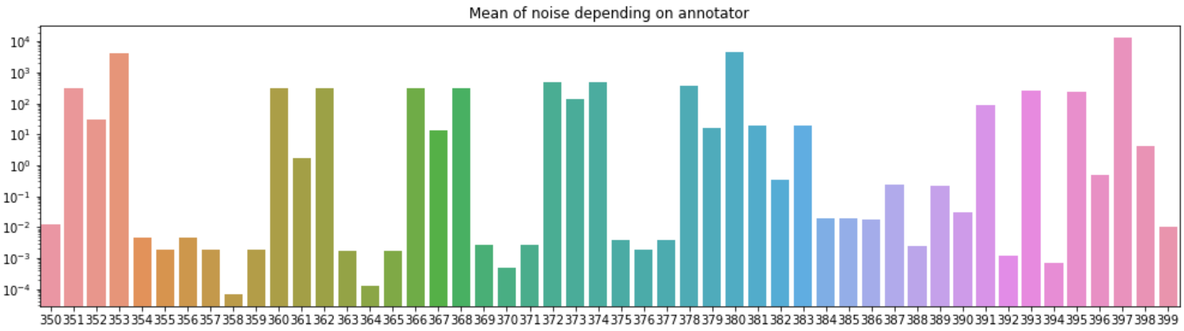
Mean of noise depending on annotator

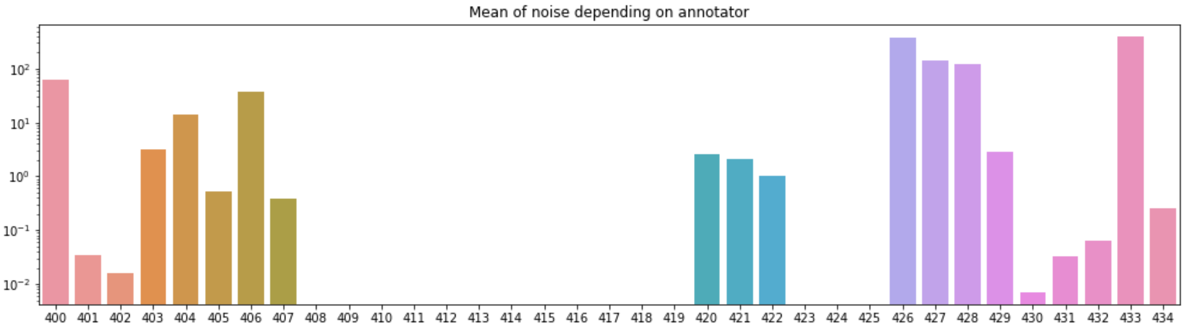
Mean of noise depending on annotator

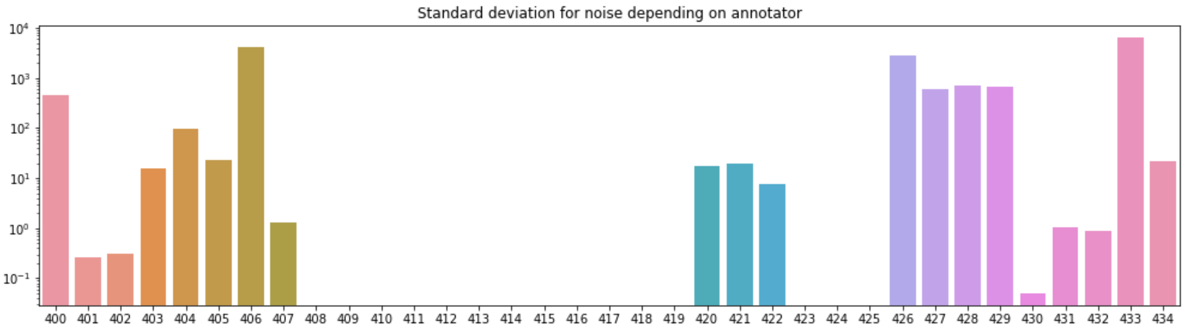
Standard deviation for noise depending on annotator

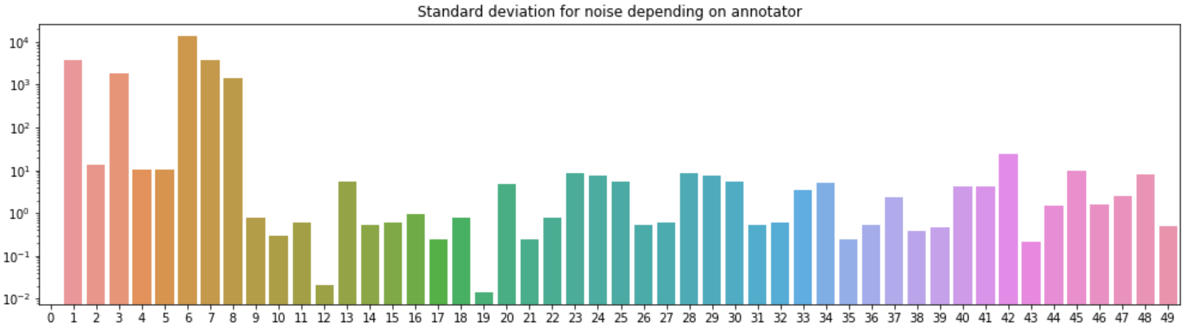
Standard deviation for noise depending on annotator

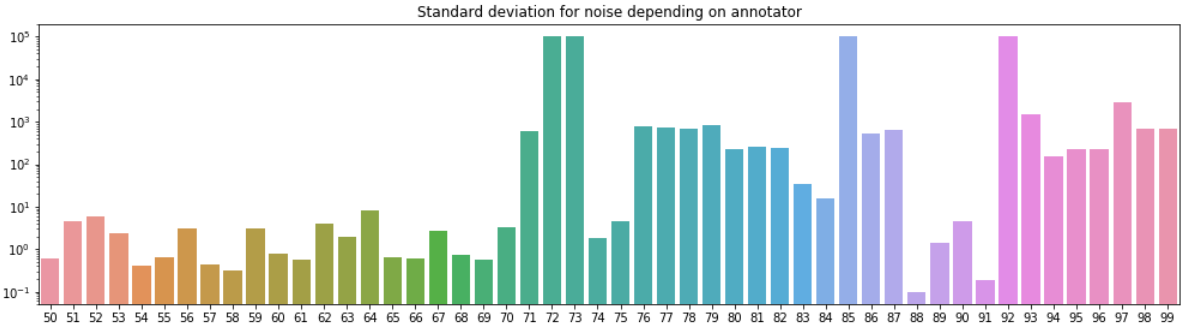
Standard deviation for noise depending on annotator

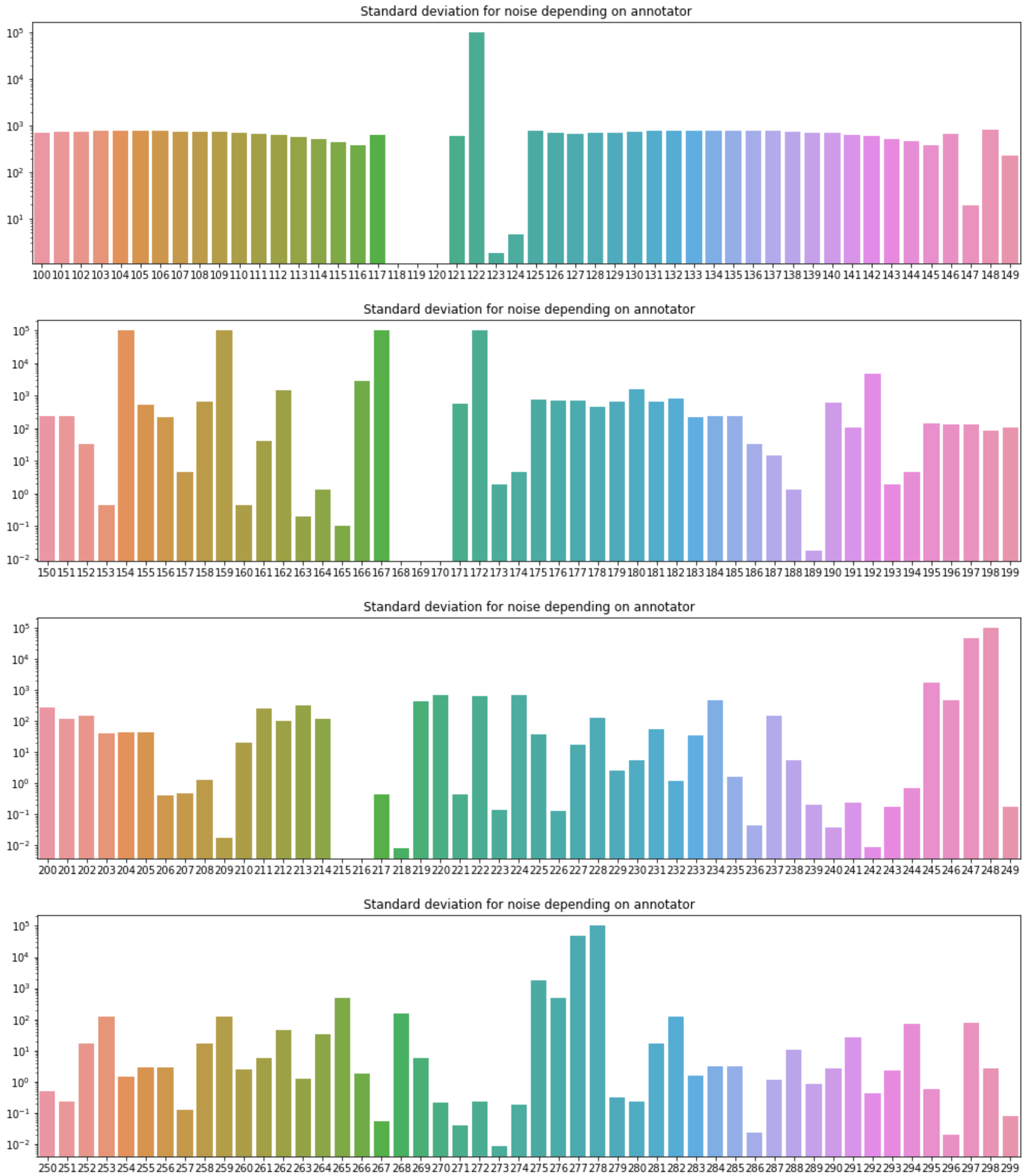

Standard deviation for noise depending on annotator
100 101 102 103 104 105 106 107 108 109 110 111 112 113 114 115 116 117 118 119 120 121 122 123 124 125 126 127 128 129 130 131 132 133 134 135 136 137 138 139 140 141 142 143 144 145 146 147 148 149
Standard deviation for noise depending on annotator
150 151 152 153 154 155 156 157 158 159 160 161 162 163 164 165 166 167 168 169 170 171 172 173 174 175 176 177 178 179 180 181 182 183 184 185 186 187 188 189 190 191 192 193 194 195 196 197 198 199
Standard deviation for noise depending on annotator
200 201 202 203 204 205 206 207 208 209 210 211 212 213 214 215 216 217 218 219 220 221 222 223 224 225 226 227 228 229 230 231 232 233 234 235 236 237 238 239 240 241 242 243 244 245 246 247 248 249
Standard deviation for noise depending on annotator
250 251 252 253 254 255 256 257 258 259 260 261 262 263 264 265 266 267 268 269 270 271 272 273 274 275 276 277 278 279 280 281 282 283 284 285 286 287 288 289 290 291 292 293 294 295 296 297 298 299

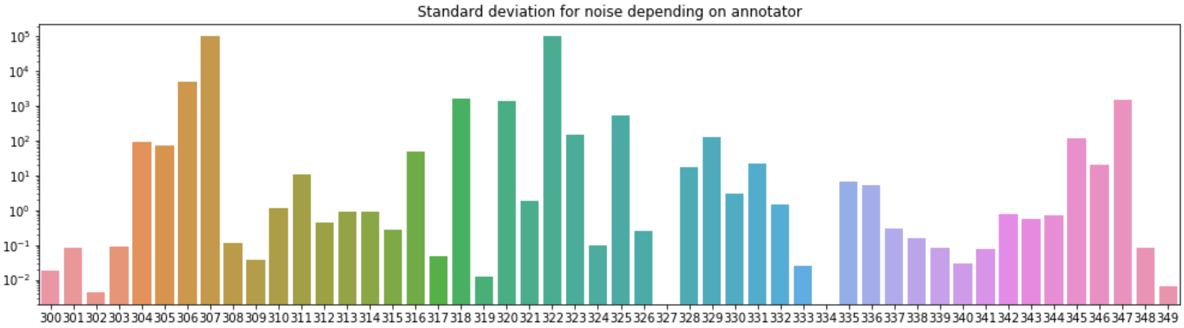

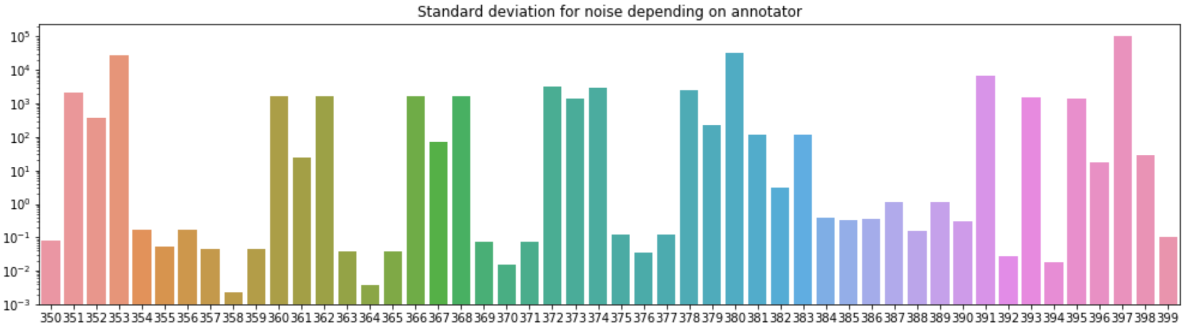

A9 Feature Noise Distributions for Manufacturer

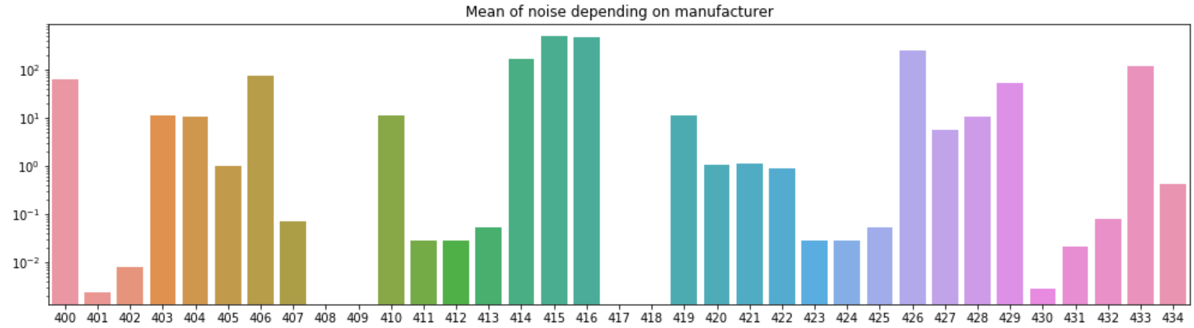

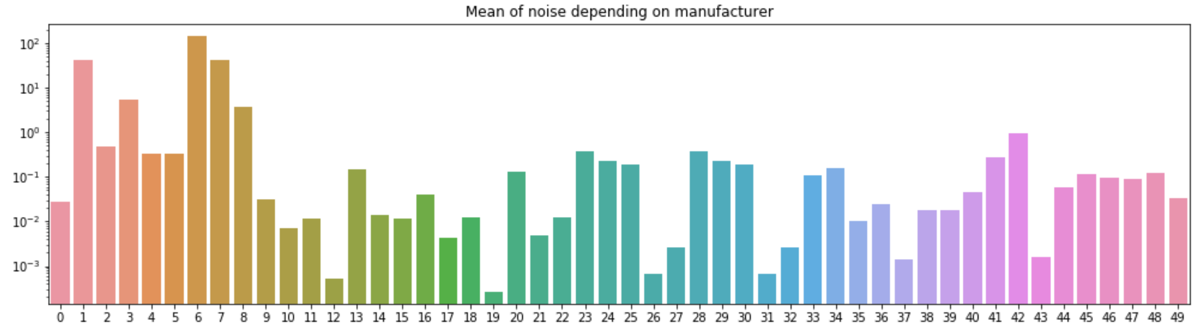

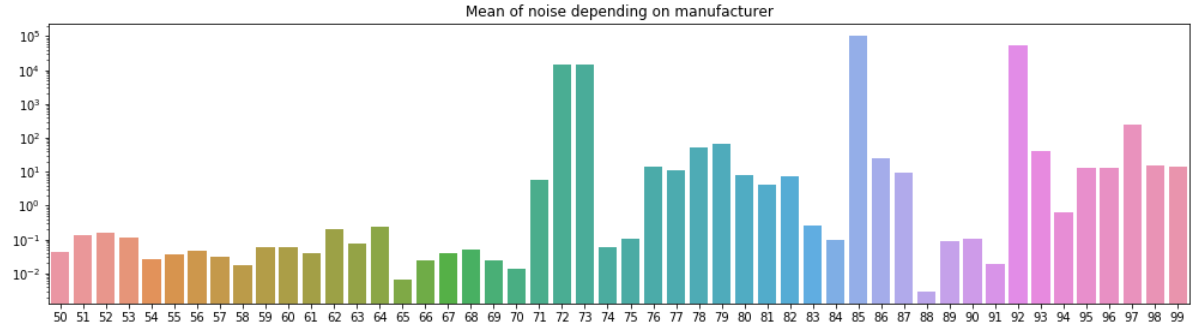

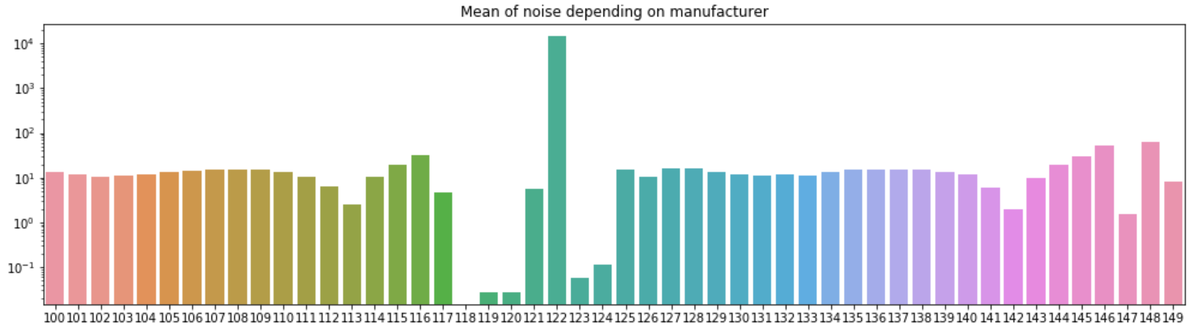
Mean of noise depending on manufacturer
10^4
10^3
10^2
10^1
10^0
10^{-1}
100 101 102 103 104 105 106 107 108 109 110 111 112 113 114 115 116 117 118 119 120 121 122 123 124 125 126 127 128 129 130 131 132 133 134 135 136 137 138 139 140 141 142 143 144 145 146 147 148 149

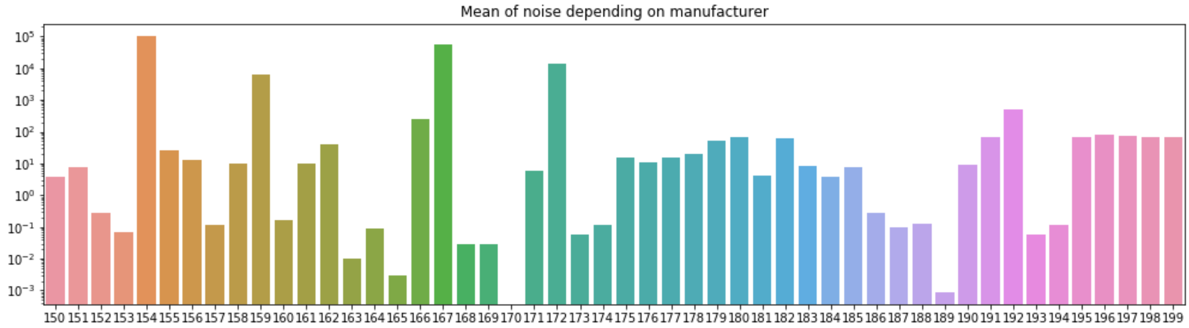
Mean of noise depending on manufacturer
10^5
10^4
10^3
10^2
10^1
10^0
10^{-1}
10^{-2}
10^{-3}
150 151 152 153 154 155 156 157 158 159 160 161 162 163 164 165 166 167 168 169 170 171 172 173 174 175 176 177 178 179 180 181 182 183 184 185 186 187 188 189 190 191 192 193 194 195 196 197 198 199

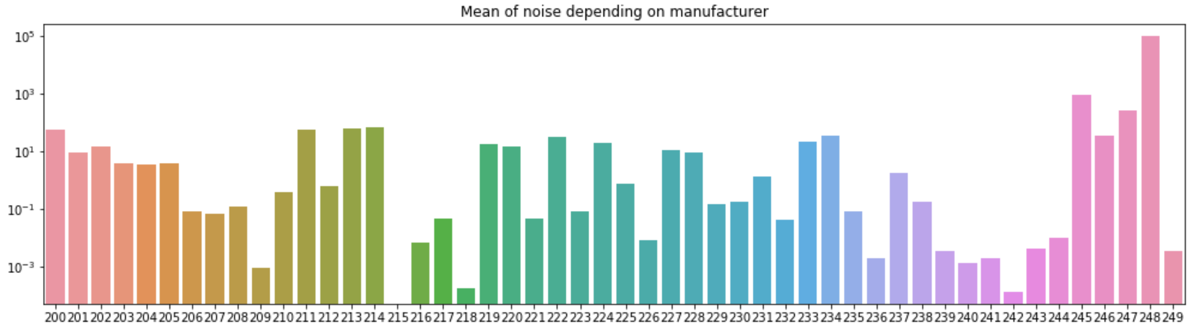
Mean of noise depending on manufacturer
10^5
10^3
10^1
10^{-1}
10^{-3}
200 201 202 203 204 205 206 207 208 209 210 211 212 213 214 215 216 217 218 219 220 221 222 223 224 225 226 227 228 229 230 231 232 233 234 235 236 237 238 239 240 241 242 243 244 245 246 247 248 249

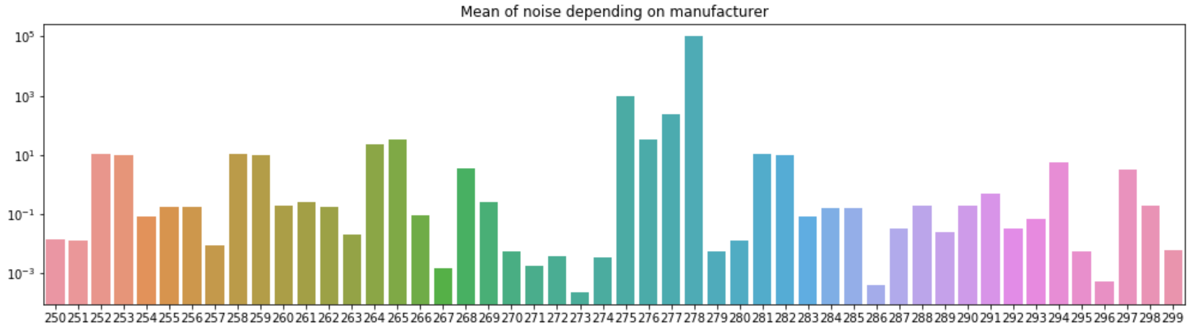
Mean of noise depending on manufacturer
10^5
10^3
10^1
10^{-1}
10^{-3}
250 251 252 253 254 255 256 257 258 259 260 261 262 263 264 265 266 267 268 269 270 271 272 273 274 275 276 277 278 279 280 281 282 283 284 285 286 287 288 289 290 291 292 293 294 295 296 297 298 299

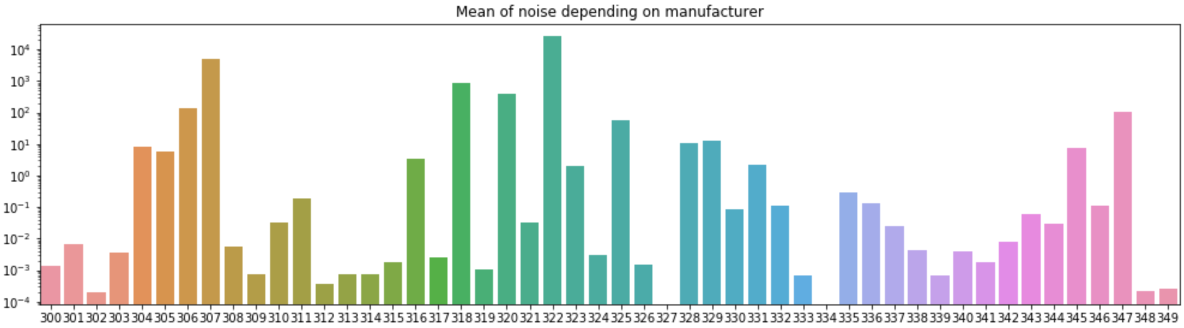
Mean of noise depending on manufacturer
10^{-4} 10^{-3} 10^{-2} 10^{-1} 10^{0} 10^{1} 10^{2} 10^{3} 10^{4}
300 301 302 303 304 305 306 307 308 309 310 311 312 313 314 315 316 317 318 319 320 321 322 323 324 325 326 327 328 329 330 331 332 333 334 335 336 337 338 339 340 341 342 343 344 345 346 347 348 349

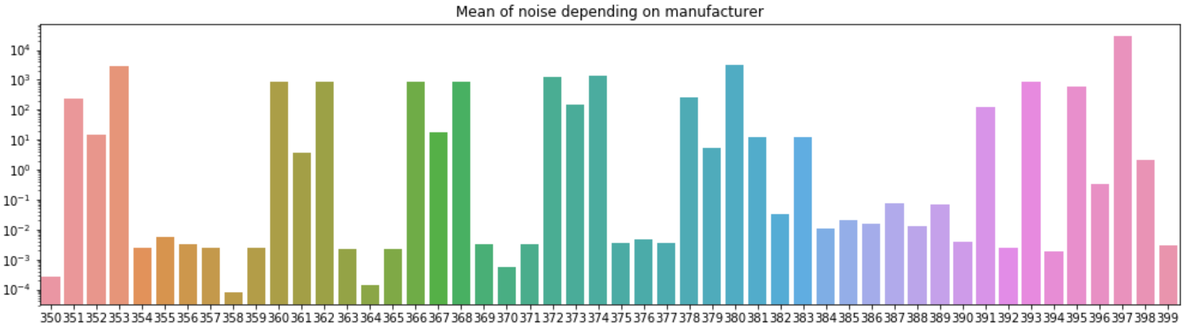
Mean of noise depending on manufacturer
10^{-4} 10^{-3} 10^{-2} 10^{-1} 10^{0} 10^{1} 10^{2} 10^{3} 10^{4}
350 351 352 353 354 355 356 357 358 359 360 361 362 363 364 365 366 367 368 369 370 371 372 373 374 375 376 377 378 379 380 381 382 383 384 385 386 387 388 389 390 391 392 393 394 395 396 397 398 399

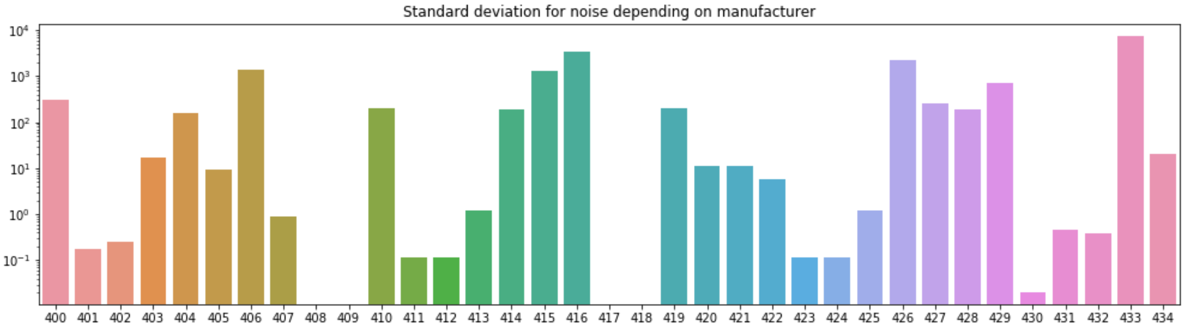
Standard deviation for noise depending on manufacturer
10^{-1} 10^{0} 10^{1} 10^{2} 10^{3} 10^{4}
400 401 402 403 404 405 406 407 408 409 410 411 412 413 414 415 416 417 418 419 420 421 422 423 424 425 426 427 428 429 430 431 432 433 434

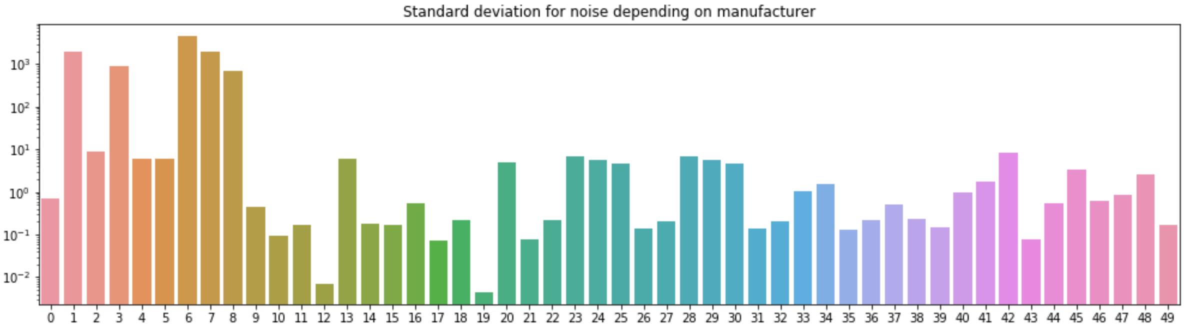
Standard deviation for noise depending on manufacturer
10^{-2} 10^{-1} 10^{0} 10^{1} 10^{2} 10^{3}
0 1 2 3 4 5 6 7 8 9 10 11 12 13 14 15 16 17 18 19 20 21 22 23 24 25 26 27 28 29 30 31 32 33 34 35 36 37 38 39 40 41 42 43 44 45 46 47 48 49

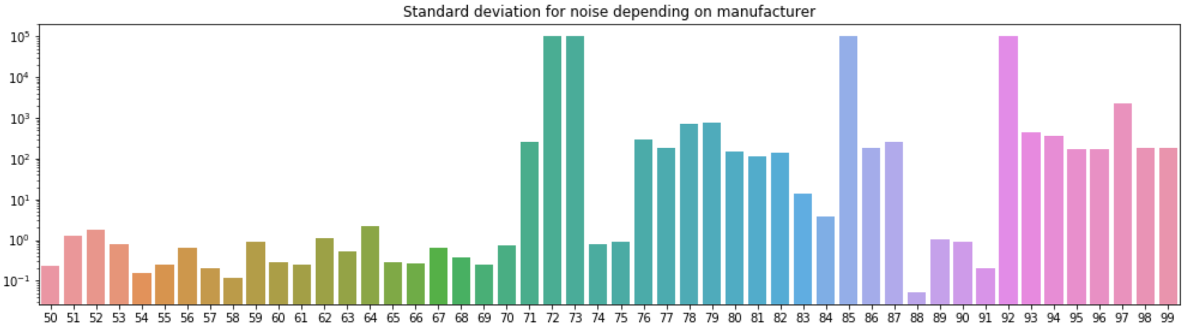
Standard deviation for noise depending on manufacturer
10^{-1} 10^{0} 10^{1} 10^{2} 10^{3} 10^{4} 10^{5}
50 51 52 53 54 55 56 57 58 59 60 61 62 63 64 65 66 67 68 69 70 71 72 73 74 75 76 77 78 79 80 81 82 83 84 85 86 87 88 89 90 91 92 93 94 95 96 97 98 99

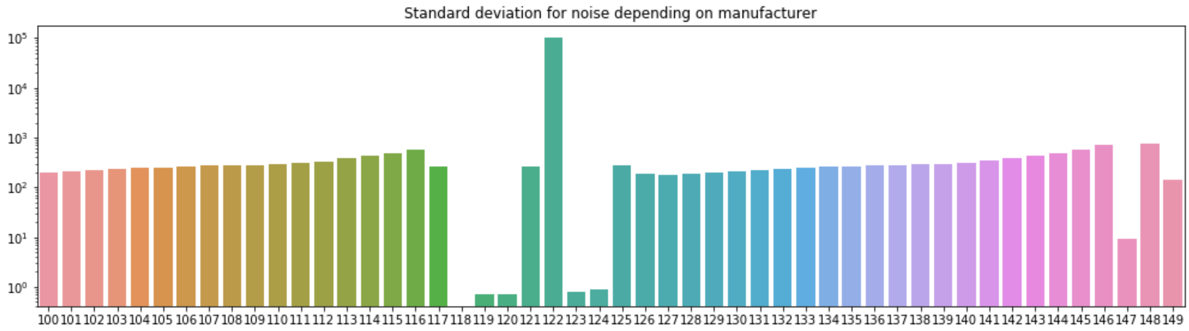
Standard deviation for noise depending on manufacturer
10^5 10^4 10^3 10^2 10^1 10^0
100 101 102 103 104 105 106 107 108 109 110 111 112 113 114 115 116 117 118 119 120 121 122 123 124 125 126 127 128 129 130 131 132 133 134 135 136 137 138 139 140 141 142 143 144 145 146 147 148 149

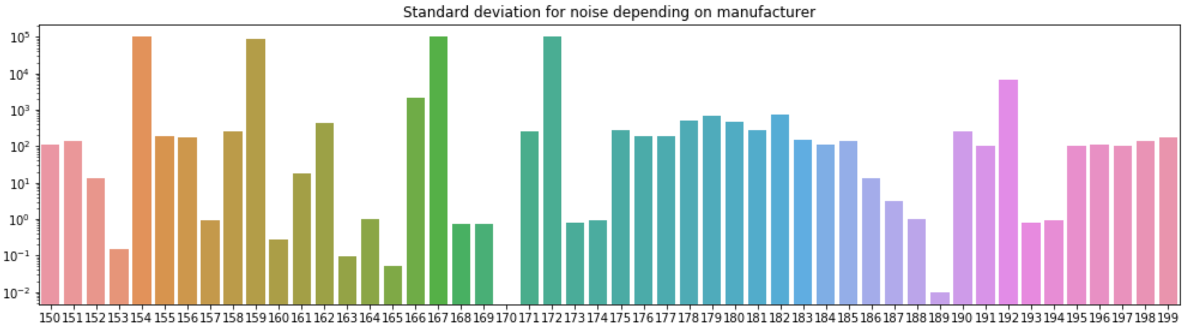
Standard deviation for noise depending on manufacturer
10^5 10^4 10^3 10^2 10^1 10^0 10^{-1} 10^{-2}
150 151 152 153 154 155 156 157 158 159 160 161 162 163 164 165 166 167 168 169 170 171 172 173 174 175 176 177 178 179 180 181 182 183 184 185 186 187 188 189 190 191 192 193 194 195 196 197 198 199

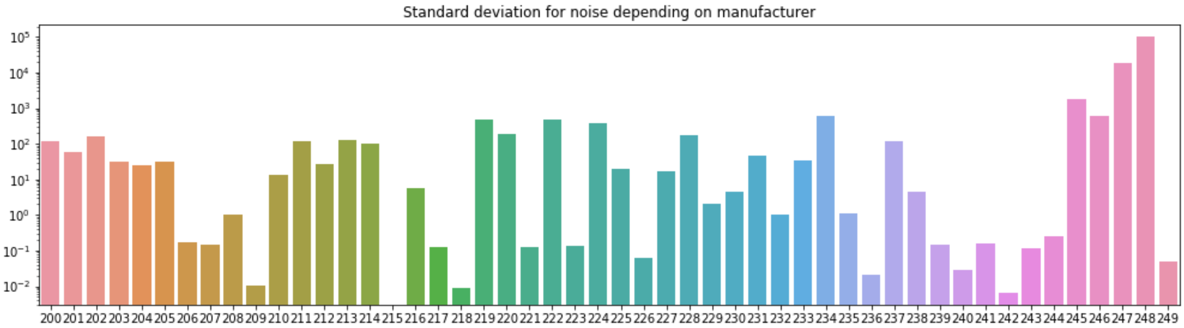
Standard deviation for noise depending on manufacturer
10^5 10^4 10^3 10^2 10^1 10^0 10^{-1} 10^{-2}
200 201 202 203 204 205 206 207 208 209 210 211 212 213 214 215 216 217 218 219 220 221 222 223 224 225 226 227 228 229 230 231 232 233 234 235 236 237 238 239 240 241 242 243 244 245 246 247 248 249

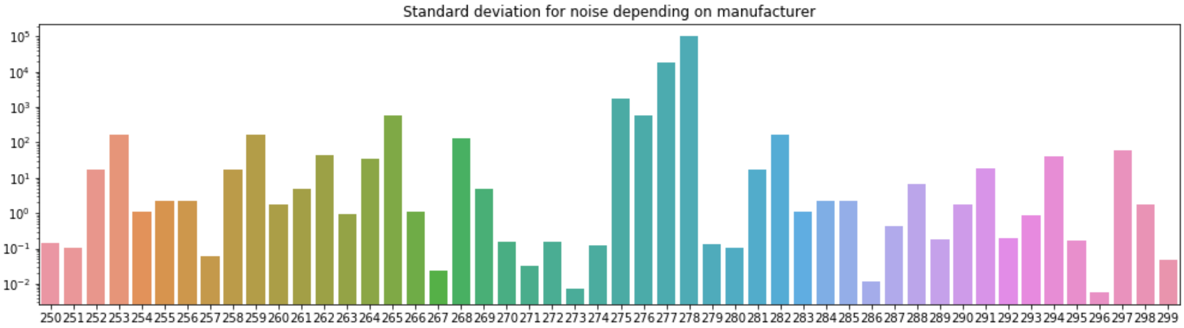
Standard deviation for noise depending on manufacturer
10^5 10^4 10^3 10^2 10^1 10^0 10^{-1} 10^{-2}
250 251 252 253 254 255 256 257 258 259 260 261 262 263 264 265 266 267 268 269 270 271 272 273 274 275 276 277 278 279 280 281 282 283 284 285 286 287 288 289 290 291 292 293 294 295 296 297 298 299

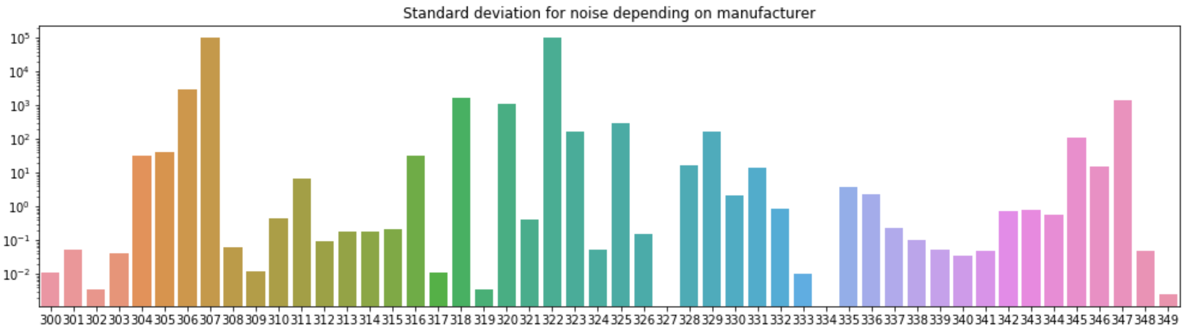

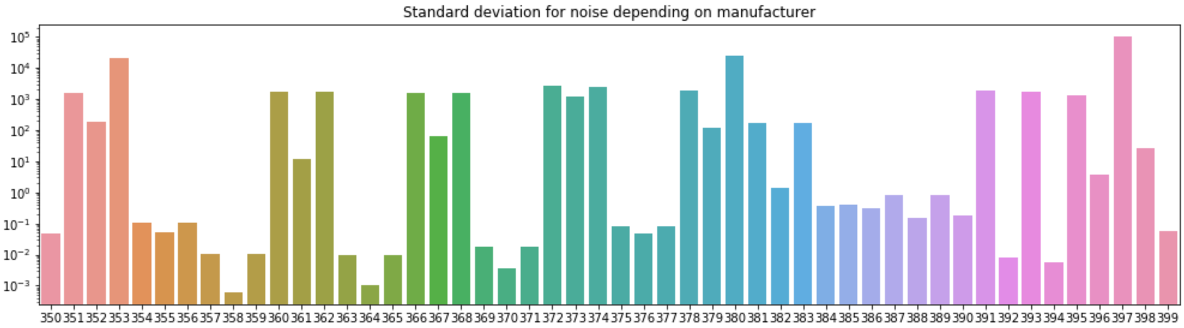

A10 Feature Noise Distributions for Voxelsize

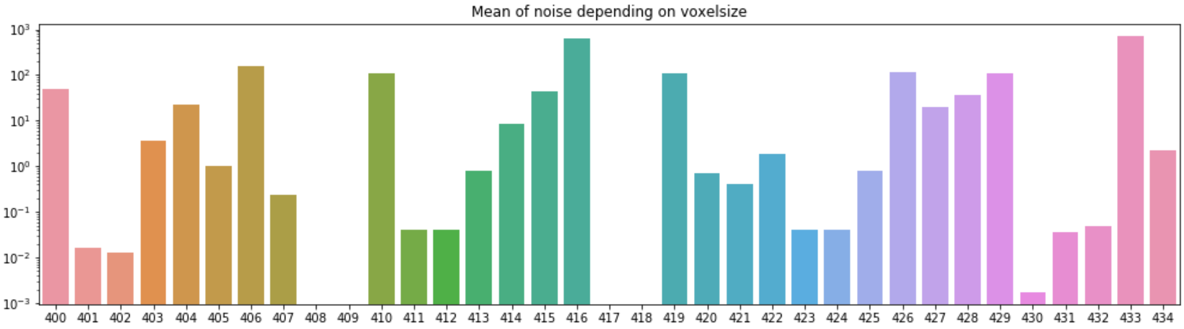

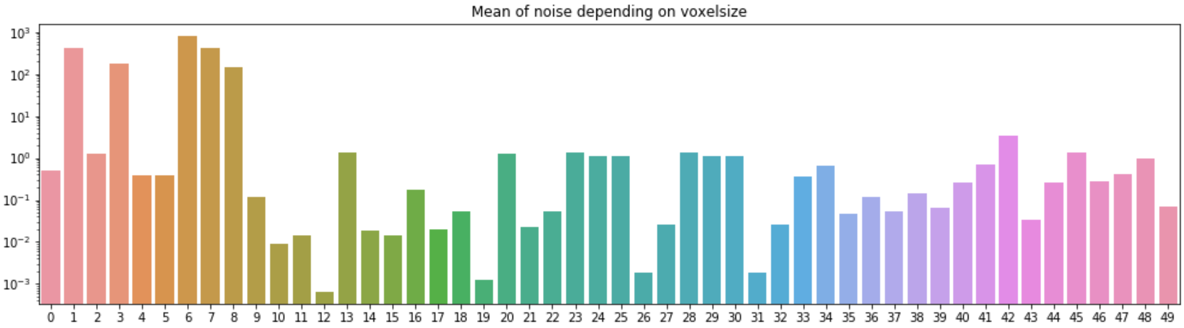

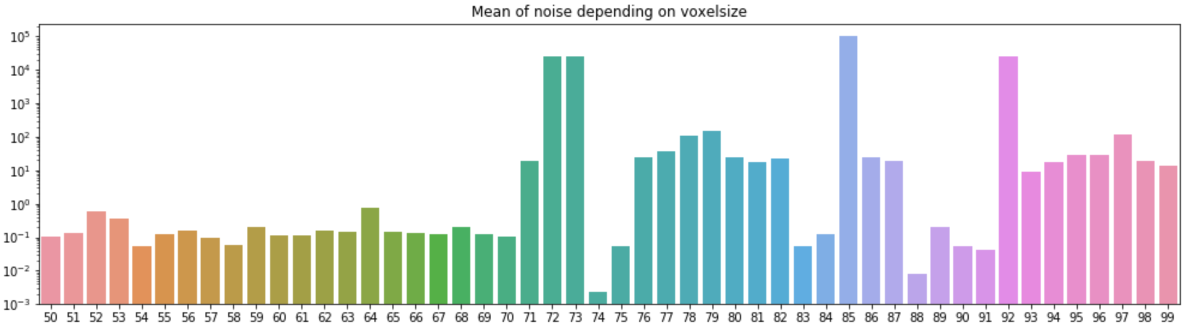

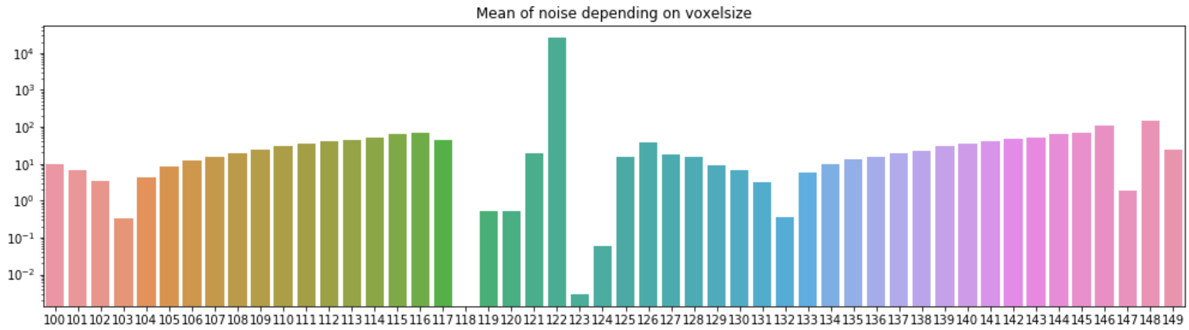
Mean of noise depending on voxelsize

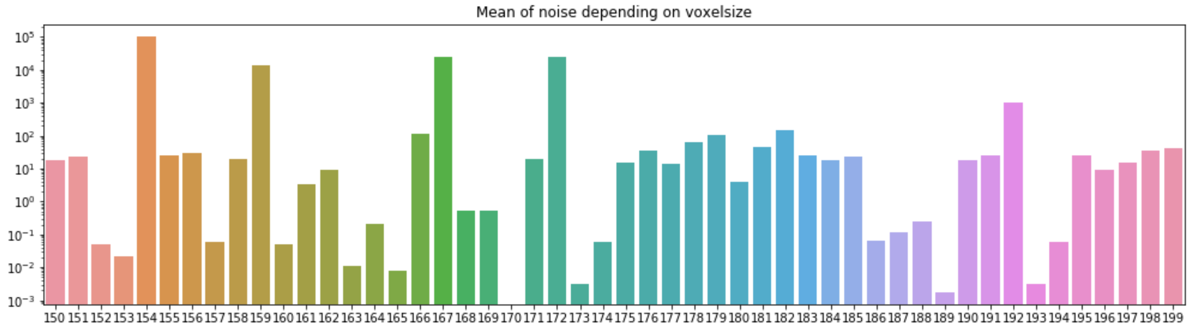
Mean of noise depending on voxelsize

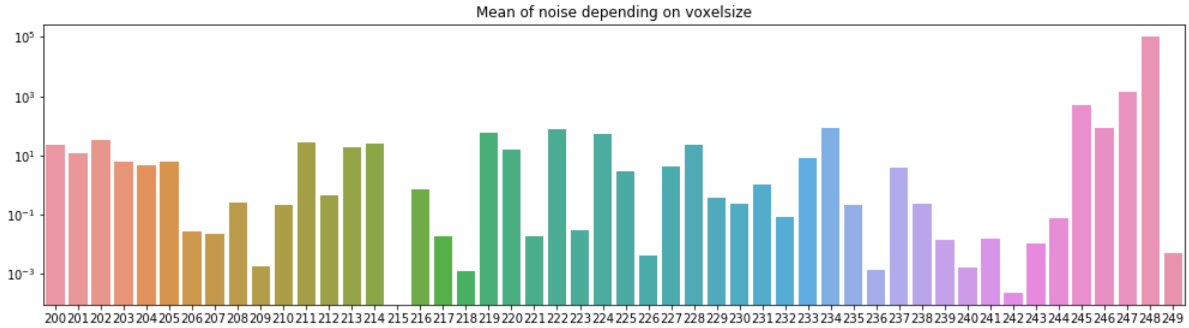
Mean of noise depending on voxelsize

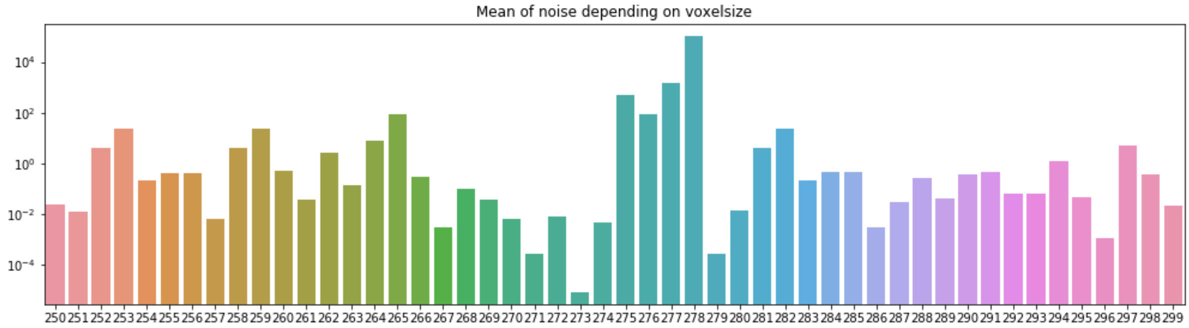
Mean of noise depending on voxelsize

Mean of noise depending on voxelsize

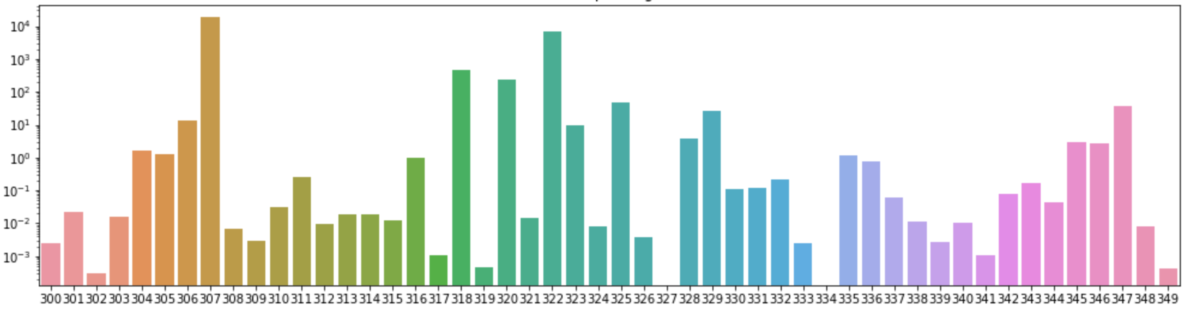

Mean of noise depending on voxelsize

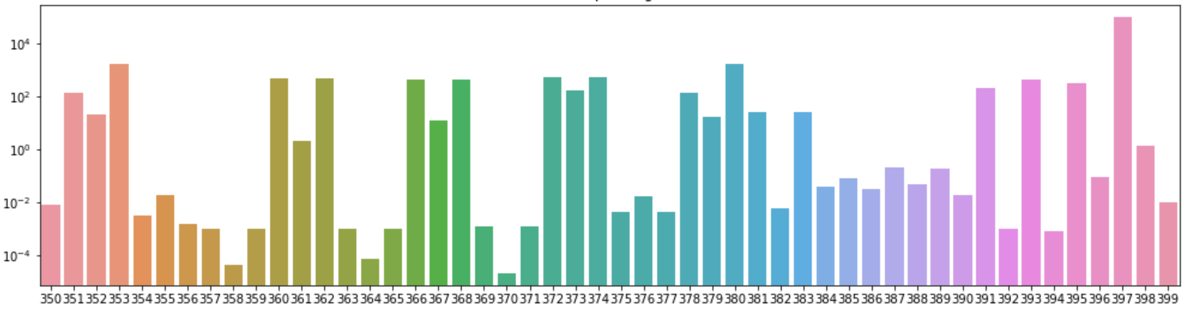

Standard deviation for noise depending on voxelsize

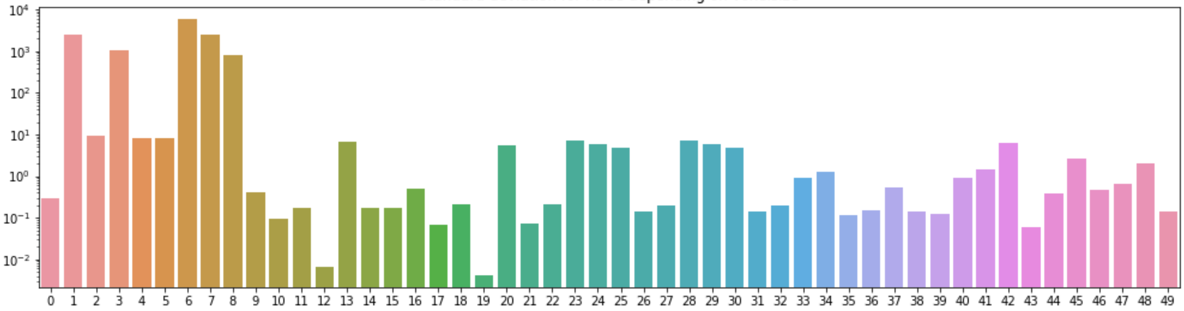

Standard deviation for noise depending on voxelsize

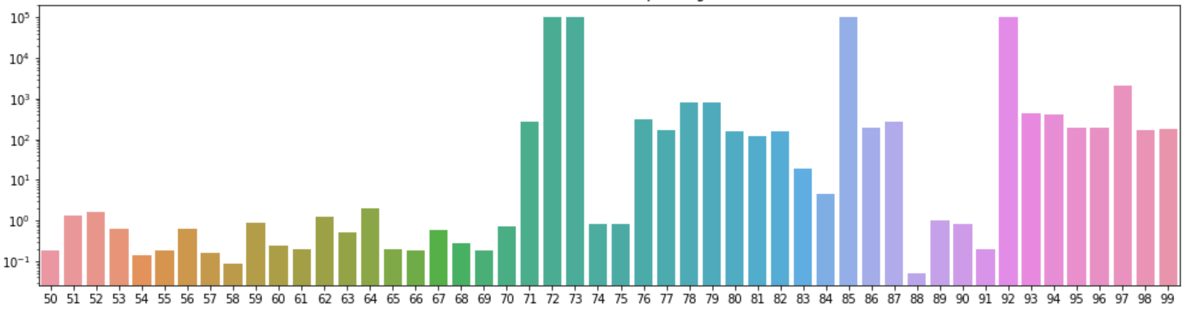

Standard deviation for noise depending on voxelsize

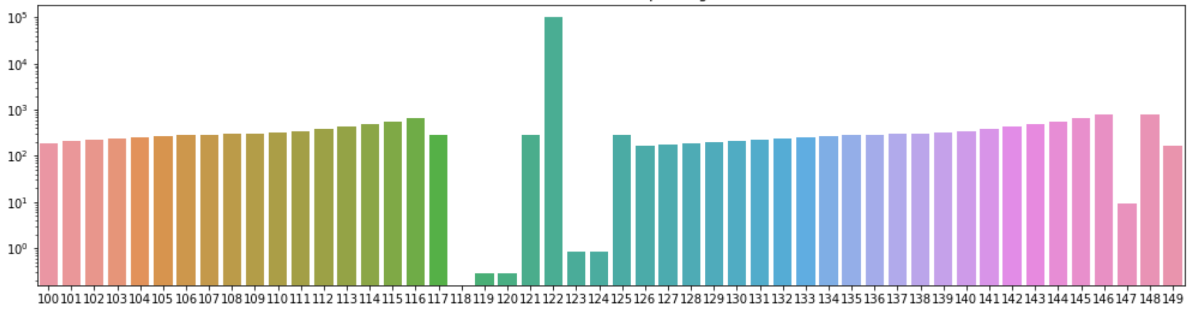

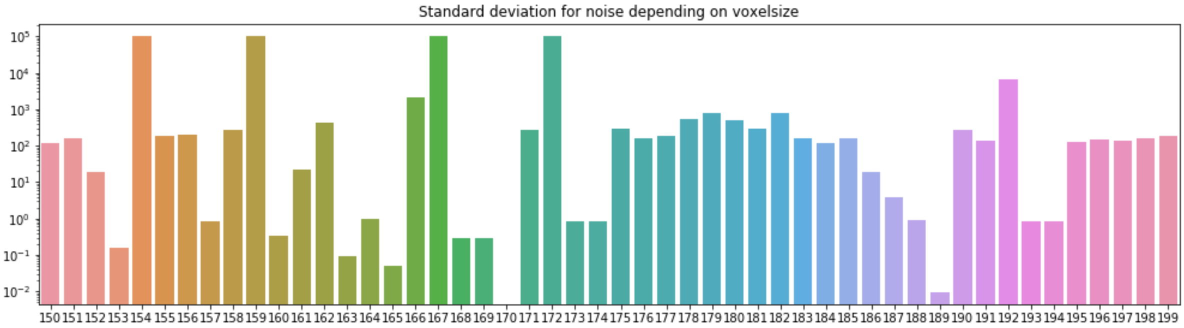
Standard deviation for noise depending on voxelsize
150 151 152 153 154 155 156 157 158 159 160 161 162 163 164 165 166 167 168 169 170 171 172 173 174 175 176 177 178 179 180 181 182 183 184 185 186 187 188 189 190 191 192 193 194 195 196 197 198 199

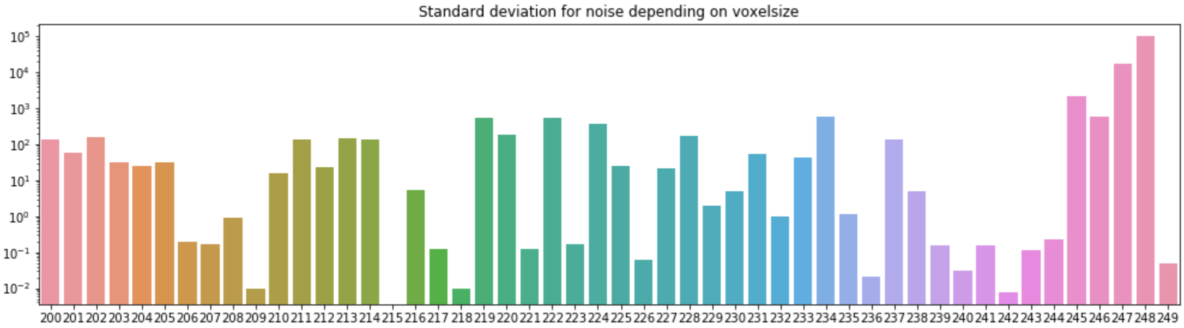
Standard deviation for noise depending on voxelsize
200 201 202 203 204 205 206 207 208 209 210 211 212 213 214 215 216 217 218 219 220 221 222 223 224 225 226 227 228 229 230 231 232 233 234 235 236 237 238 239 240 241 242 243 244 245 246 247 248 249

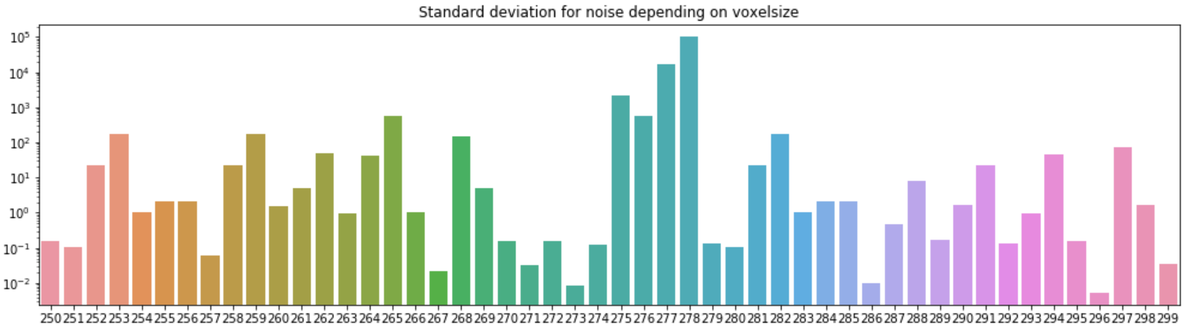
Standard deviation for noise depending on voxelsize
250 251 252 253 254 255 256 257 258 259 260 261 262 263 264 265 266 267 268 269 270 271 272 273 274 275 276 277 278 279 280 281 282 283 284 285 286 287 288 289 290 291 292 293 294 295 296 297 298 299

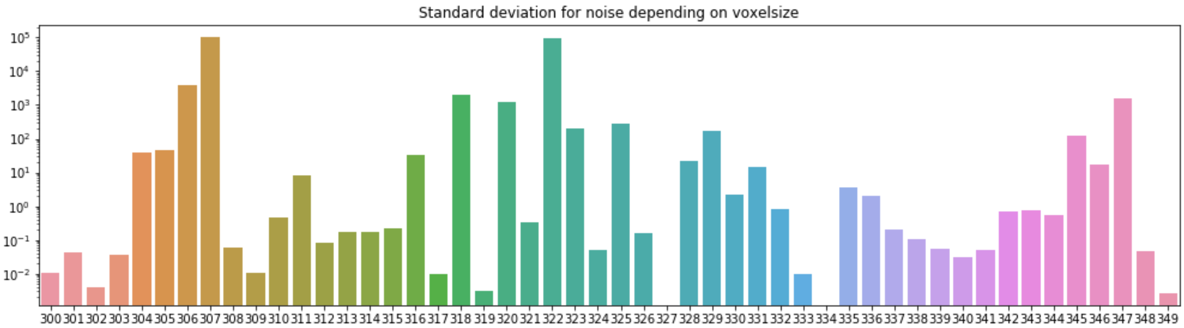
Standard deviation for noise depending on voxelsize
300 301 302 303 304 305 306 307 308 309 310 311 312 313 314 315 316 317 318 319 320 321 322 323 324 325 326 327 328 329 330 331 332 333 334 335 336 337 338 339 340 341 342 343 344 345 346 347 348 349

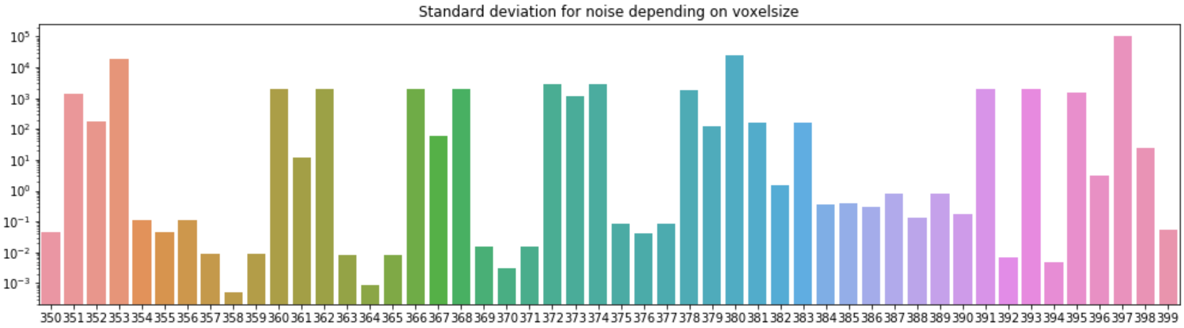
Standard deviation for noise depending on voxelsize

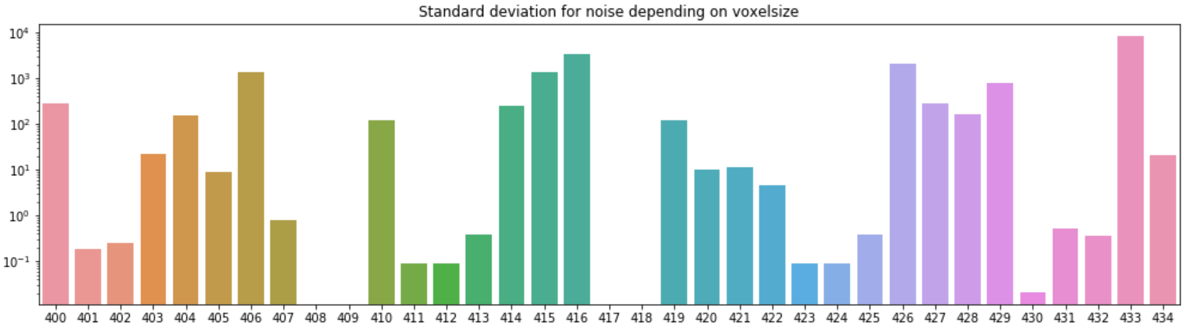
Standard deviation for noise depending on voxelsize

A11 Distribution of features w.r.t. subclasses

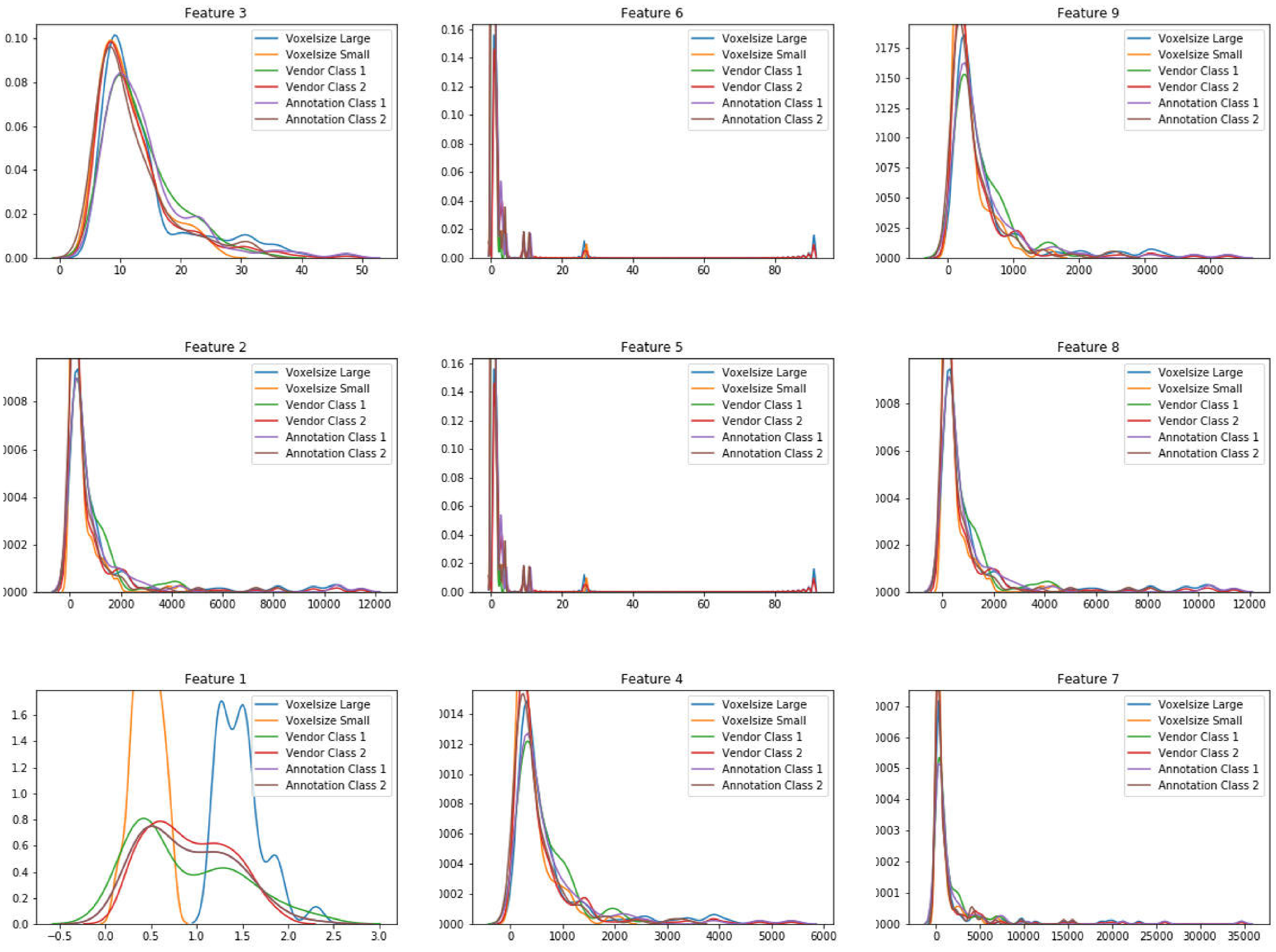
Feature 3
Feature 6
Feature 9
Feature 2
Feature 5
Feature 8
Feature 1
Feature 4
Feature 7
Voxelsize Large
Voxelsize Small
Vendor Class 1
Vendor Class 2
Annotation Class 1
Annotation Class 2

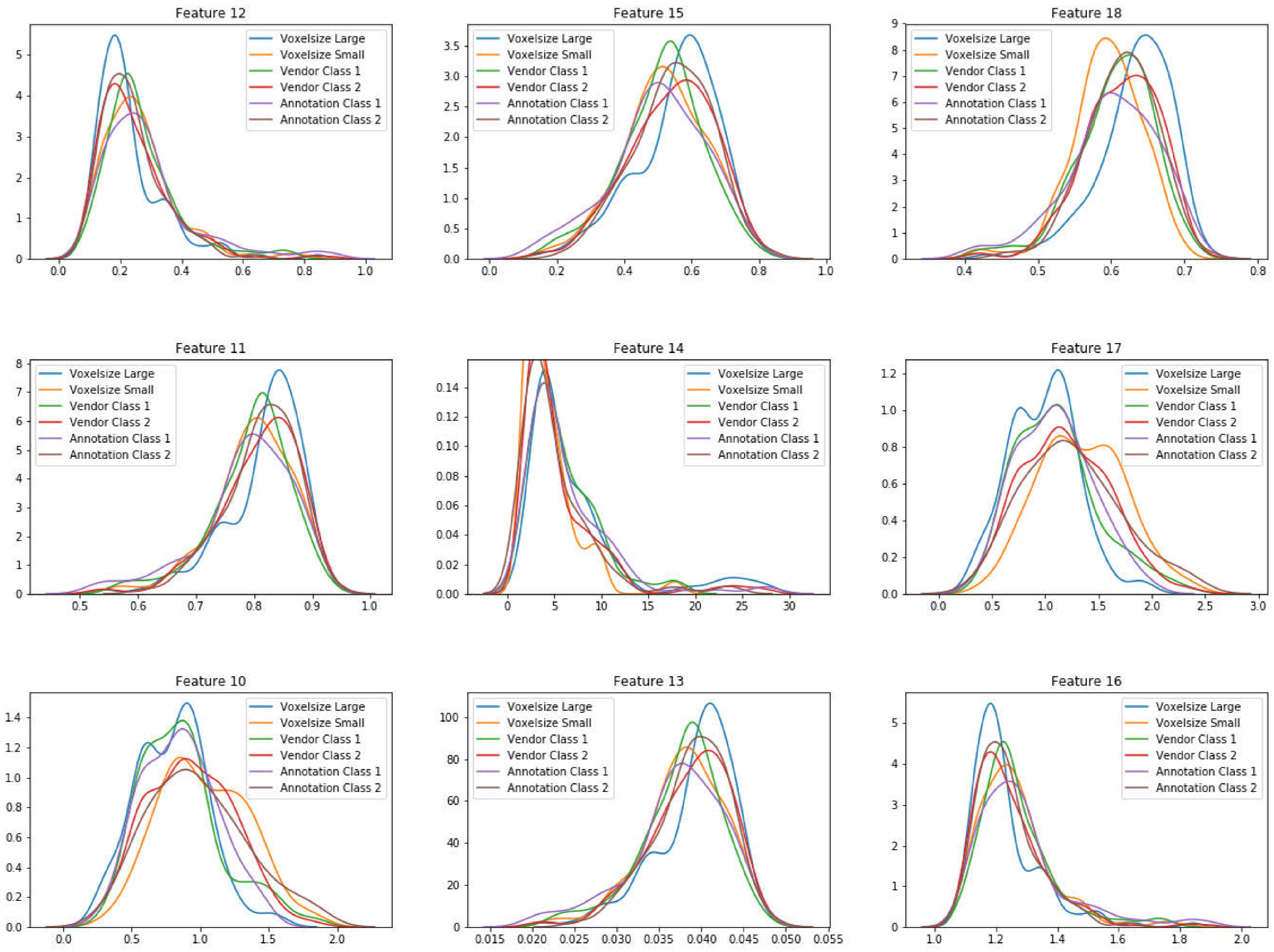
Feature 12
Voxelsize Large
Voxelsize Small
Vendor Class 1
Vendor Class 2
Annotation Class 1
Annotation Class 2
Feature 15
Voxelsize Large
Voxelsize Small
Vendor Class 1
Vendor Class 2
Annotation Class 1
Annotation Class 2
Feature 18
Voxelsize Large
Voxelsize Small
Vendor Class 1
Vendor Class 2
Annotation Class 1
Annotation Class 2
Feature 11
Voxelsize Large
Voxelsize Small
Vendor Class 1
Vendor Class 2
Annotation Class 1
Annotation Class 2
Feature 14
Voxelsize Large
Voxelsize Small
Vendor Class 1
Vendor Class 2
Annotation Class 1
Annotation Class 2
Feature 17
Voxelsize Large
Voxelsize Small
Vendor Class 1
Vendor Class 2
Annotation Class 1
Annotation Class 2
Feature 10
Voxelsize Large
Voxelsize Small
Vendor Class 1
Vendor Class 2
Annotation Class 1
Annotation Class 2
Feature 13
Voxelsize Large
Voxelsize Small
Vendor Class 1
Vendor Class 2
Annotation Class 1
Annotation Class 2
Feature 16
Voxelsize Large
Voxelsize Small
Vendor Class 1
Vendor Class 2
Annotation Class 1
Annotation Class 2

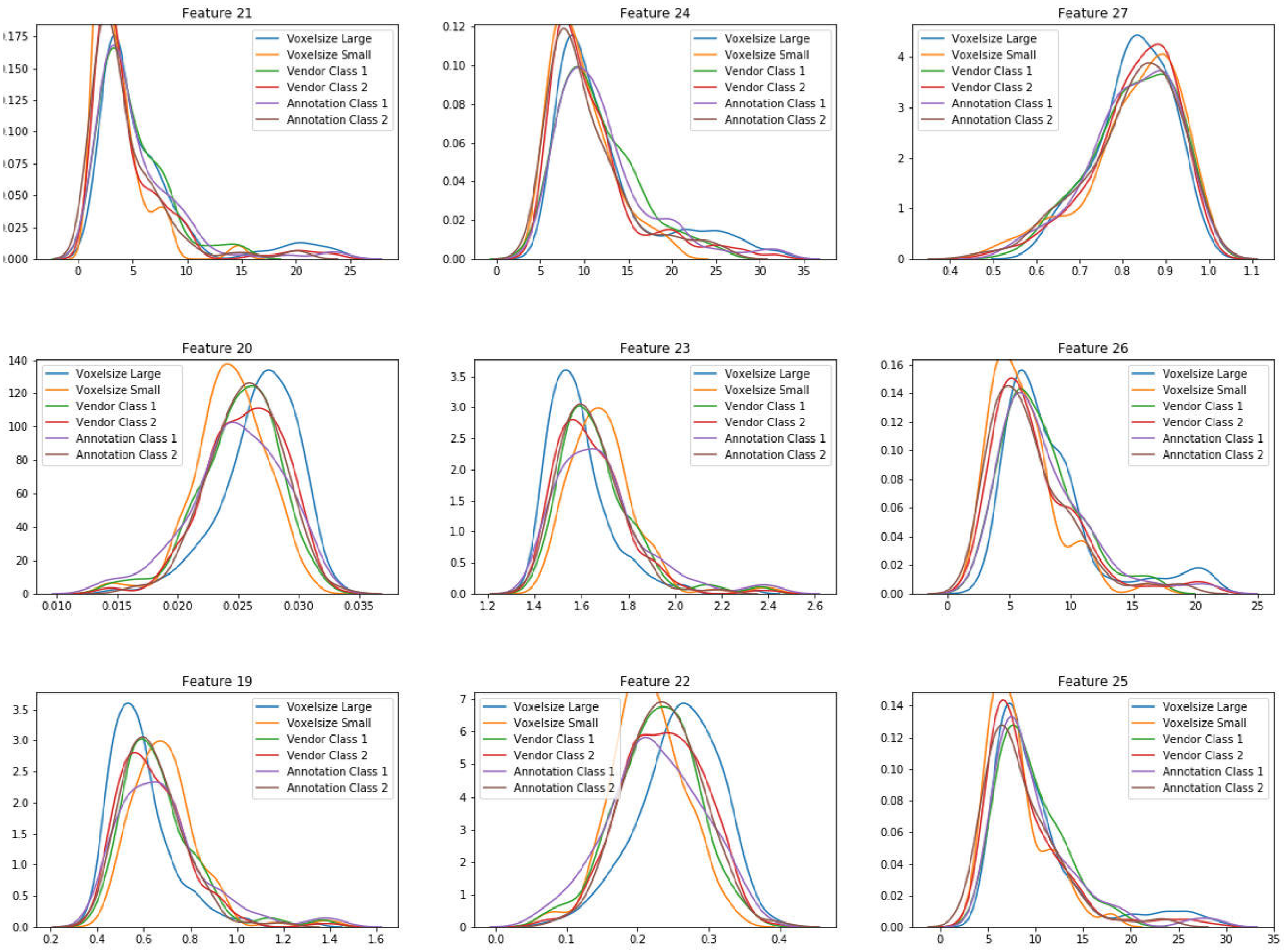
Feature 21
Feature 24
Feature 27
Feature 20
Feature 23
Feature 26
Feature 19
Feature 22
Feature 25
Voxelsize Large
Voxelsize Small
Vendor Class 1
Vendor Class 2
Annotation Class 1
Annotation Class 2

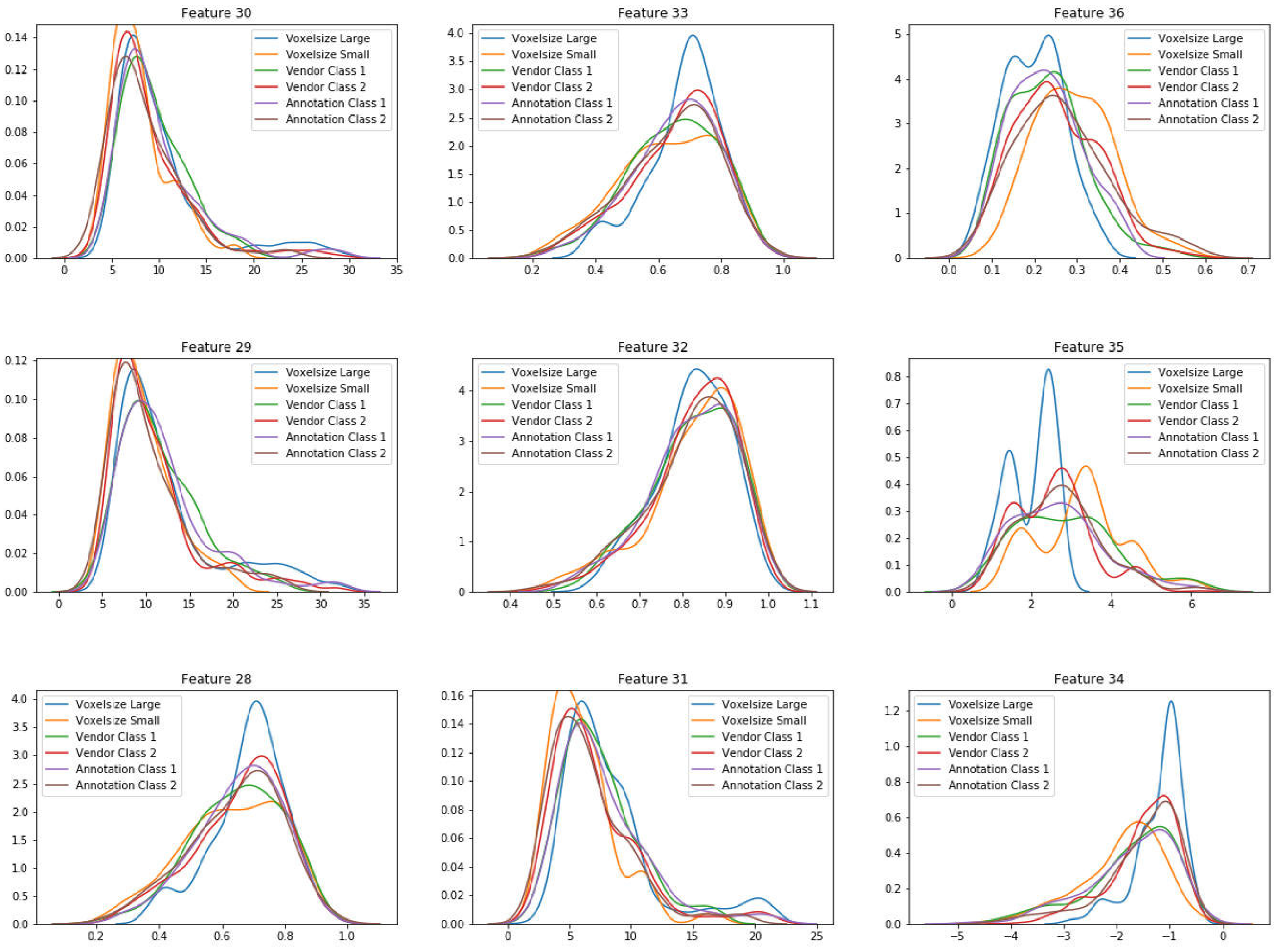
Feature 30
Voxelsize Large
Voxelsize Small
Vendor Class 1
Vendor Class 2
Annotation Class 1
Annotation Class 2
0.00 0.02 0.04 0.06 0.08 0.10 0.12 0.14
0 5 10 15 20 25 30 35
Feature 33
Voxelsize Large
Voxelsize Small
Vendor Class 1
Vendor Class 2
Annotation Class 1
Annotation Class 2
0.0 0.5 1.0 1.5 2.0 2.5 3.0 3.5 4.0
0.2 0.4 0.6 0.8 1.0
Feature 36
Voxelsize Large
Voxelsize Small
Vendor Class 1
Vendor Class 2
Annotation Class 1
Annotation Class 2
0 1 2 3 4 5
0.0 0.1 0.2 0.3 0.4 0.5 0.6 0.7
Feature 29
Voxelsize Large
Voxelsize Small
Vendor Class 1
Vendor Class 2
Annotation Class 1
Annotation Class 2
0.00 0.02 0.04 0.06 0.08 0.10 0.12
0 5 10 15 20 25 30 35
Feature 32
Voxelsize Large
Voxelsize Small
Vendor Class 1
Vendor Class 2
Annotation Class 1
Annotation Class 2
0 1 2 3 4
0.4 0.5 0.6 0.7 0.8 0.9 1.0 1.1
Feature 35
Voxelsize Large
Voxelsize Small
Vendor Class 1
Vendor Class 2
Annotation Class 1
Annotation Class 2
0.0 0.1 0.2 0.3 0.4 0.5 0.6 0.7 0.8
0 2 4 6
Feature 28
Voxelsize Large
Voxelsize Small
Vendor Class 1
Vendor Class 2
Annotation Class 1
Annotation Class 2
0.0 0.5 1.0 1.5 2.0 2.5 3.0 3.5 4.0
0.2 0.4 0.6 0.8 1.0
Feature 31
Voxelsize Large
Voxelsize Small
Vendor Class 1
Vendor Class 2
Annotation Class 1
Annotation Class 2
0.00 0.02 0.04 0.06 0.08 0.10 0.12 0.14 0.16
0 5 10 15 20 25
Feature 34
Voxelsize Large
Voxelsize Small
Vendor Class 1
Vendor Class 2
Annotation Class 1
Annotation Class 2
0.0 0.2 0.4 0.6 0.8 1.0 1.2
−5 −4 −3 −2 −1 0

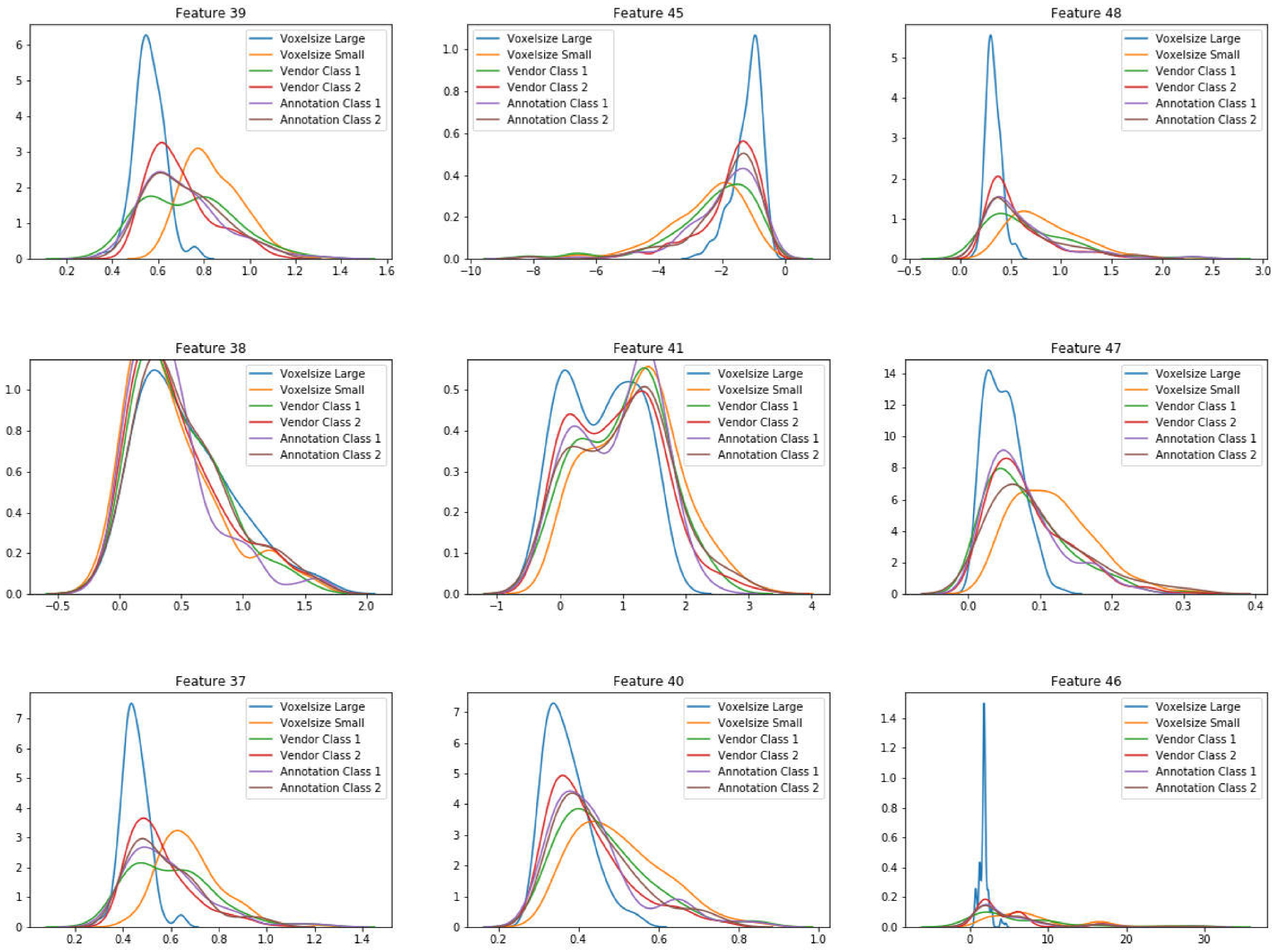

Feature 39
Feature 45
Feature 48
Feature 38
Feature 41
Feature 47
Feature 37
Feature 40
Feature 46
Voxelsize Large
Voxelsize Small
Vendor Class 1
Vendor Class 2
Annotation Class 1
Annotation Class 2

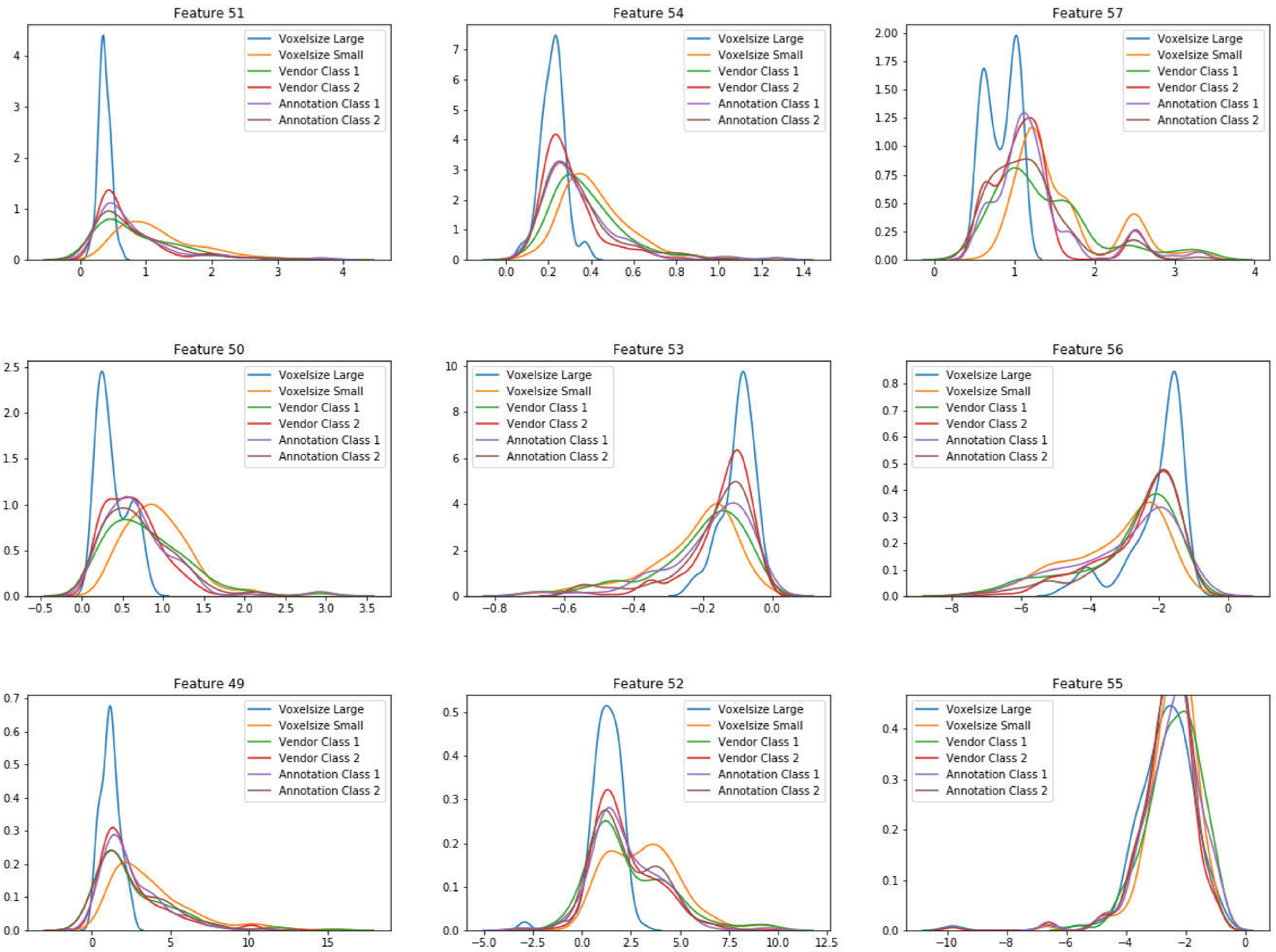
Feature 51
Voxelsize Large
Voxelsize Small
Vendor Class 1
Vendor Class 2
Annotation Class 1
Annotation Class 2
Feature 54
Voxelsize Large
Voxelsize Small
Vendor Class 1
Vendor Class 2
Annotation Class 1
Annotation Class 2
Feature 57
Voxelsize Large
Voxelsize Small
Vendor Class 1
Vendor Class 2
Annotation Class 1
Annotation Class 2
Feature 50
Voxelsize Large
Voxelsize Small
Vendor Class 1
Vendor Class 2
Annotation Class 1
Annotation Class 2
Feature 53
Voxelsize Large
Voxelsize Small
Vendor Class 1
Vendor Class 2
Annotation Class 1
Annotation Class 2
Feature 56
Voxelsize Large
Voxelsize Small
Vendor Class 1
Vendor Class 2
Annotation Class 1
Annotation Class 2
Feature 49
Voxelsize Large
Voxelsize Small
Vendor Class 1
Vendor Class 2
Annotation Class 1
Annotation Class 2
Feature 52
Voxelsize Large
Voxelsize Small
Vendor Class 1
Vendor Class 2
Annotation Class 1
Annotation Class 2
Feature 55
Voxelsize Large
Voxelsize Small
Vendor Class 1
Vendor Class 2
Annotation Class 1
Annotation Class 2

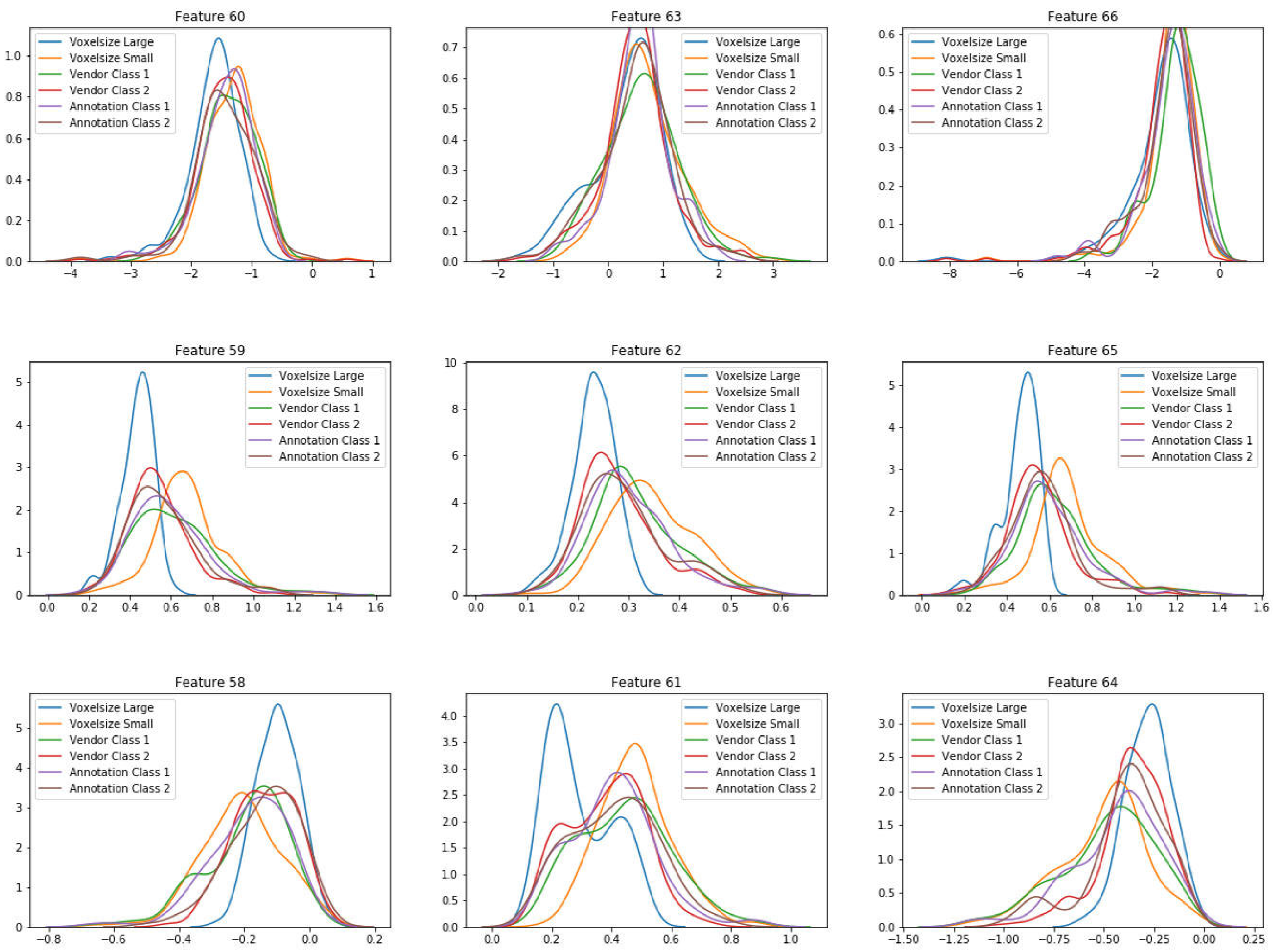
Feature 60
Feature 63
Feature 66
Feature 59
Feature 62
Feature 65
Feature 58
Feature 61
Feature 64
Voxelsize Large
Voxelsize Small
Vendor Class 1
Vendor Class 2
Annotation Class 1
Annotation Class 2

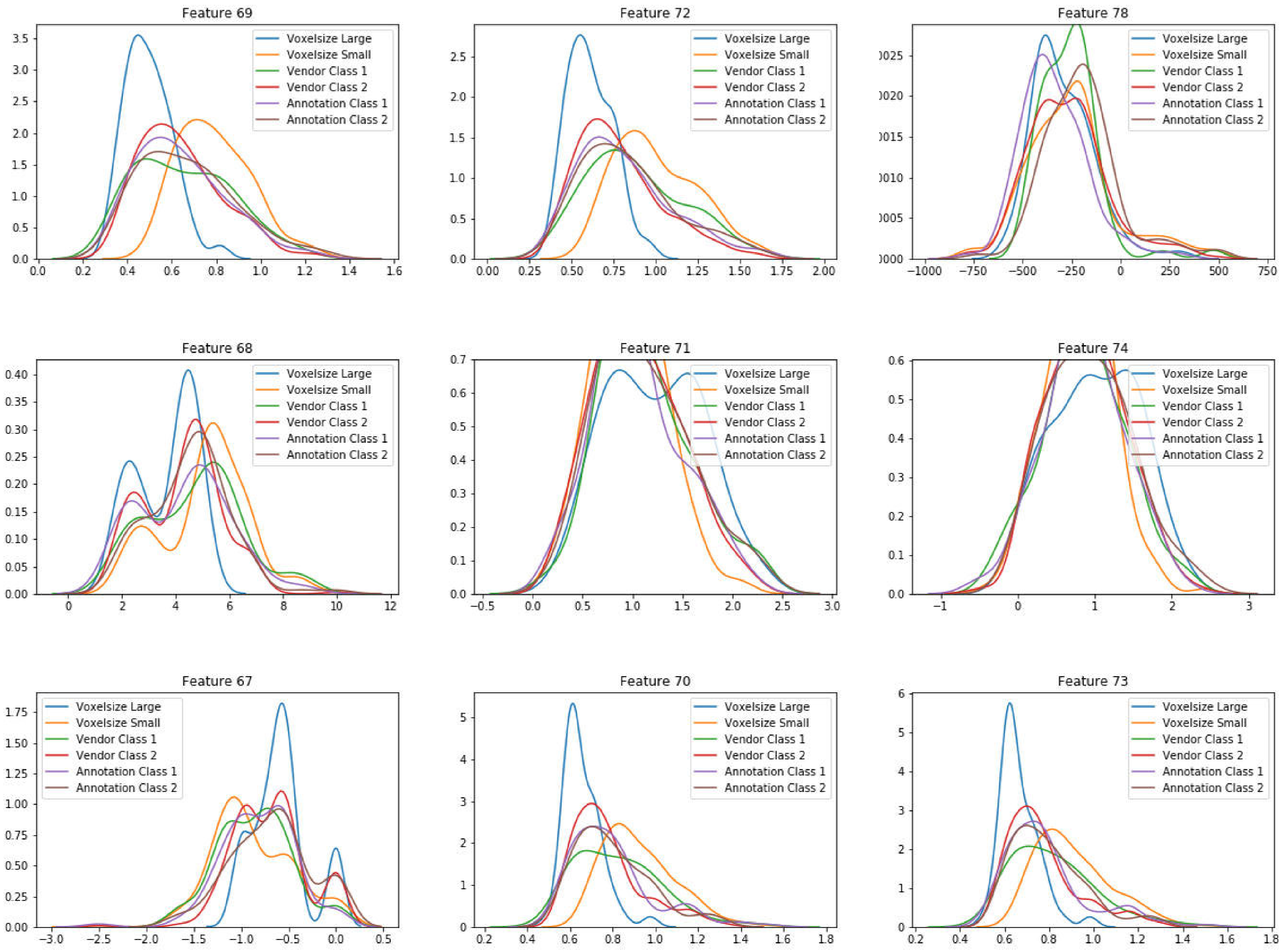
Feature 69
Feature 72
Feature 78
Feature 68
Feature 71
Feature 74
Feature 67
Feature 70
Feature 73
Voxelsize Large
Voxelsize Small
Vendor Class 1
Vendor Class 2
Annotation Class 1
Annotation Class 2

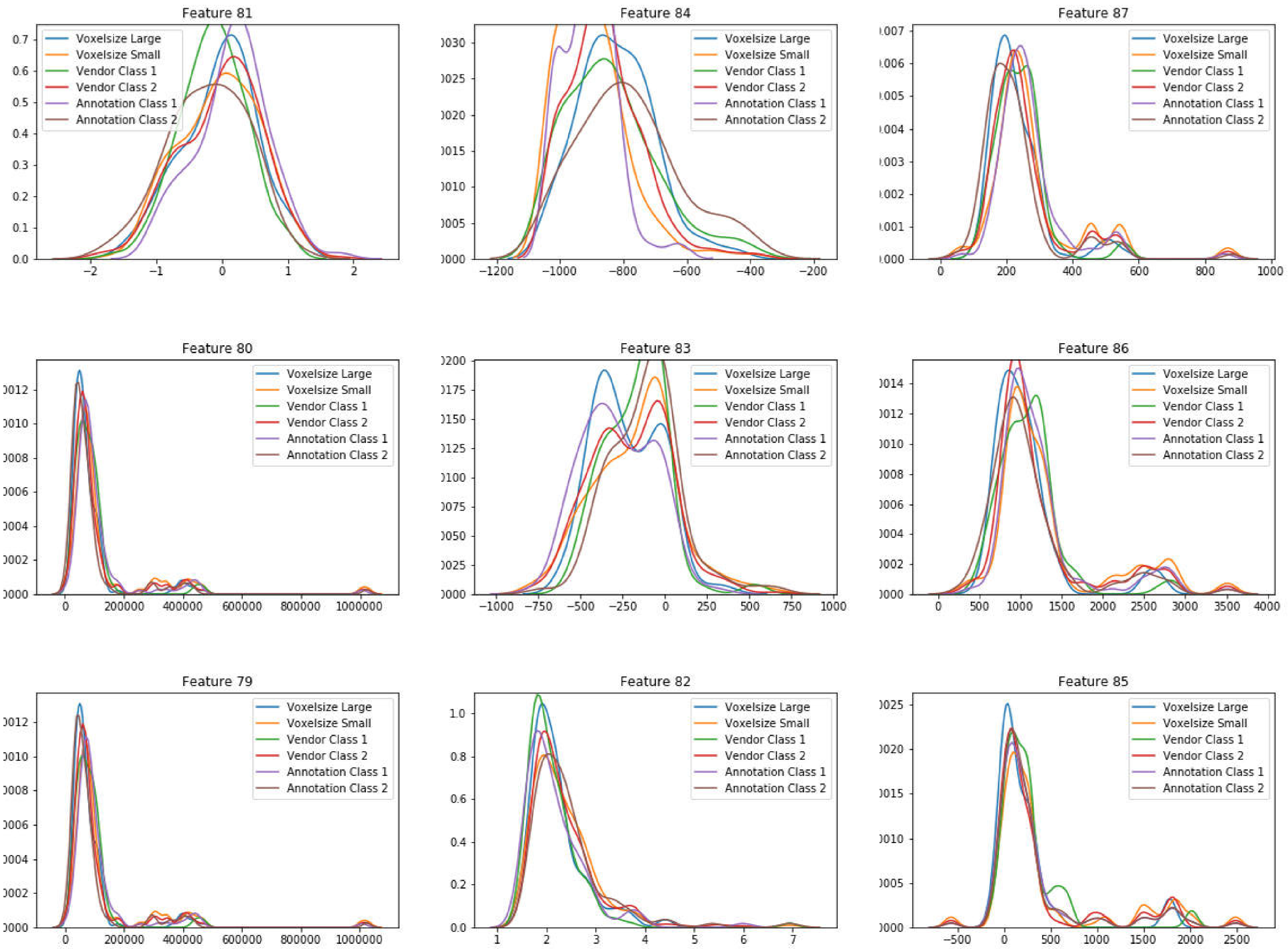

Feature 81
Feature 84
Feature 87
Feature 80
Feature 83
Feature 86
Feature 79
Feature 82
Feature 85
Voxelsize Large
Voxelsize Small
Vendor Class 1
Vendor Class 2
Annotation Class 1
Annotation Class 2

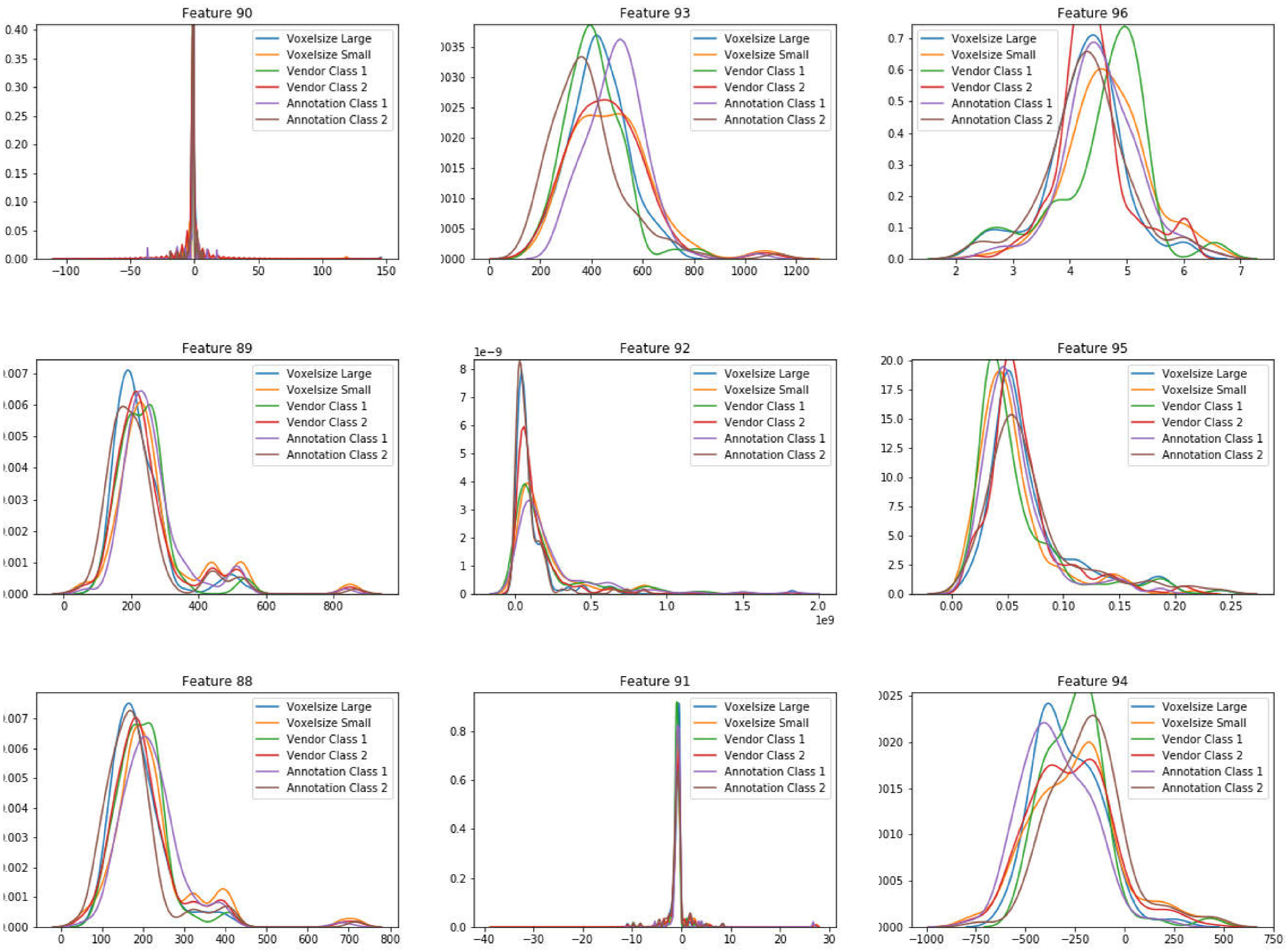
Feature 90
Feature 93
Feature 96
Feature 89
Feature 92
Feature 95
Feature 88
Feature 91
Feature 94
Voxelsize Large
Voxelsize Small
Vendor Class 1
Vendor Class 2
Annotation Class 1
Annotation Class 2
1e−9
1e9

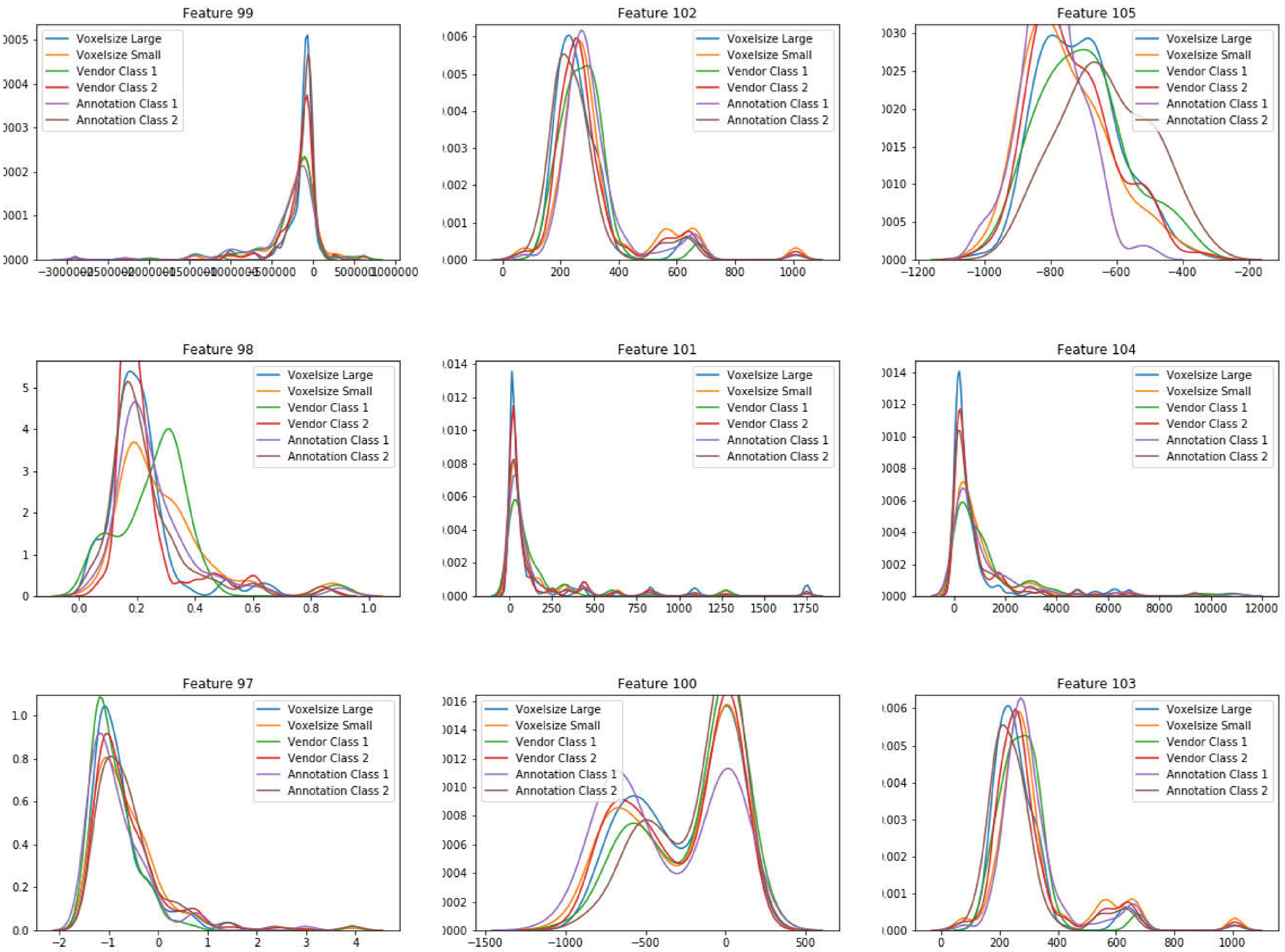
Feature 99
Feature 102
Feature 105
Feature 98
Feature 101
Feature 104
Feature 97
Feature 100
Feature 103
Voxelsize Large
Voxelsize Small
Vendor Class 1
Vendor Class 2
Annotation Class 1
Annotation Class 2

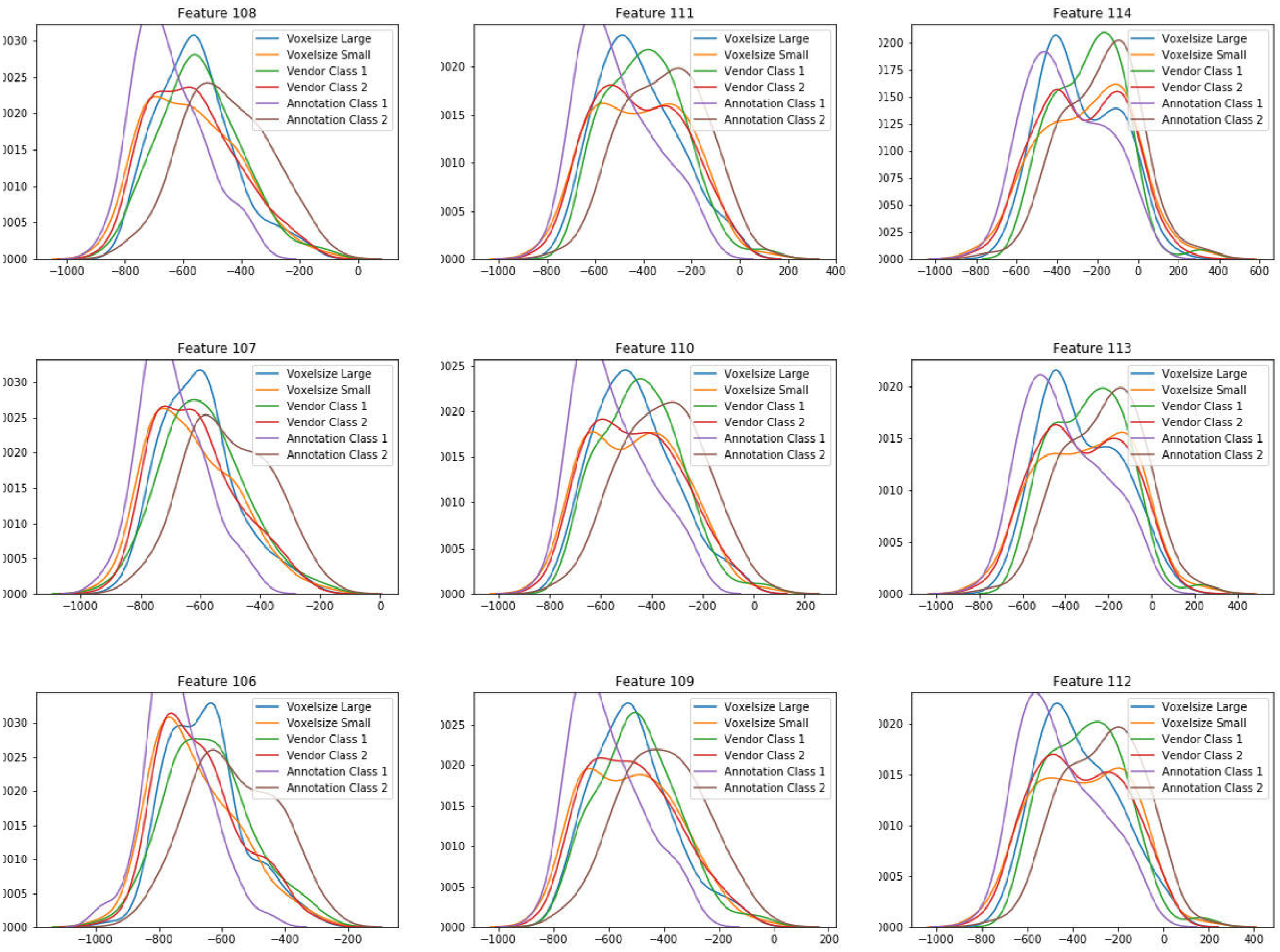
Feature 108
Feature 111
Feature 114
Feature 107
Feature 110
Feature 113
Feature 106
Feature 109
Feature 112
Voxelsize Large
Voxelsize Small
Vendor Class 1
Vendor Class 2
Annotation Class 1
Annotation Class 2

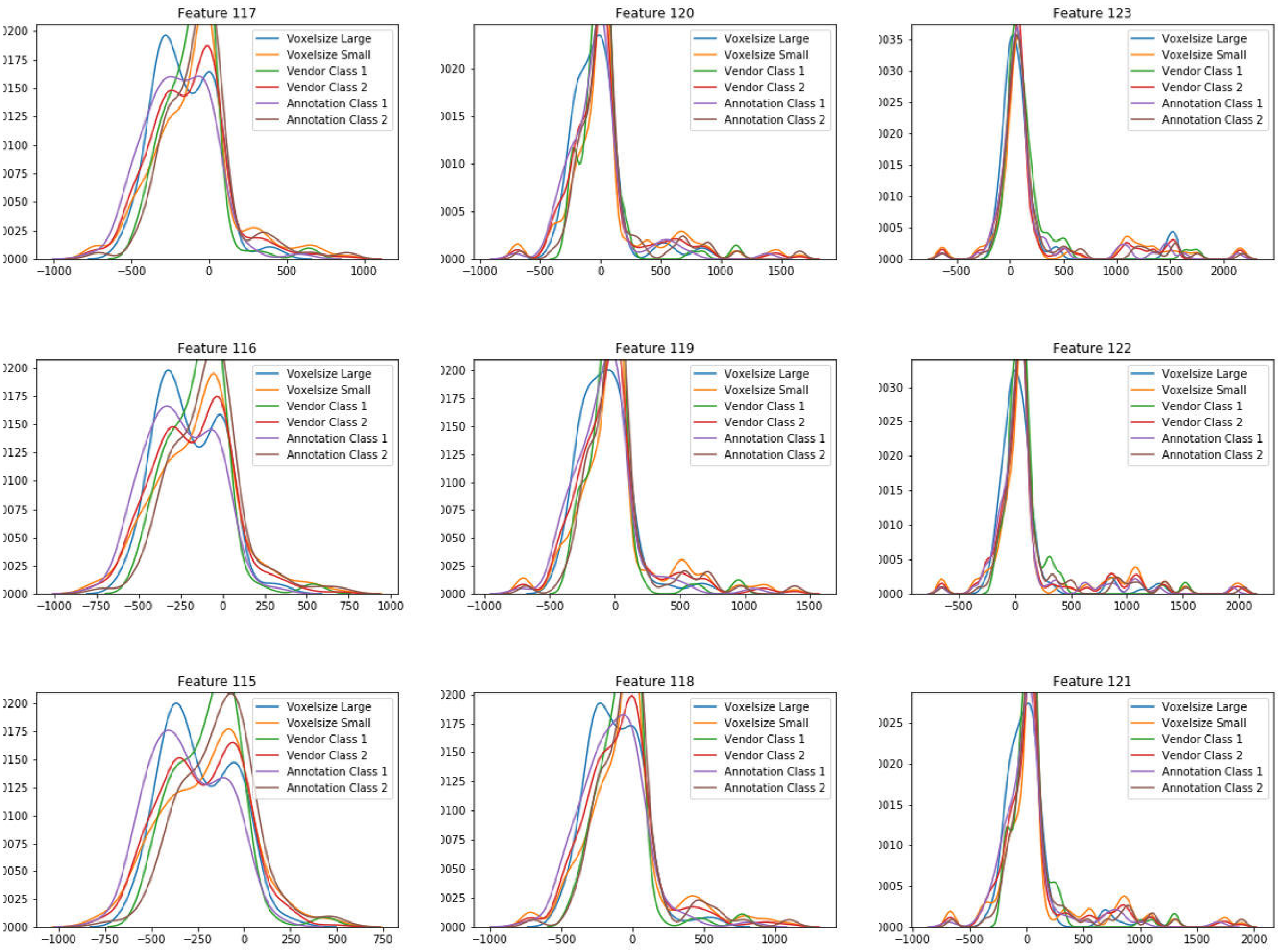

Feature 117
Feature 120
Feature 123
Feature 116
Feature 119
Feature 122
Feature 115
Feature 118
Feature 121
Voxelsize Large
Voxelsize Small
Vendor Class 1
Vendor Class 2
Annotation Class 1
Annotation Class 2

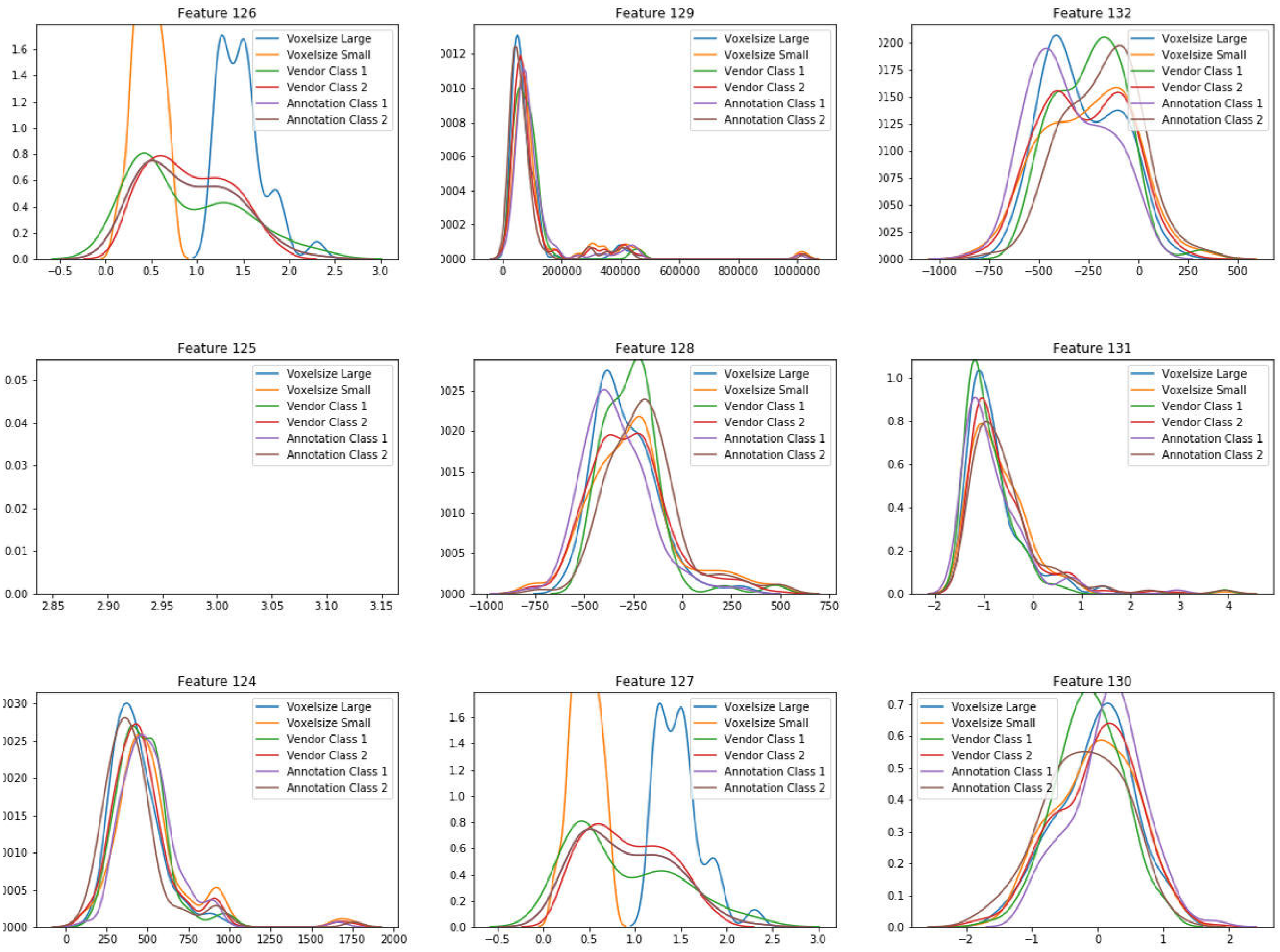
Feature 126
Feature 129
Feature 132
Feature 125
Feature 128
Feature 131
Feature 124
Feature 127
Feature 130
Voxelsize Large
Voxelsize Small
Vendor Class 1
Vendor Class 2
Annotation Class 1
Annotation Class 2

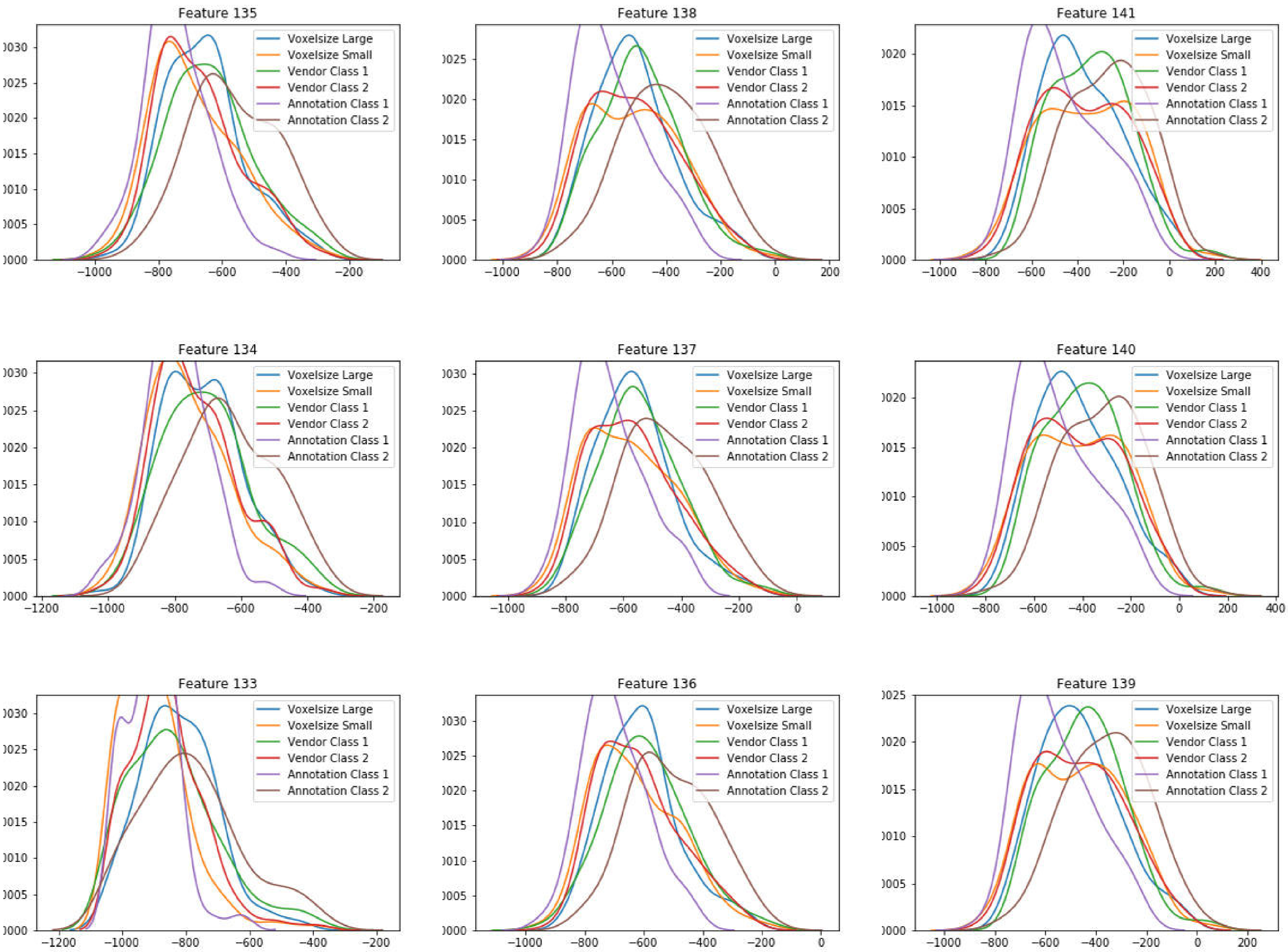
Feature 135
Feature 138
Feature 141
Feature 134
Feature 137
Feature 140
Feature 133
Feature 136
Feature 139
Voxelsize Large
Voxelsize Small
Vendor Class 1
Vendor Class 2
Annotation Class 1
Annotation Class 2

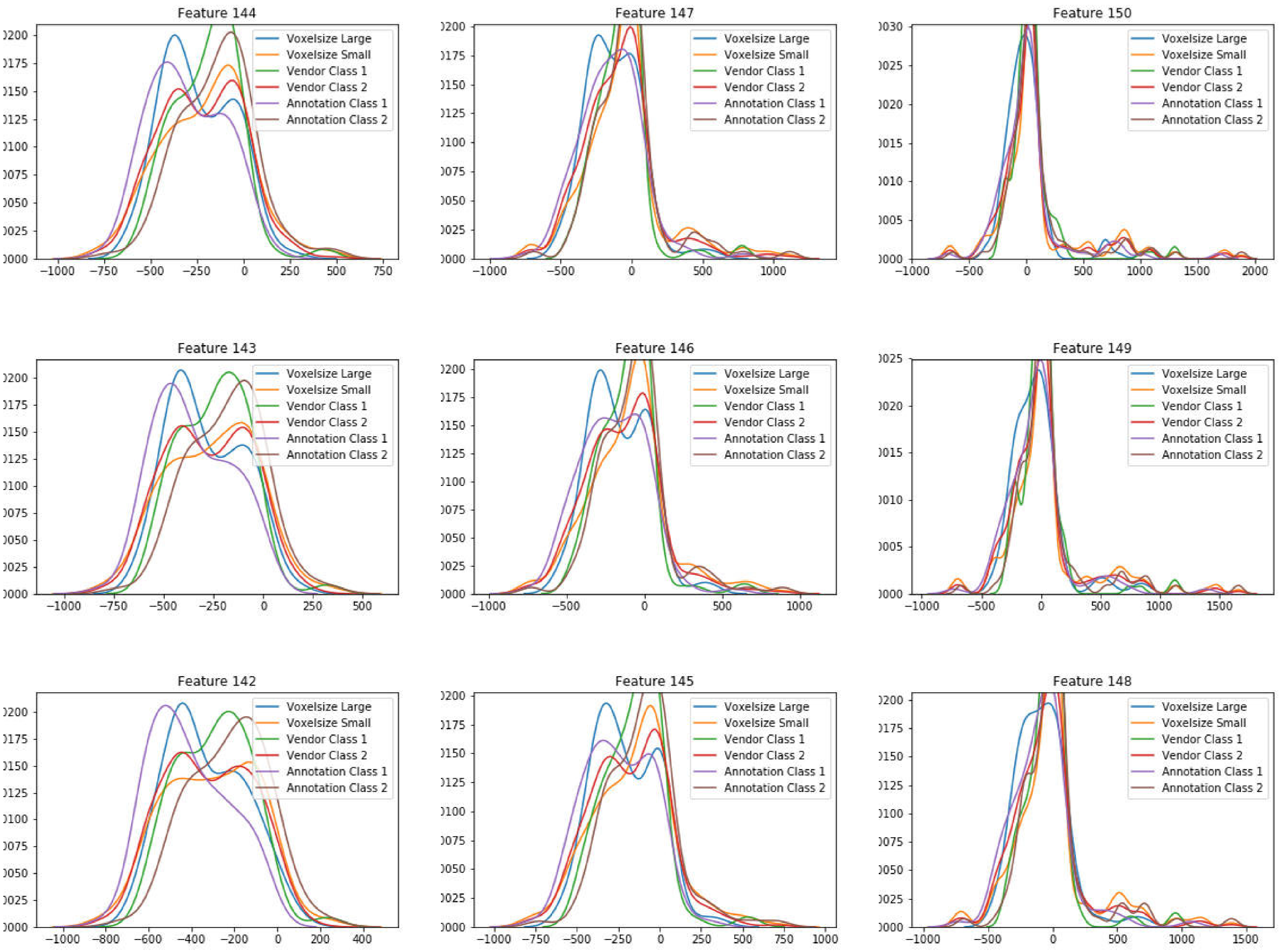
Feature 144
Feature 147
Feature 150
Feature 143
Feature 146
Feature 149
Feature 142
Feature 145
Feature 148
Voxelsize Large
Voxelsize Small
Vendor Class 1
Vendor Class 2
Annotation Class 1
Annotation Class 2

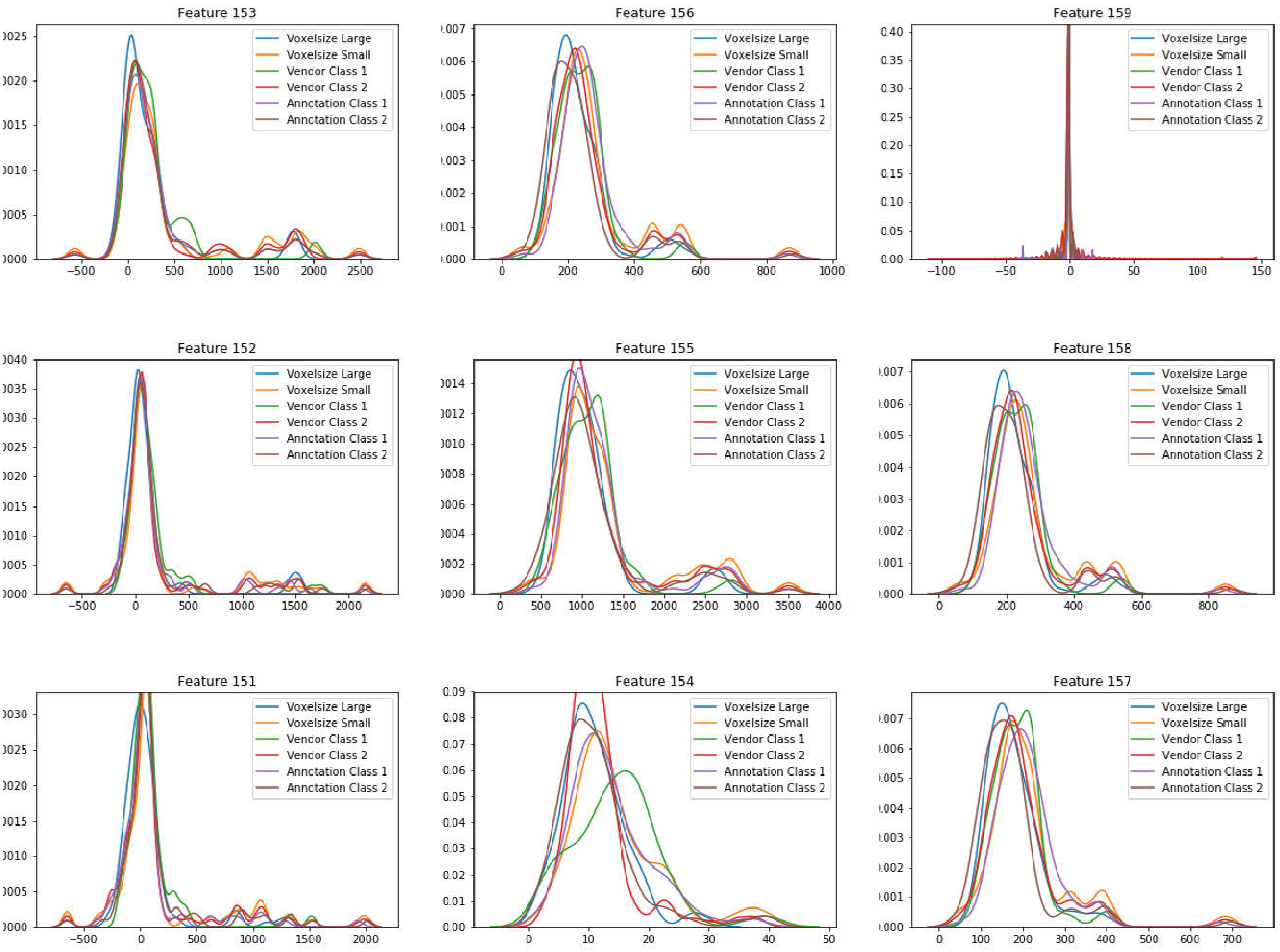
Feature 153
Feature 156
Feature 159
Feature 152
Feature 155
Feature 158
Feature 151
Feature 154
Feature 157
Voxelsize Large
Voxelsize Small
Vendor Class 1
Vendor Class 2
Annotation Class 1
Annotation Class 2

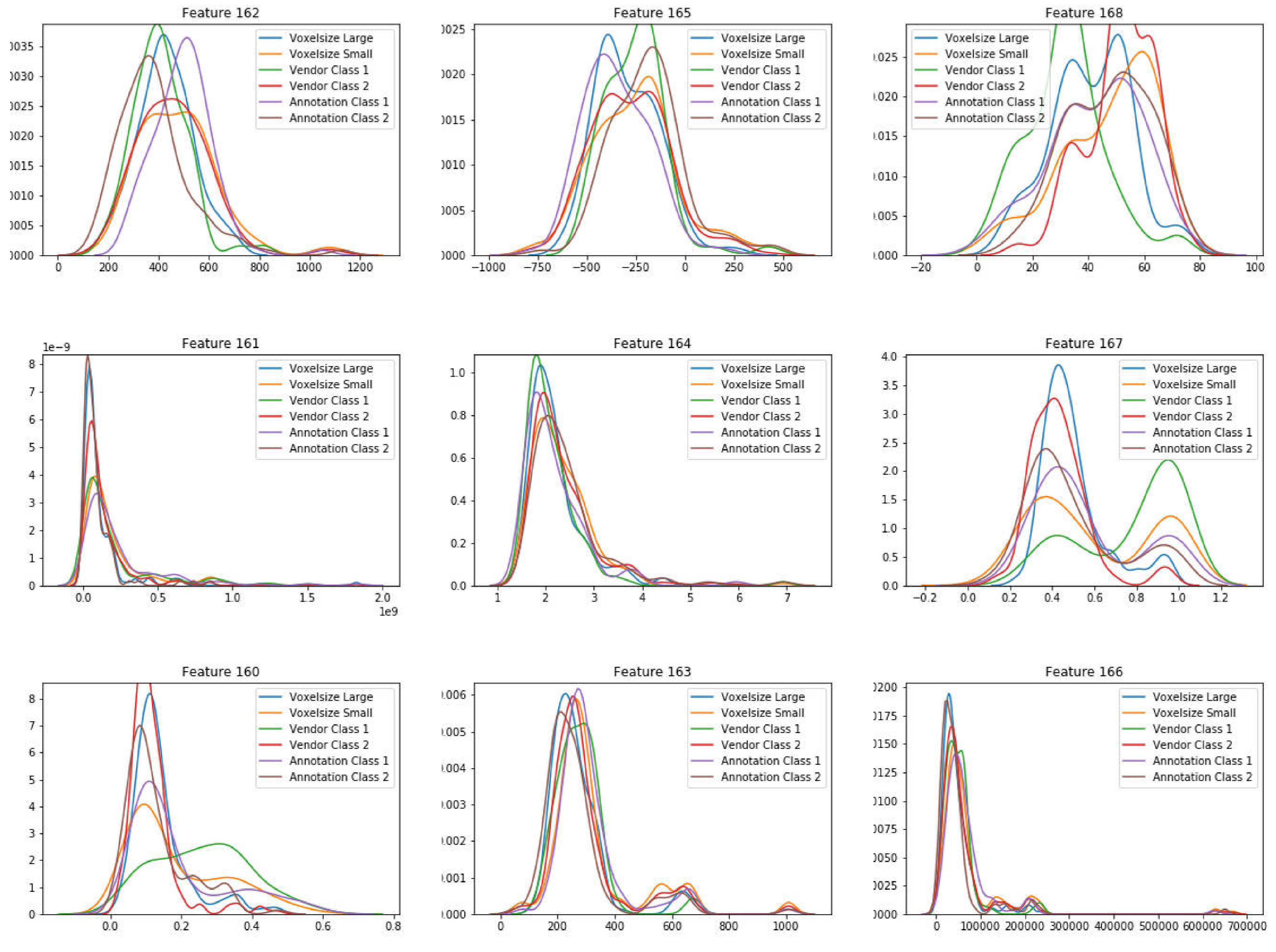

Feature 162
Feature 165
Feature 168
Feature 161
Feature 164
Feature 167
Feature 160
Feature 163
Feature 166
Voxelsize Large
Voxelsize Small
Vendor Class 1
Vendor Class 2
Annotation Class 1
Annotation Class 2
1e−9
1e9

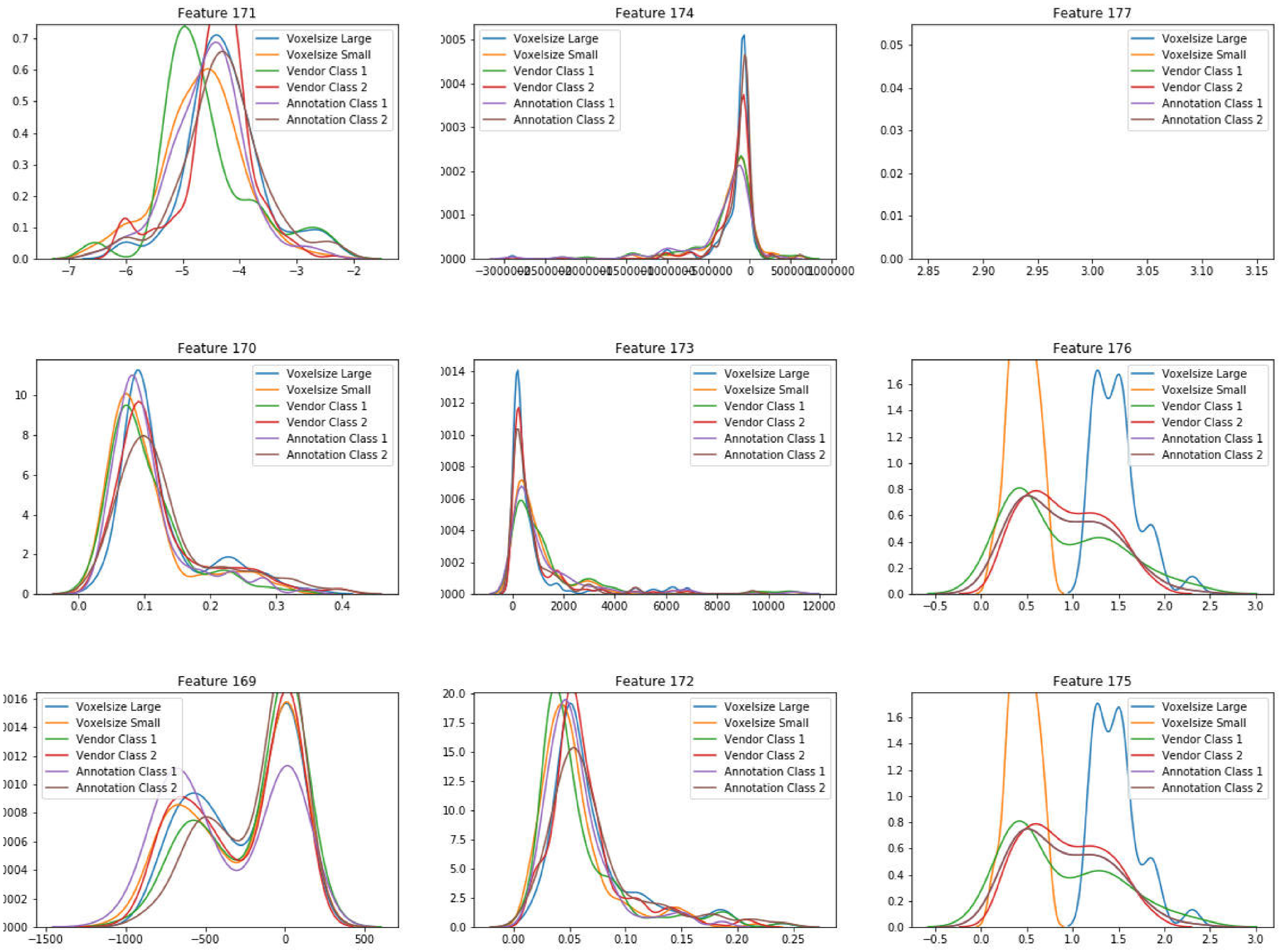
Feature 171
Voxelsize Large
Voxelsize Small
Vendor Class 1
Vendor Class 2
Annotation Class 1
Annotation Class 2
Feature 174
Voxelsize Large
Voxelsize Small
Vendor Class 1
Vendor Class 2
Annotation Class 1
Annotation Class 2
Feature 177
Voxelsize Large
Voxelsize Small
Vendor Class 1
Vendor Class 2
Annotation Class 1
Annotation Class 2
Feature 170
Voxelsize Large
Voxelsize Small
Vendor Class 1
Vendor Class 2
Annotation Class 1
Annotation Class 2
Feature 173
Voxelsize Large
Voxelsize Small
Vendor Class 1
Vendor Class 2
Annotation Class 1
Annotation Class 2
Feature 176
Voxelsize Large
Voxelsize Small
Vendor Class 1
Vendor Class 2
Annotation Class 1
Annotation Class 2
Feature 169
Voxelsize Large
Voxelsize Small
Vendor Class 1
Vendor Class 2
Annotation Class 1
Annotation Class 2
Feature 172
Voxelsize Large
Voxelsize Small
Vendor Class 1
Vendor Class 2
Annotation Class 1
Annotation Class 2
Feature 175
Voxelsize Large
Voxelsize Small
Vendor Class 1
Vendor Class 2
Annotation Class 1
Annotation Class 2

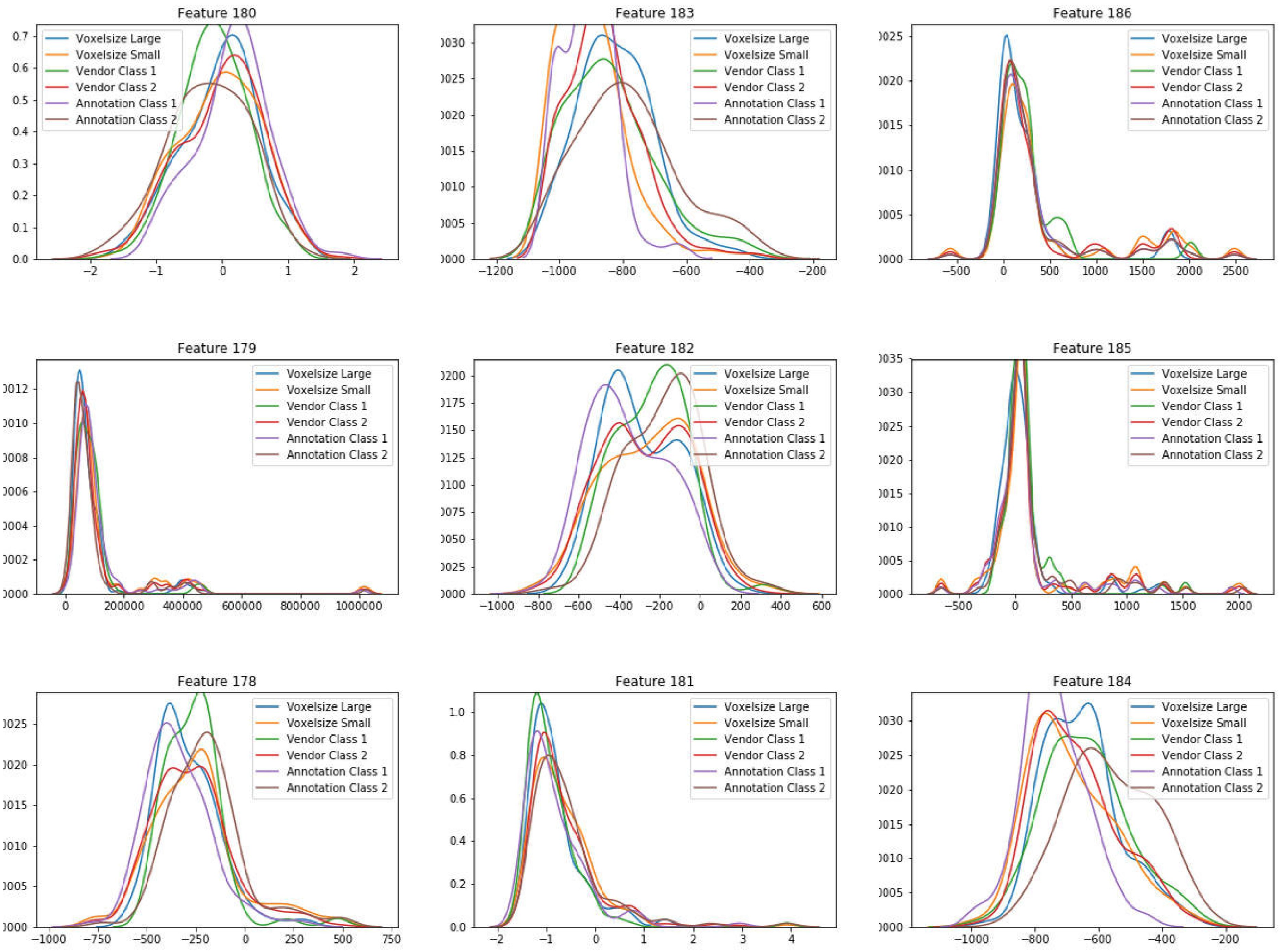
Feature 180
Feature 183
Feature 186
Feature 179
Feature 182
Feature 185
Feature 178
Feature 181
Feature 184
Voxelsize Large
Voxelsize Small
Vendor Class 1
Vendor Class 2
Annotation Class 1
Annotation Class 2

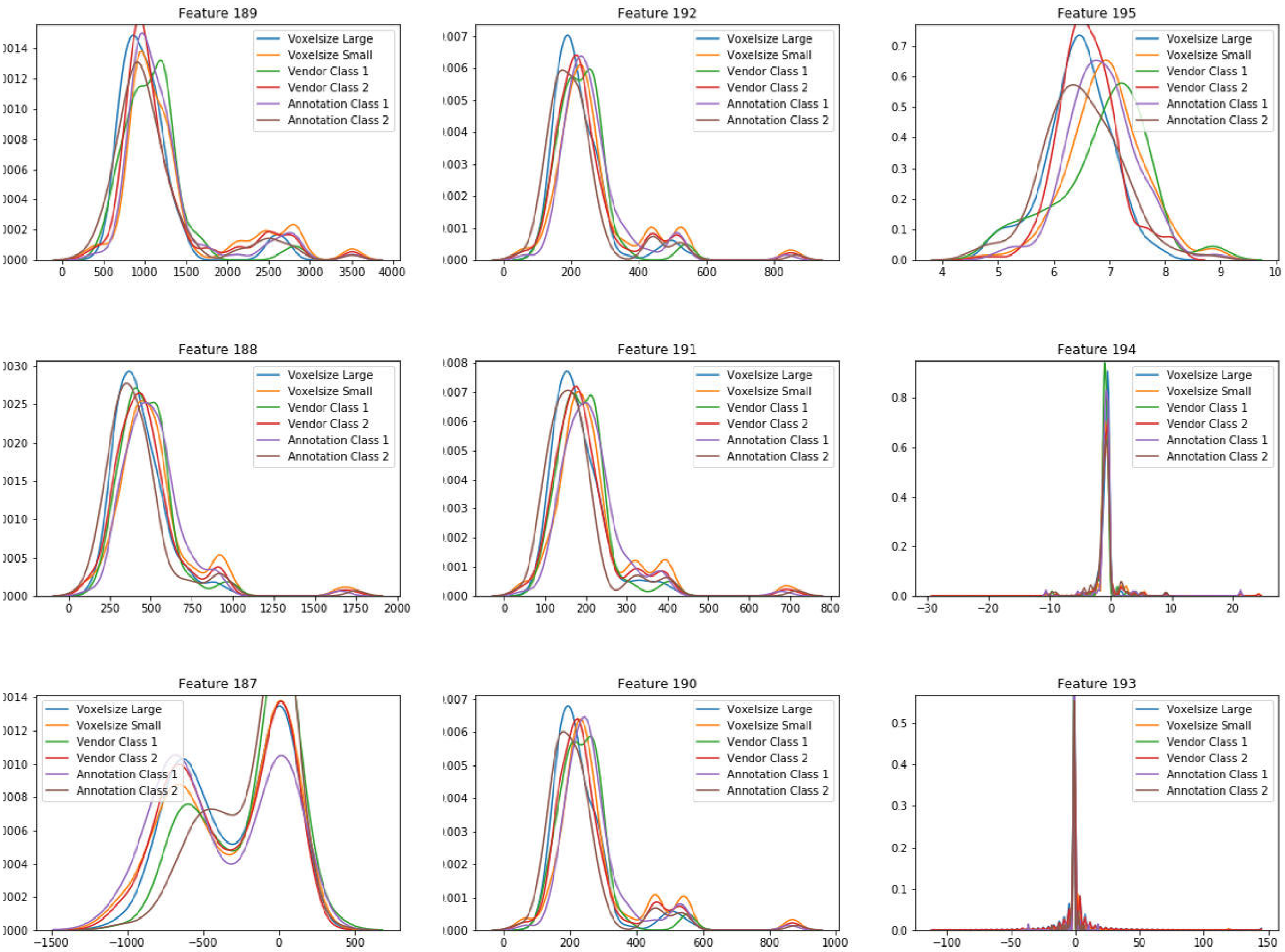
Feature 189
Feature 192
Feature 195
Feature 188
Feature 191
Feature 194
Feature 187
Feature 190
Feature 193
Voxelsize Large
Voxelsize Small
Vendor Class 1
Vendor Class 2
Annotation Class 1
Annotation Class 2

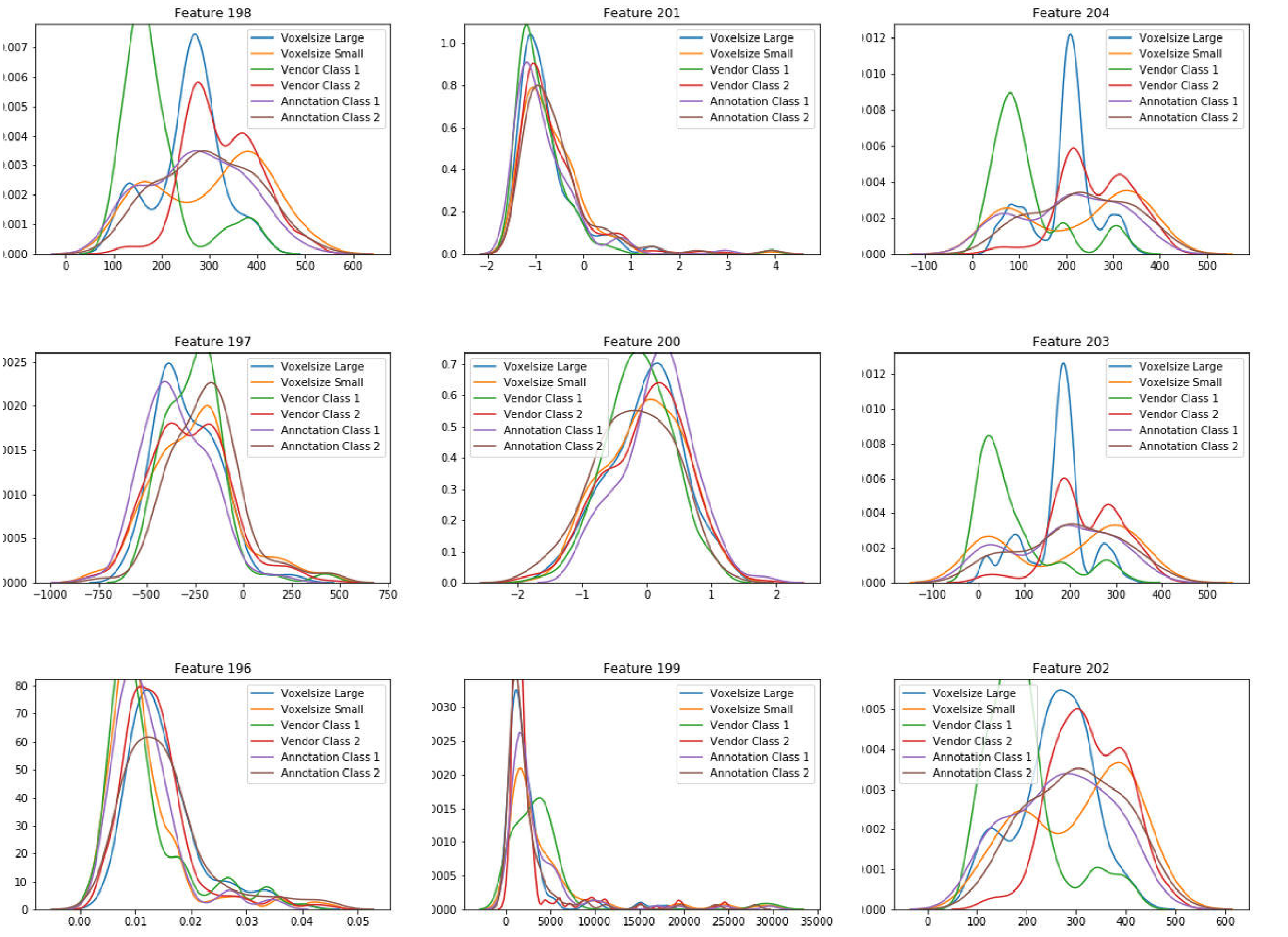
Feature 198
Feature 201
Feature 204
Feature 197
Feature 200
Feature 203
Feature 196
Feature 199
Feature 202
Voxelsize Large
Voxelsize Small
Vendor Class 1
Vendor Class 2
Annotation Class 1
Annotation Class 2

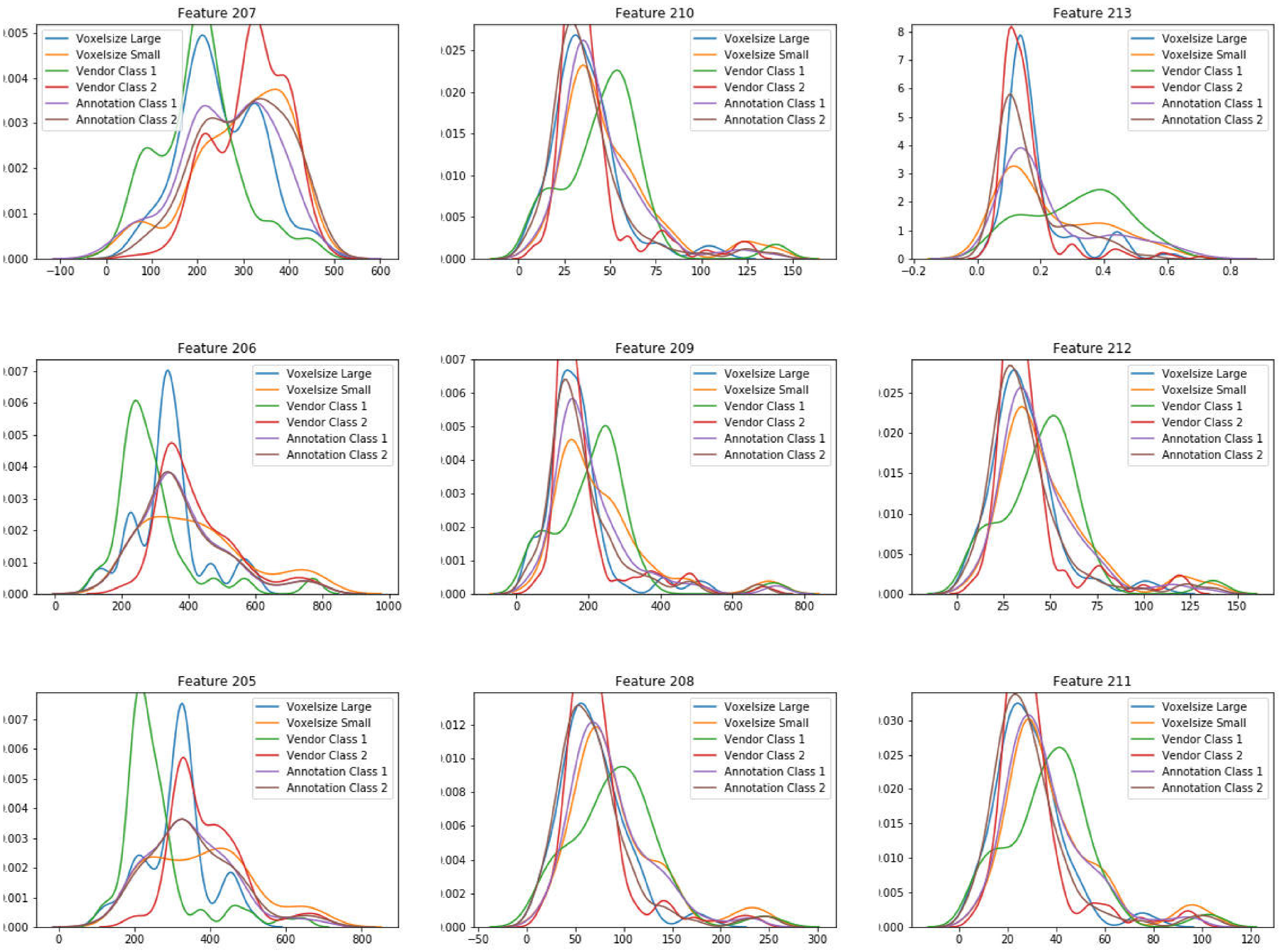
Feature 207
Feature 210
Feature 213
Feature 206
Feature 209
Feature 212
Feature 205
Feature 208
Feature 211
Voxelsize Large
Voxelsize Small
Vendor Class 1
Vendor Class 2
Annotation Class 1
Annotation Class 2

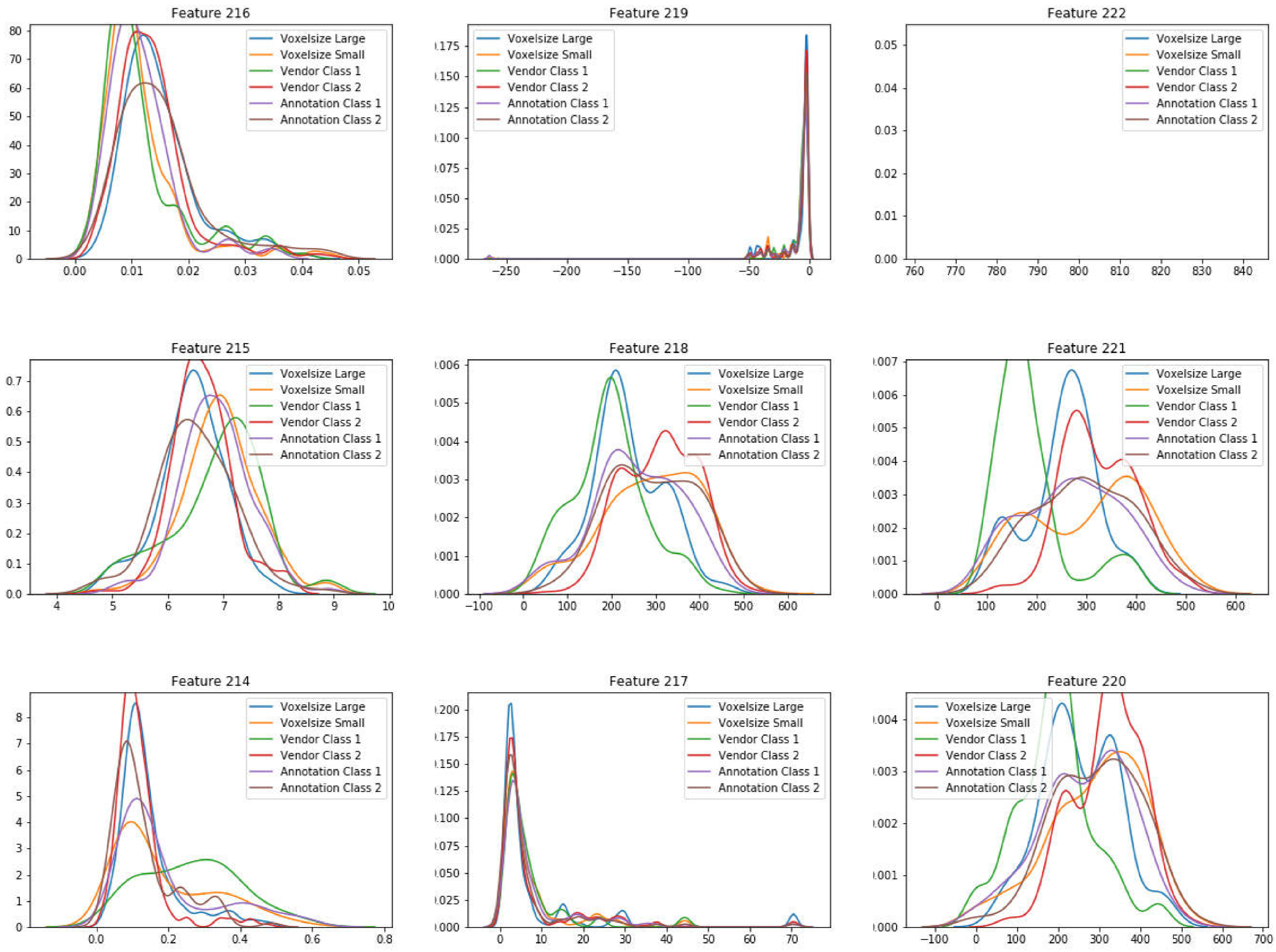
Feature 216
Feature 219
Feature 222
Feature 215
Feature 218
Feature 221
Feature 214
Feature 217
Feature 220
Voxelsize Large
Voxelsize Small
Vendor Class 1
Vendor Class 2
Annotation Class 1
Annotation Class 2

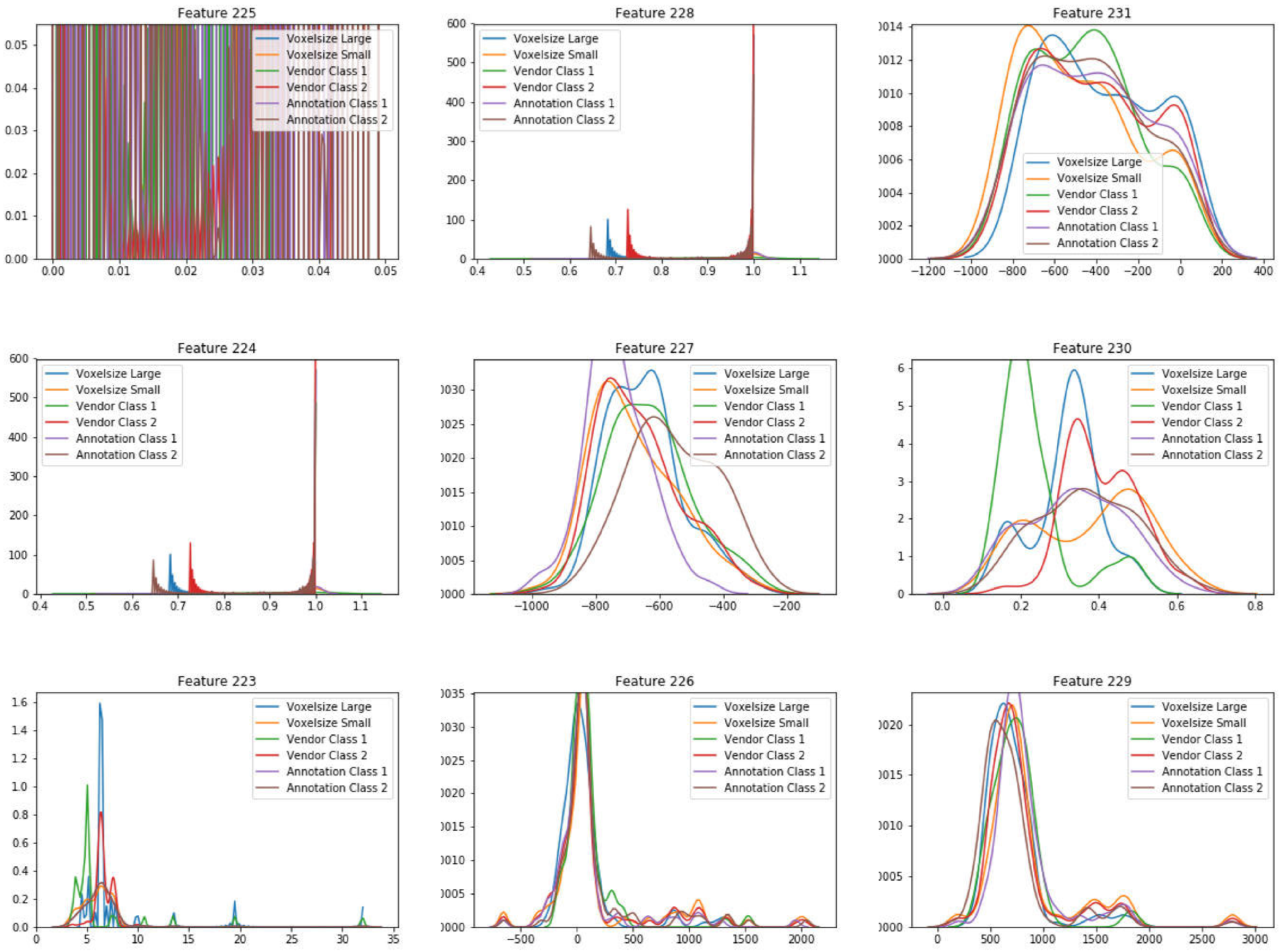
Feature 225
Feature 228
Feature 231
Feature 224
Feature 227
Feature 230
Feature 223
Feature 226
Feature 229
Voxelsize Large
Voxelsize Small
Vendor Class 1
Vendor Class 2
Annotation Class 1
Annotation Class 2

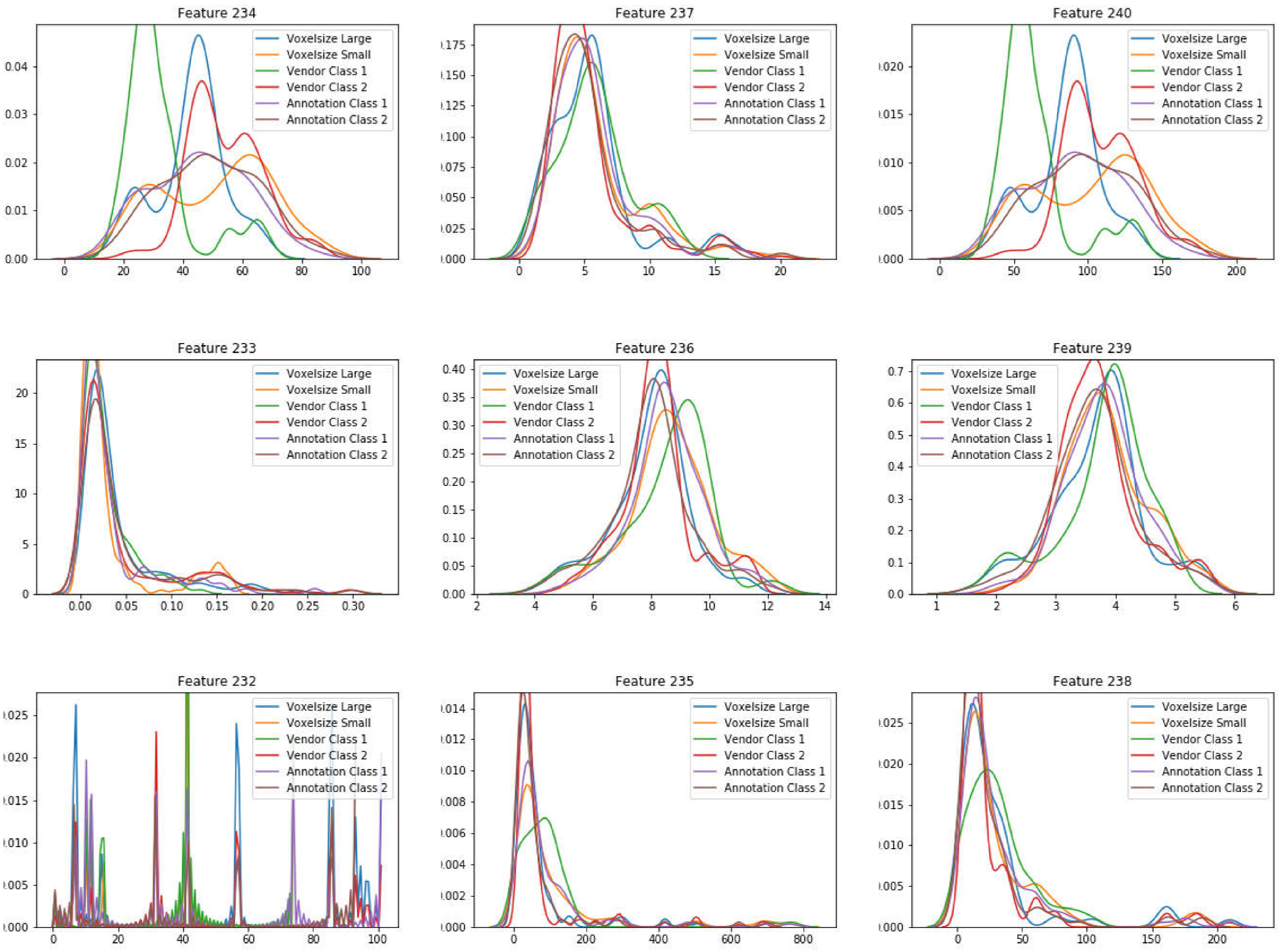
Feature 234
Feature 237
Feature 240
Feature 233
Feature 236
Feature 239
Feature 232
Feature 235
Feature 238
Voxelsize Large
Voxelsize Small
Vendor Class 1
Vendor Class 2
Annotation Class 1
Annotation Class 2

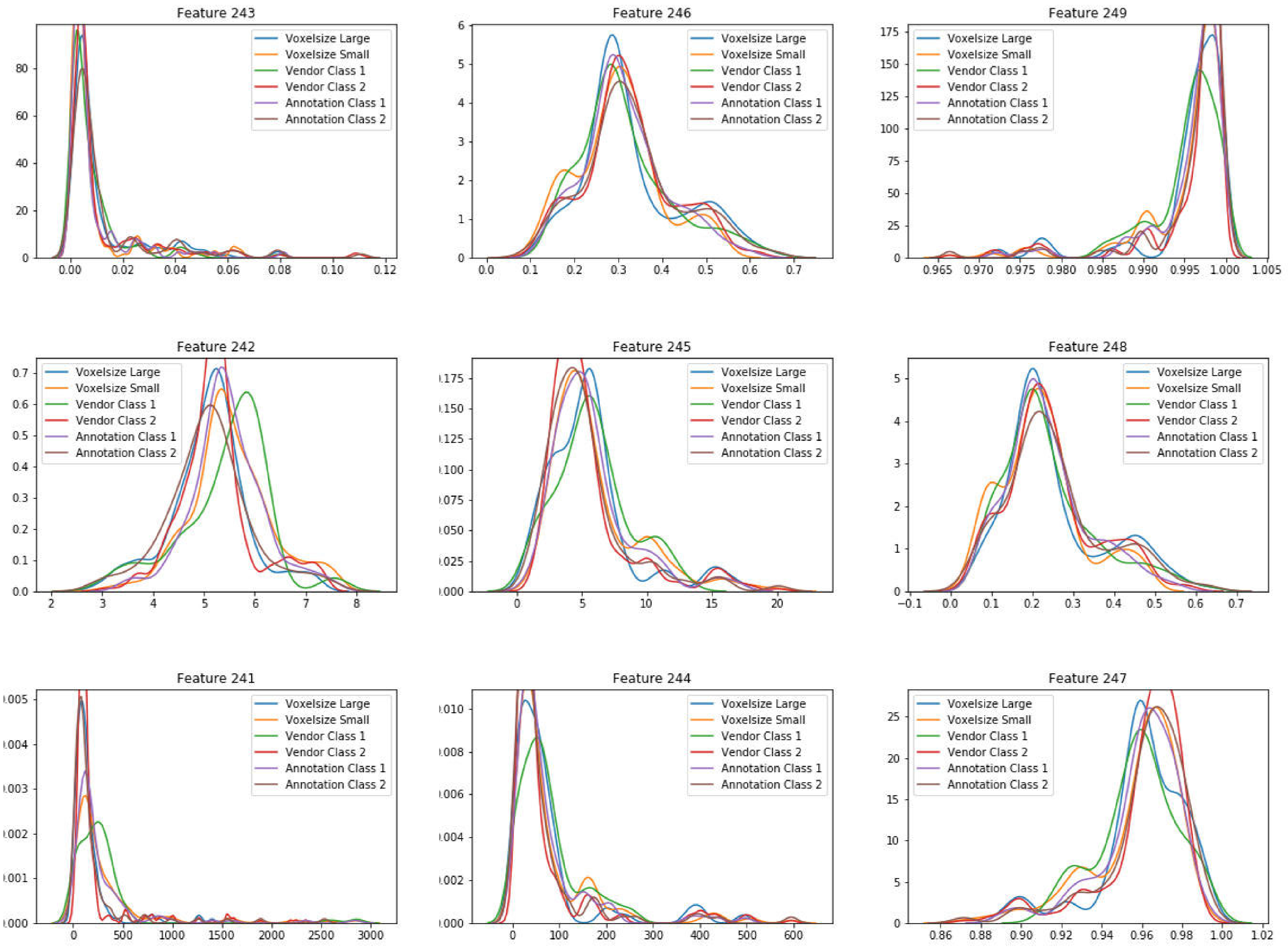
Feature 243
Feature 246
Feature 249
Feature 242
Feature 245
Feature 248
Feature 241
Feature 244
Feature 247
Voxelsize Large
Voxelsize Small
Vendor Class 1
Vendor Class 2
Annotation Class 1
Annotation Class 2

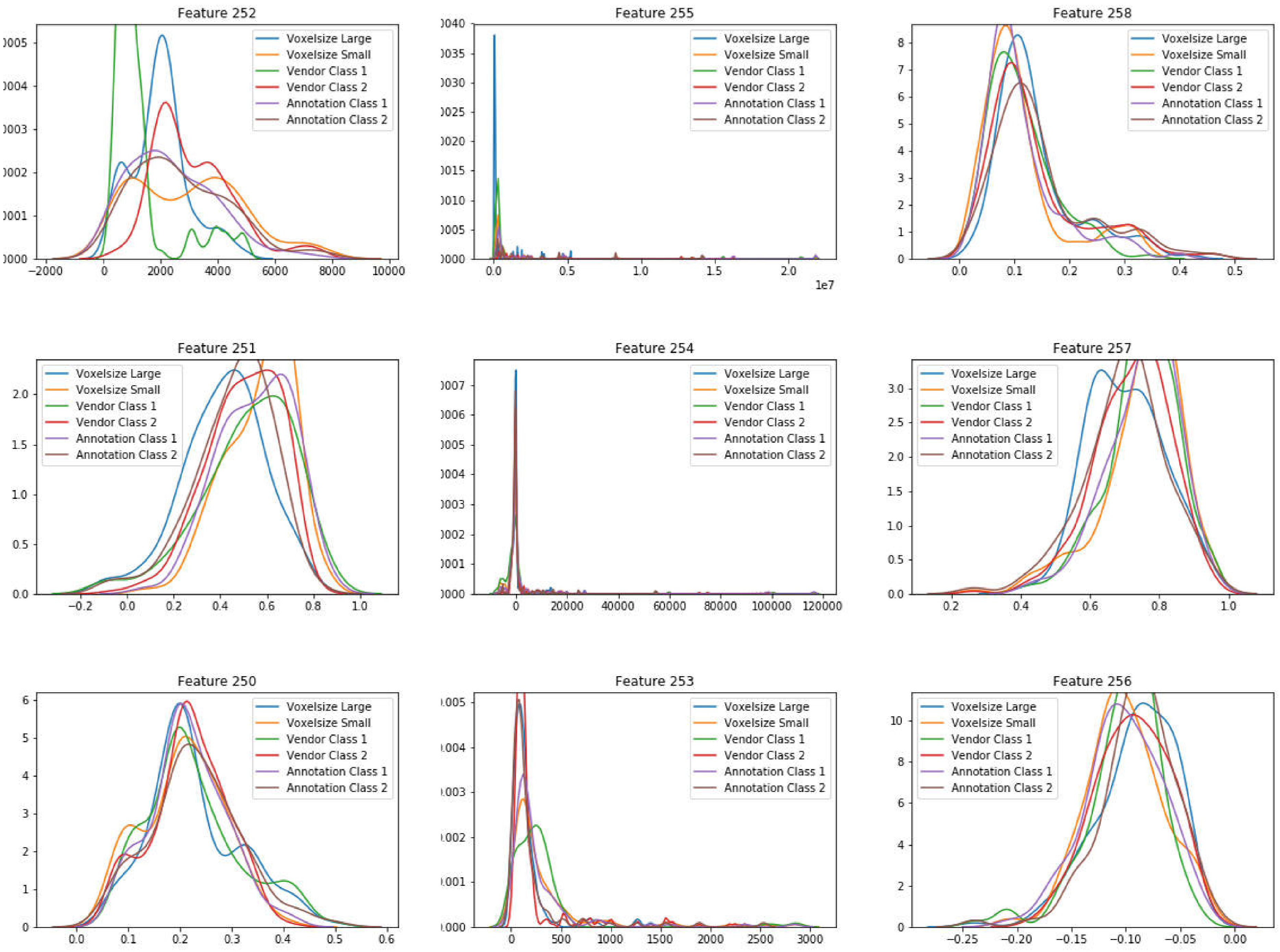
Feature 252
Feature 255
Feature 258
Feature 251
Feature 254
Feature 257
Feature 250
Feature 253
Feature 256
Voxelsize Large
Voxelsize Small
Vendor Class 1
Vendor Class 2
Annotation Class 1
Annotation Class 2
1e7

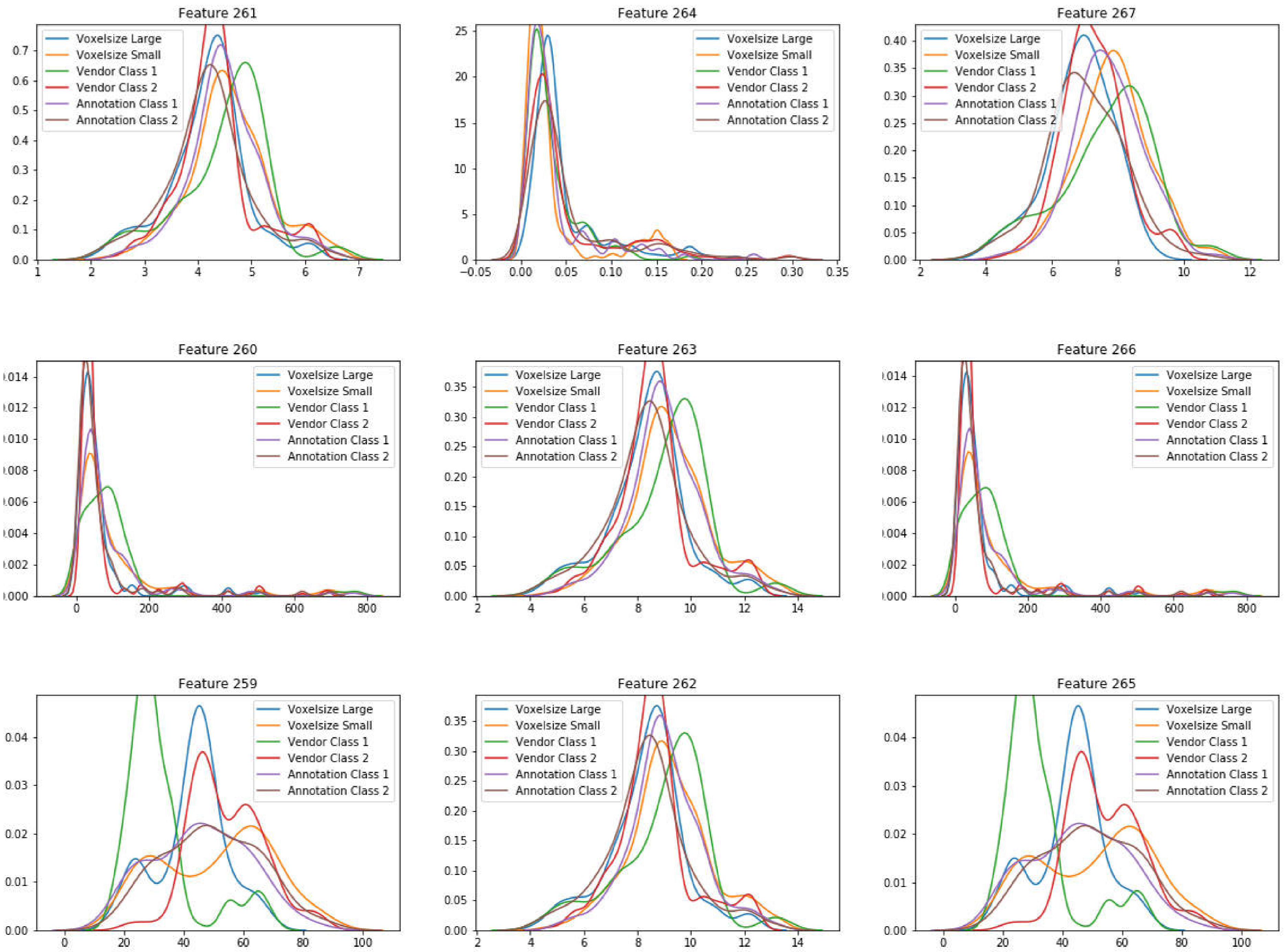
Feature 261
Feature 264
Feature 267
Feature 260
Feature 263
Feature 266
Feature 259
Feature 262
Feature 265
Voxelsize Large
Voxelsize Small
Vendor Class 1
Vendor Class 2
Annotation Class 1
Annotation Class 2

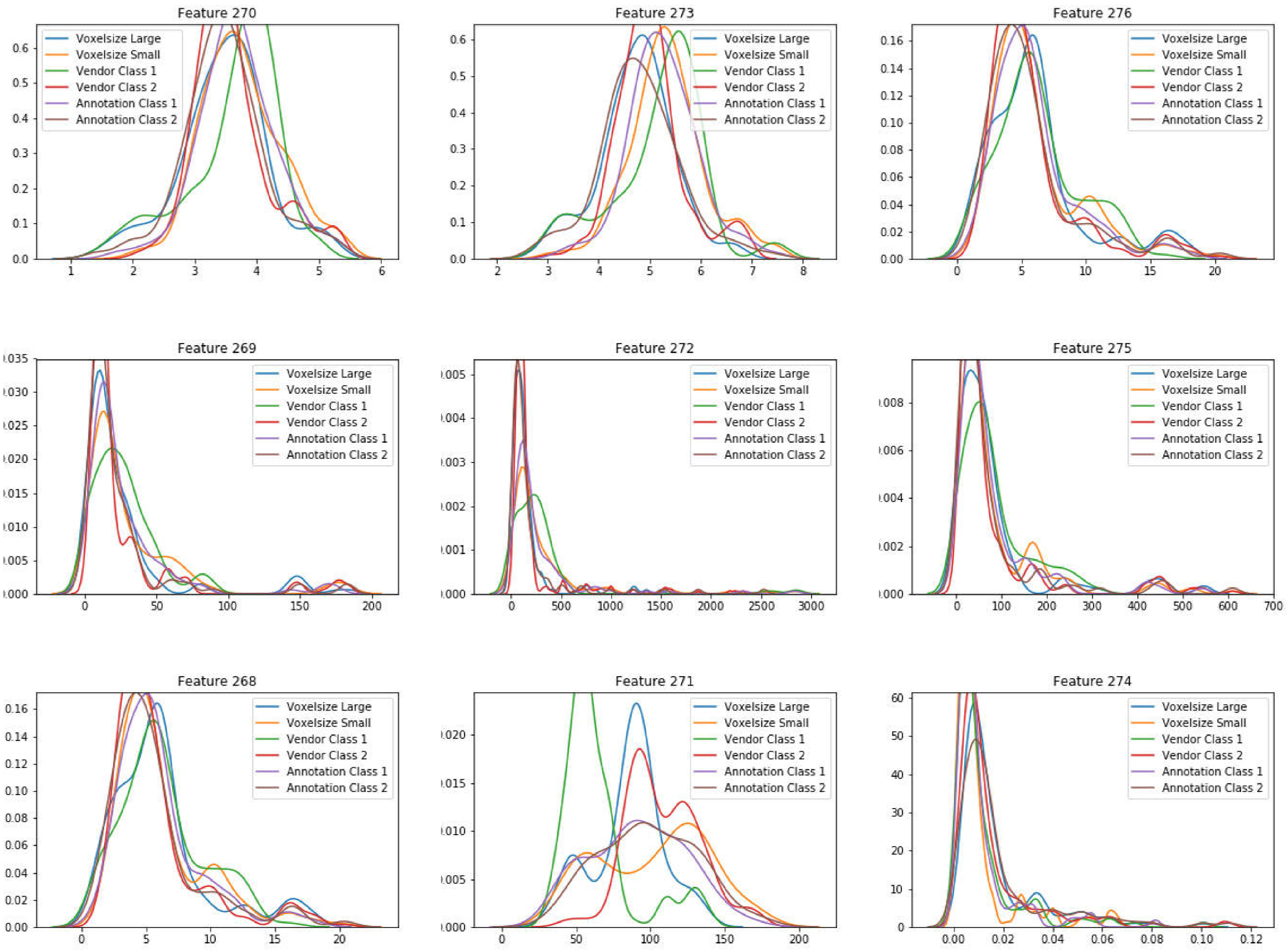
Feature 270
Feature 273
Feature 276
Feature 269
Feature 272
Feature 275
Feature 268
Feature 271
Feature 274
Voxelsize Large
Voxelsize Small
Vendor Class 1
Vendor Class 2
Annotation Class 1
Annotation Class 2

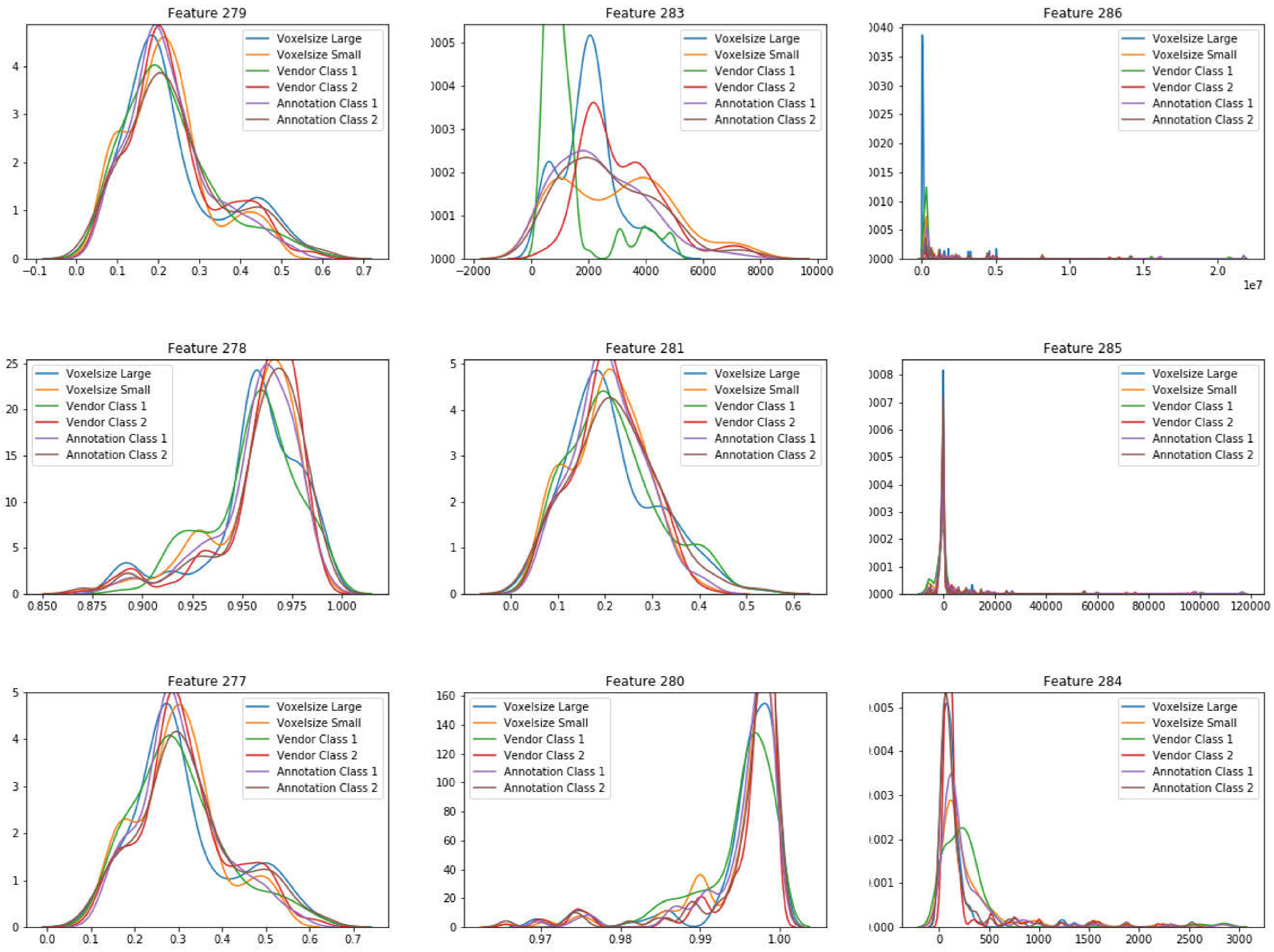
Feature 279
Feature 283
Feature 286
Feature 278
Feature 281
Feature 285
Feature 277
Feature 280
Feature 284
Voxelsize Large
Voxelsize Small
Vendor Class 1
Vendor Class 2
Annotation Class 1
Annotation Class 2
1e7

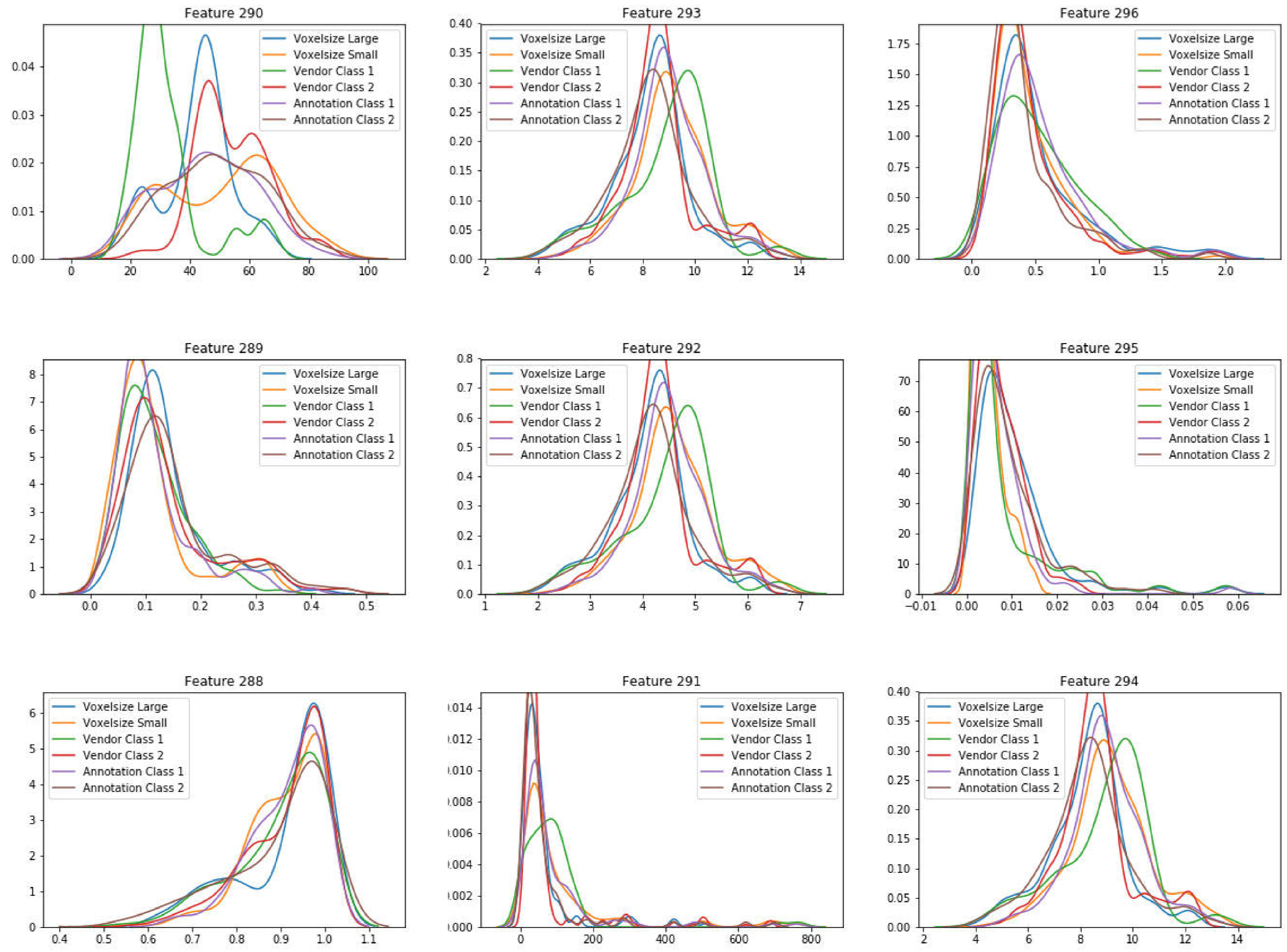
Feature 290
Feature 293
Feature 296
Feature 289
Feature 292
Feature 295
Feature 288
Feature 291
Feature 294
Voxelsize Large
Voxelsize Small
Vendor Class 1
Vendor Class 2
Annotation Class 1
Annotation Class 2

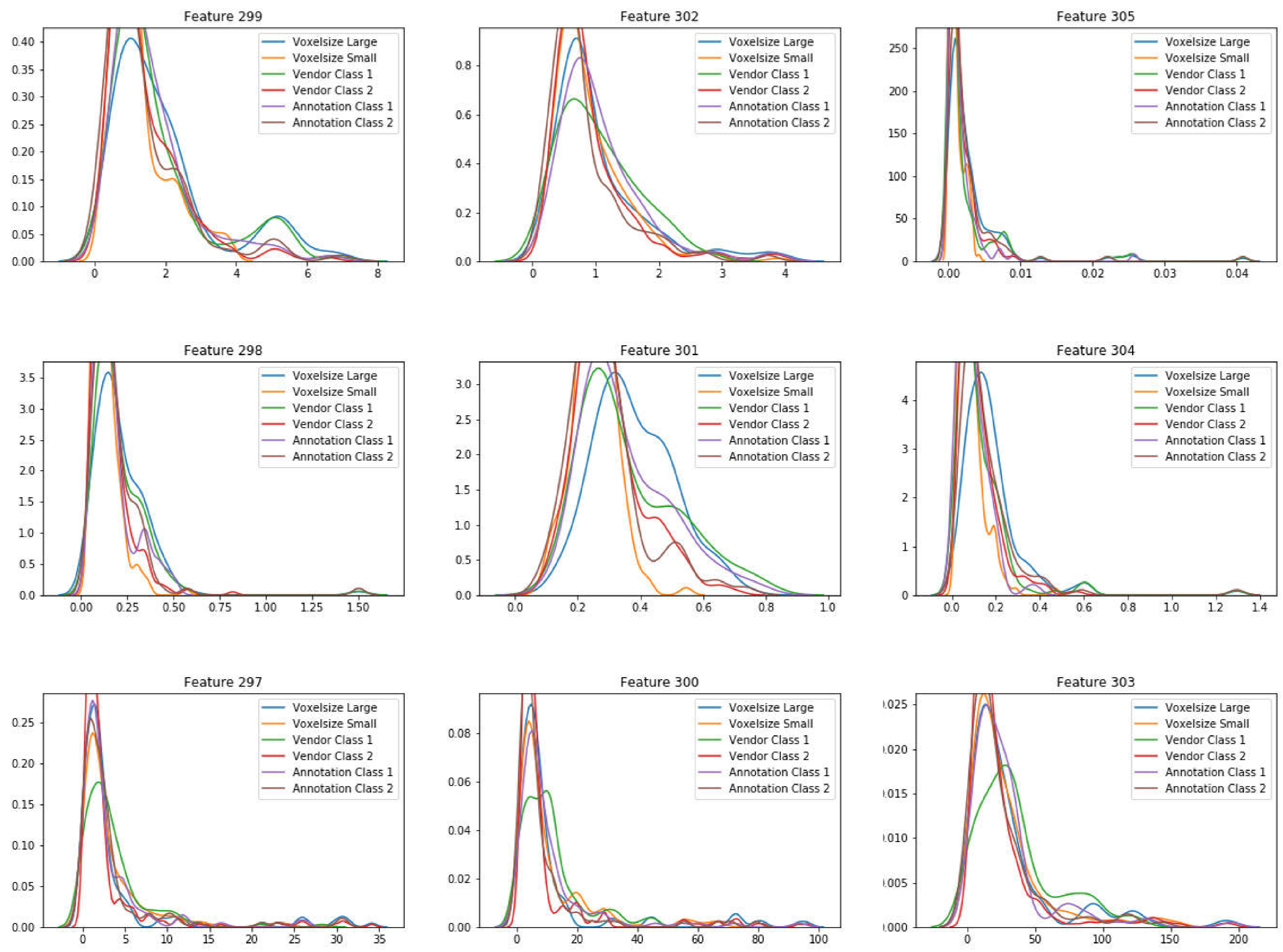
Feature 299
Feature 302
Feature 305
Feature 298
Feature 301
Feature 304
Feature 297
Feature 300
Feature 303
Voxelsize Large
Voxelsize Small
Vendor Class 1
Vendor Class 2
Annotation Class 1
Annotation Class 2

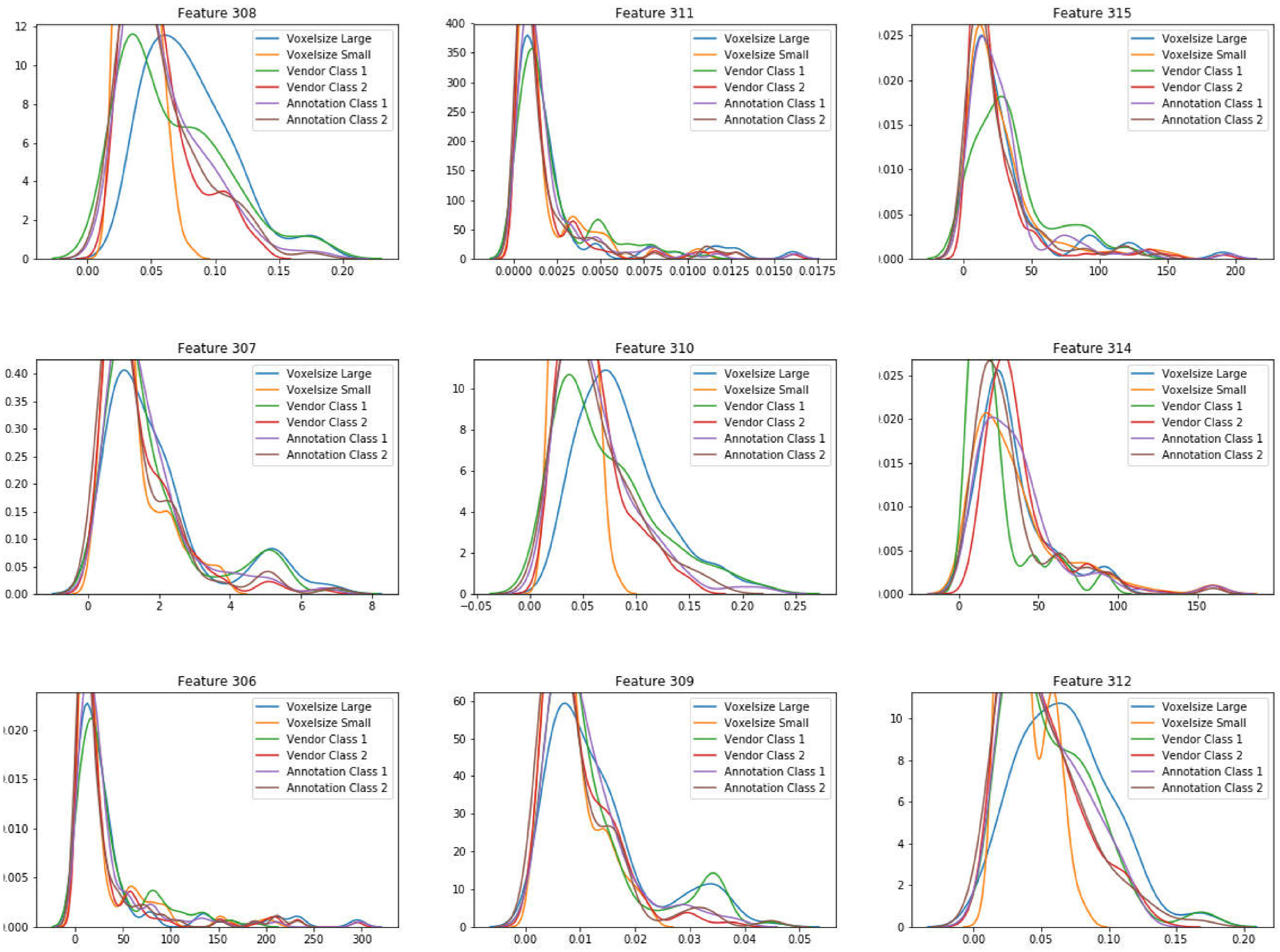
Feature 308
Feature 311
Feature 315
Feature 307
Feature 310
Feature 314
Feature 306
Feature 309
Feature 312
Voxelsize Large
Voxelsize Small
Vendor Class 1
Vendor Class 2
Annotation Class 1
Annotation Class 2

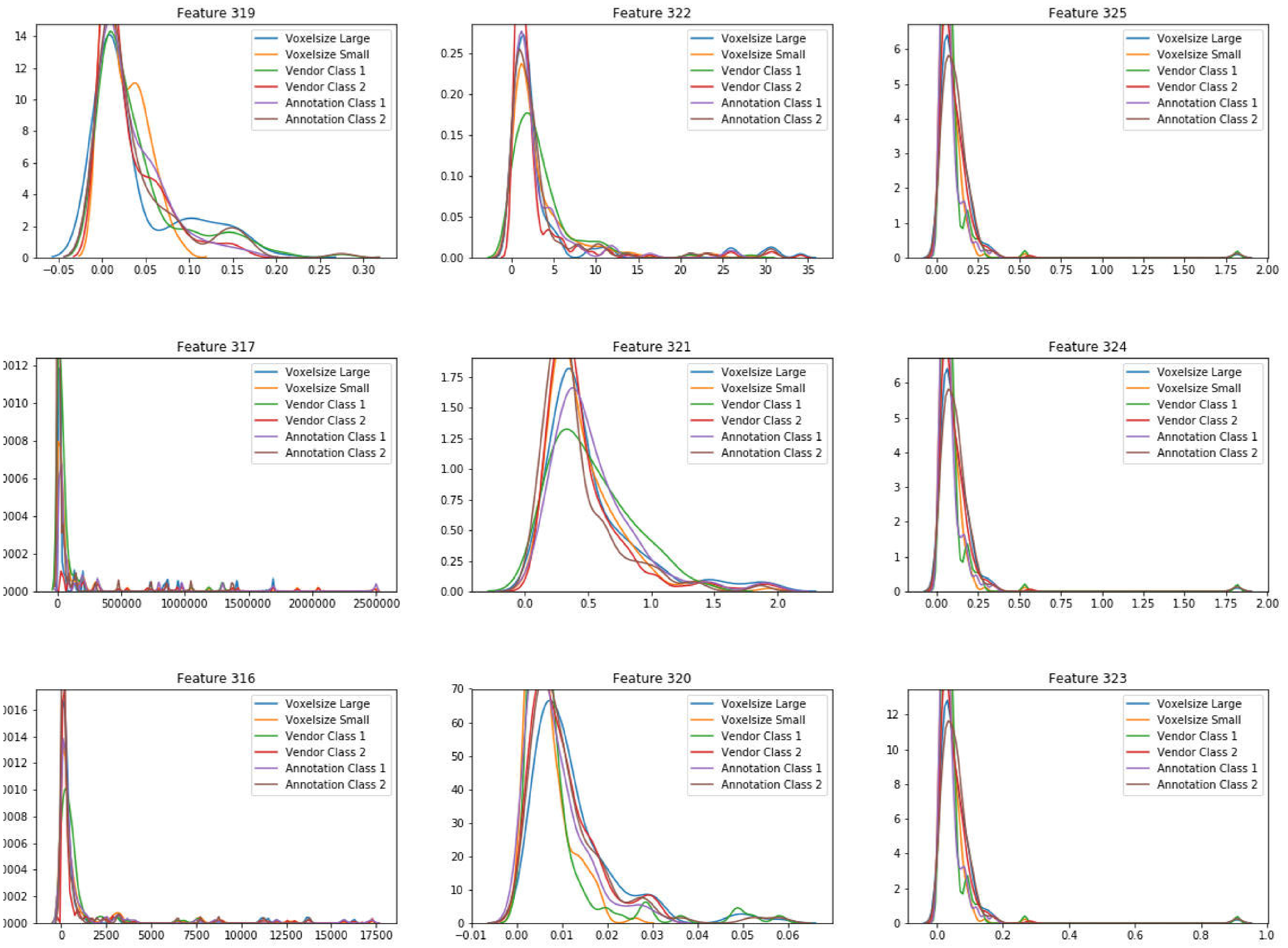
Feature 319
Feature 322
Feature 325
Feature 317
Feature 321
Feature 324
Feature 316
Feature 320
Feature 323
Voxelsize Large
Voxelsize Small
Vendor Class 1
Vendor Class 2
Annotation Class 1
Annotation Class 2

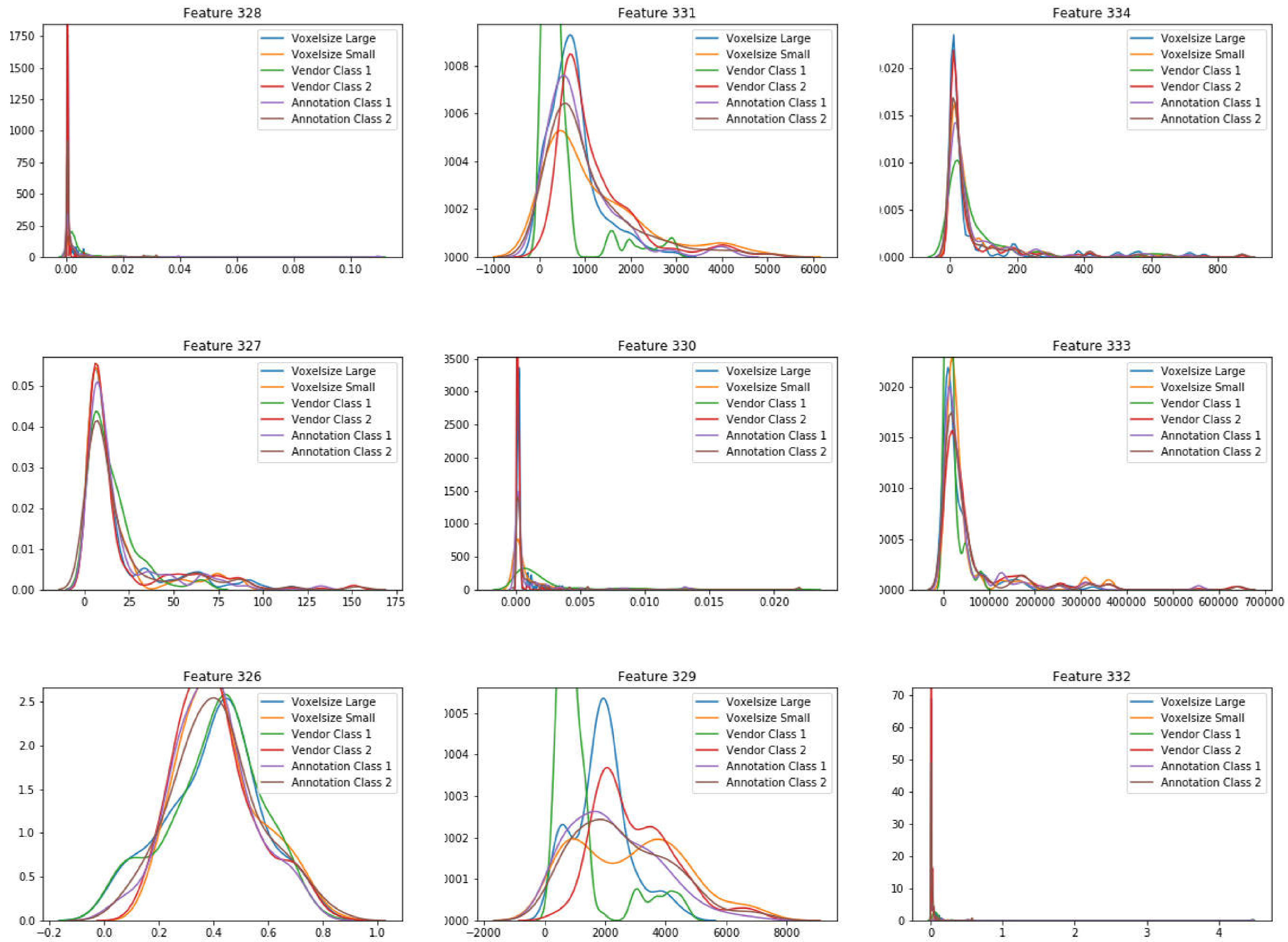
Feature 328
Feature 331
Feature 334
Feature 327
Feature 330
Feature 333
Feature 326
Feature 329
Feature 332
Voxelsize Large
Voxelsize Small
Vendor Class 1
Vendor Class 2
Annotation Class 1
Annotation Class 2

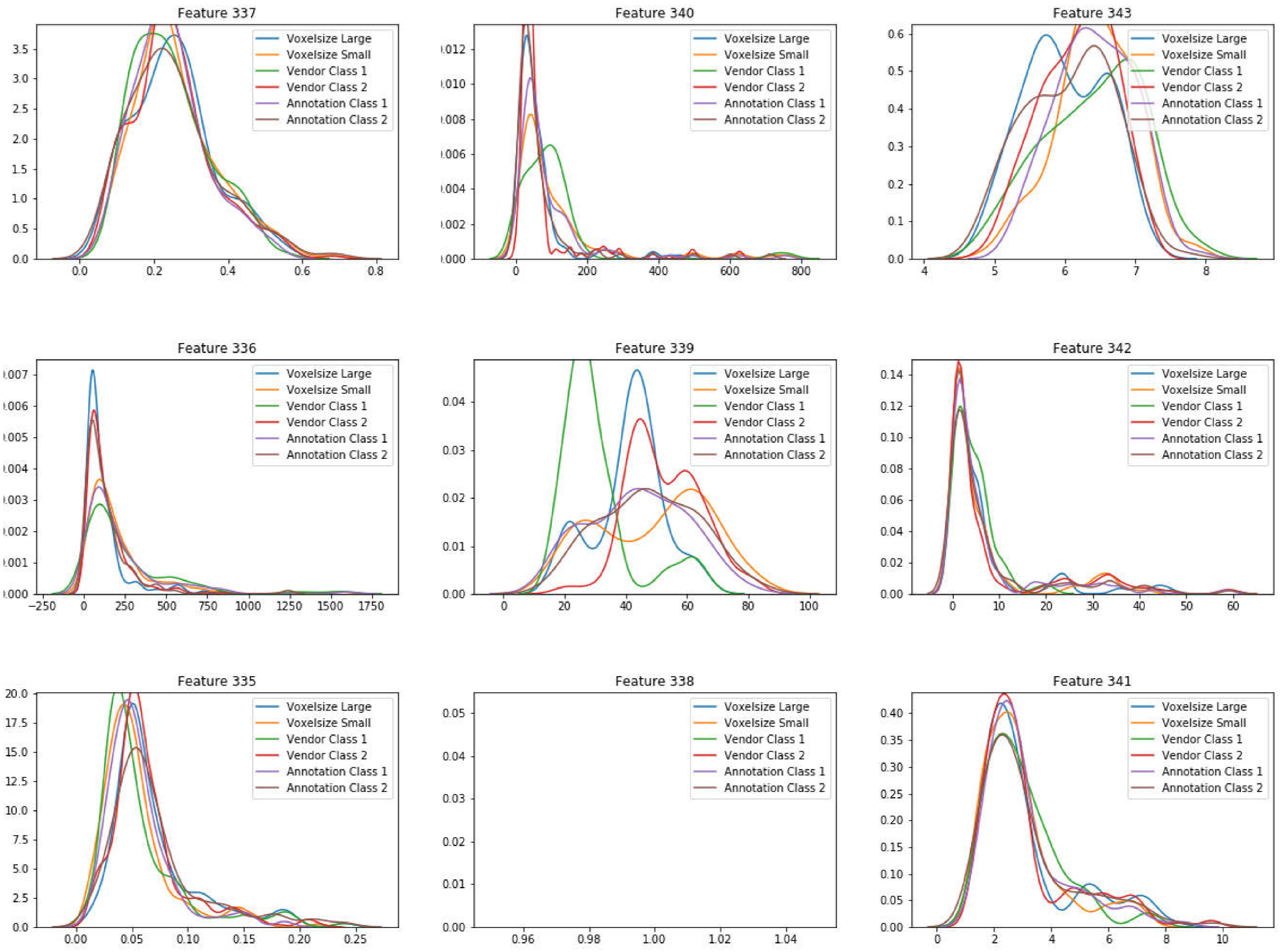
Feature 337
Feature 340
Feature 343
Feature 336
Feature 339
Feature 342
Feature 335
Feature 338
Feature 341
Voxelsize Large
Voxelsize Small
Vendor Class 1
Vendor Class 2
Annotation Class 1
Annotation Class 2

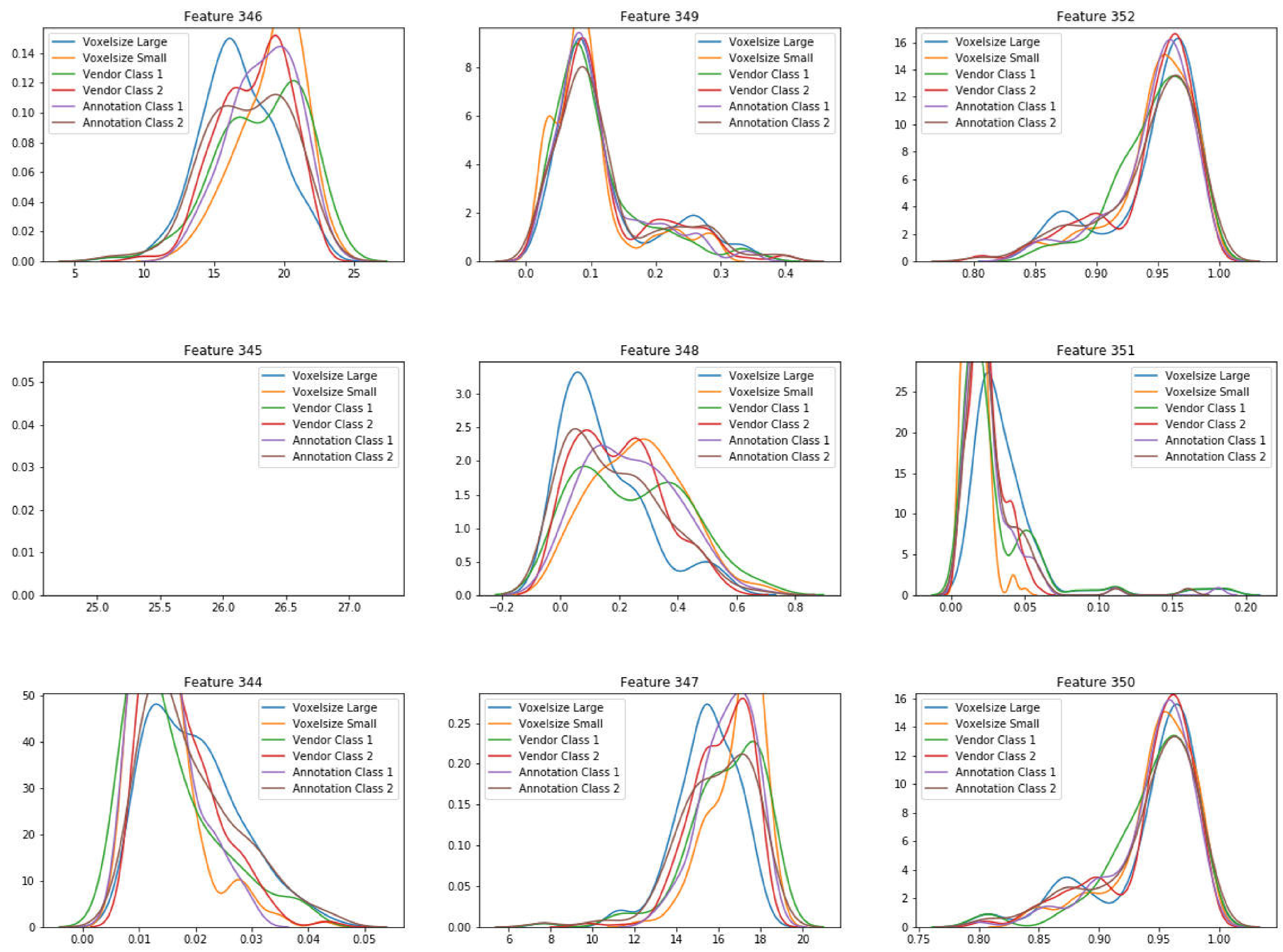
Feature 346
Feature 349
Feature 352
Feature 345
Feature 348
Feature 351
Feature 344
Feature 347
Feature 350
Voxelsize Large
Voxelsize Small
Vendor Class 1
Vendor Class 2
Annotation Class 1
Annotation Class 2

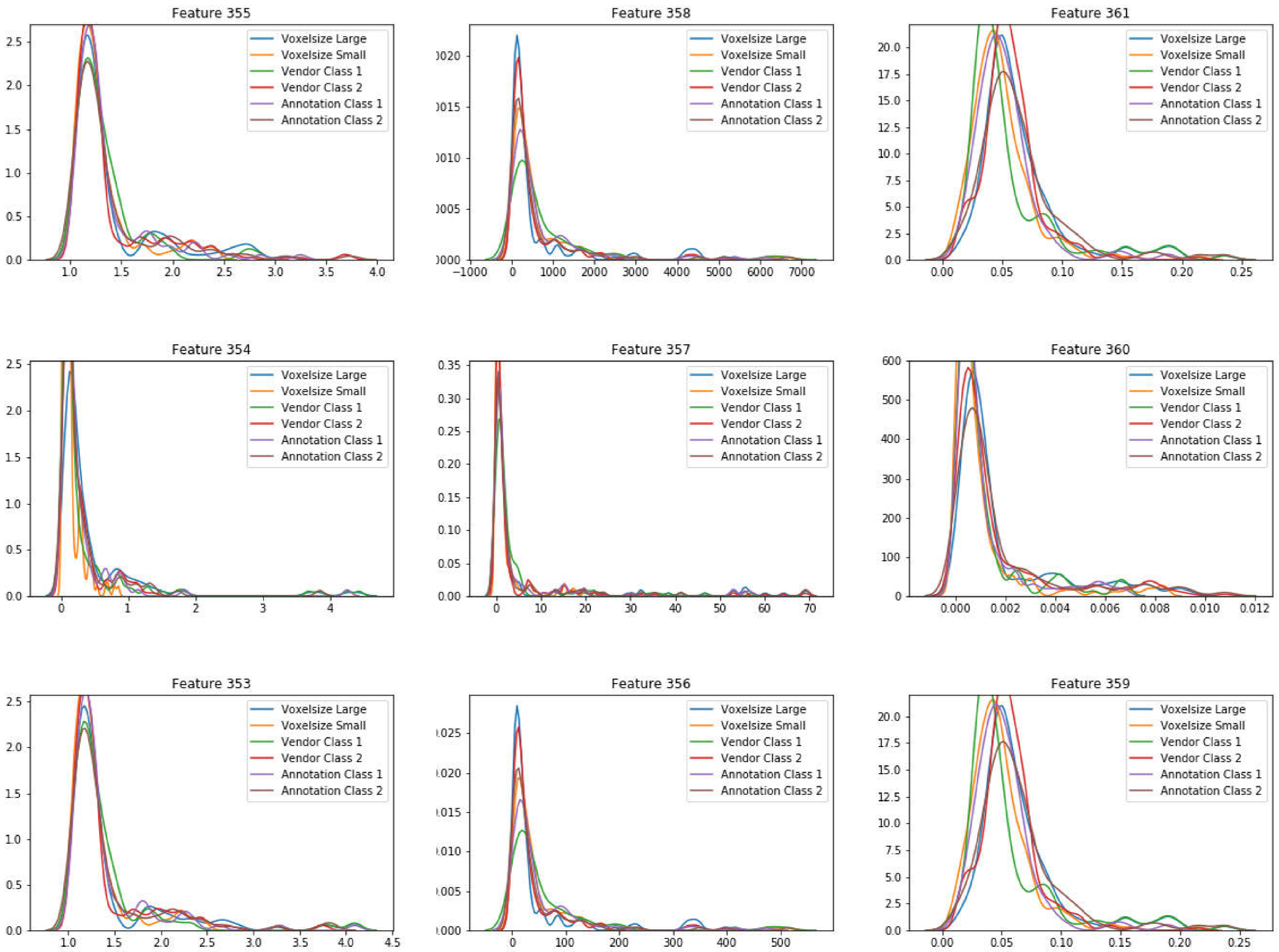
Feature 355
Feature 358
Feature 361
Feature 354
Feature 357
Feature 360
Feature 353
Feature 356
Feature 359
Voxelsize Large
Voxelsize Small
Vendor Class 1
Vendor Class 2
Annotation Class 1
Annotation Class 2

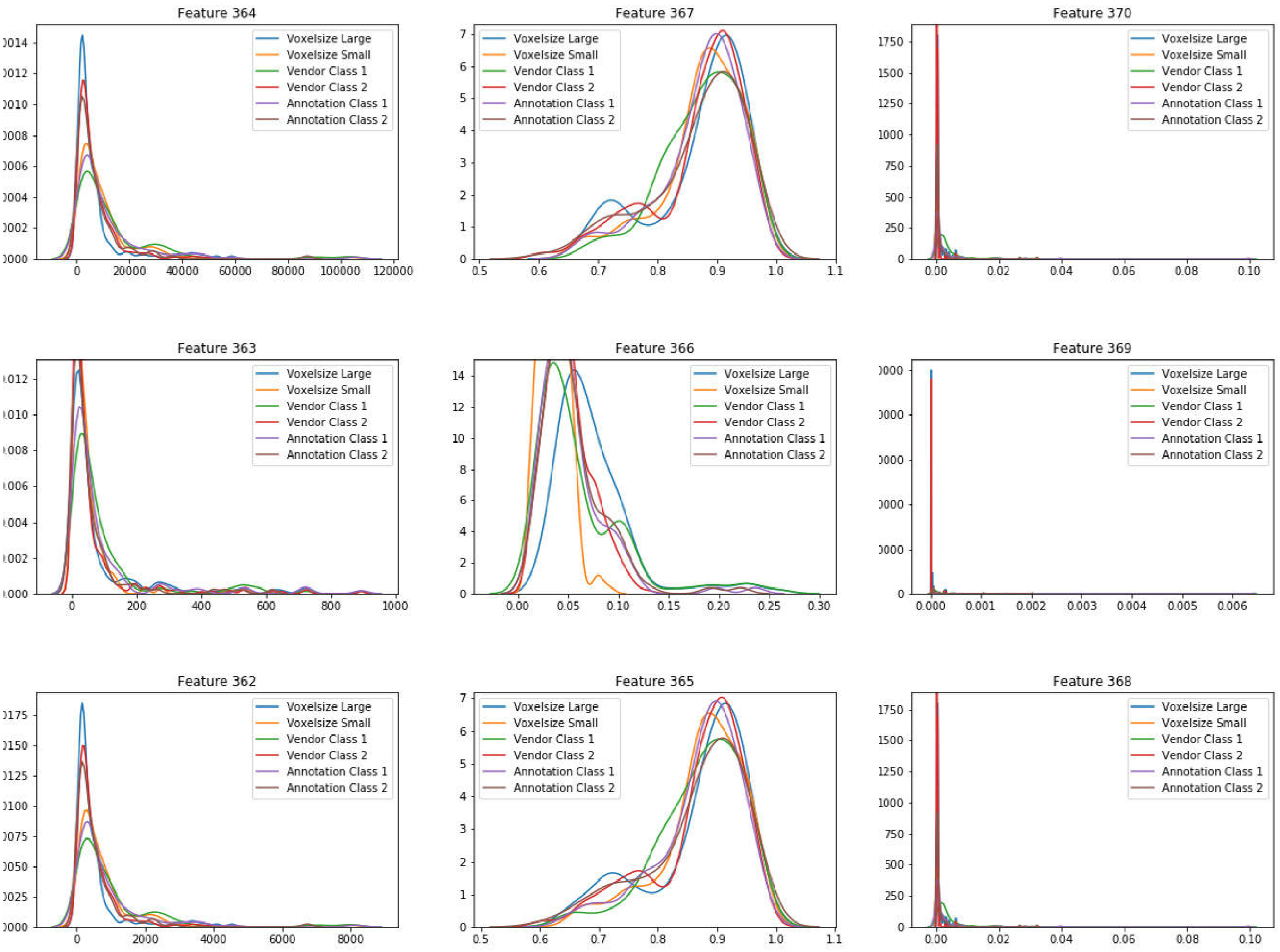
Feature 364
Feature 367
Feature 370
Feature 363
Feature 366
Feature 369
Feature 362
Feature 365
Feature 368
Voxelsize Large
Voxelsize Small
Vendor Class 1
Vendor Class 2
Annotation Class 1
Annotation Class 2

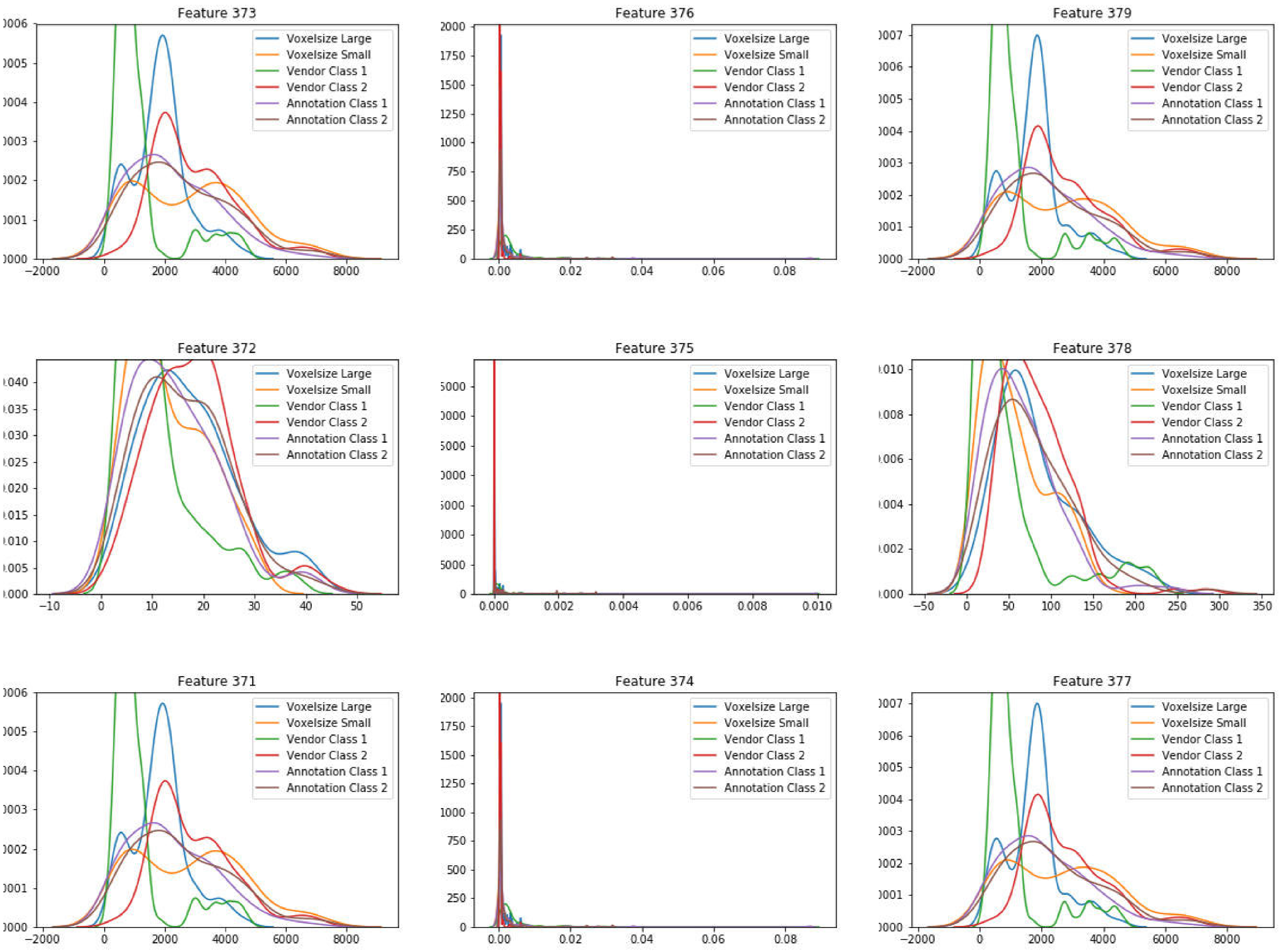
Feature 373
Feature 376
Feature 379
Feature 372
Feature 375
Feature 378
Feature 371
Feature 374
Feature 377
Voxelsize Large
Voxelsize Small
Vendor Class 1
Vendor Class 2
Annotation Class 1
Annotation Class 2

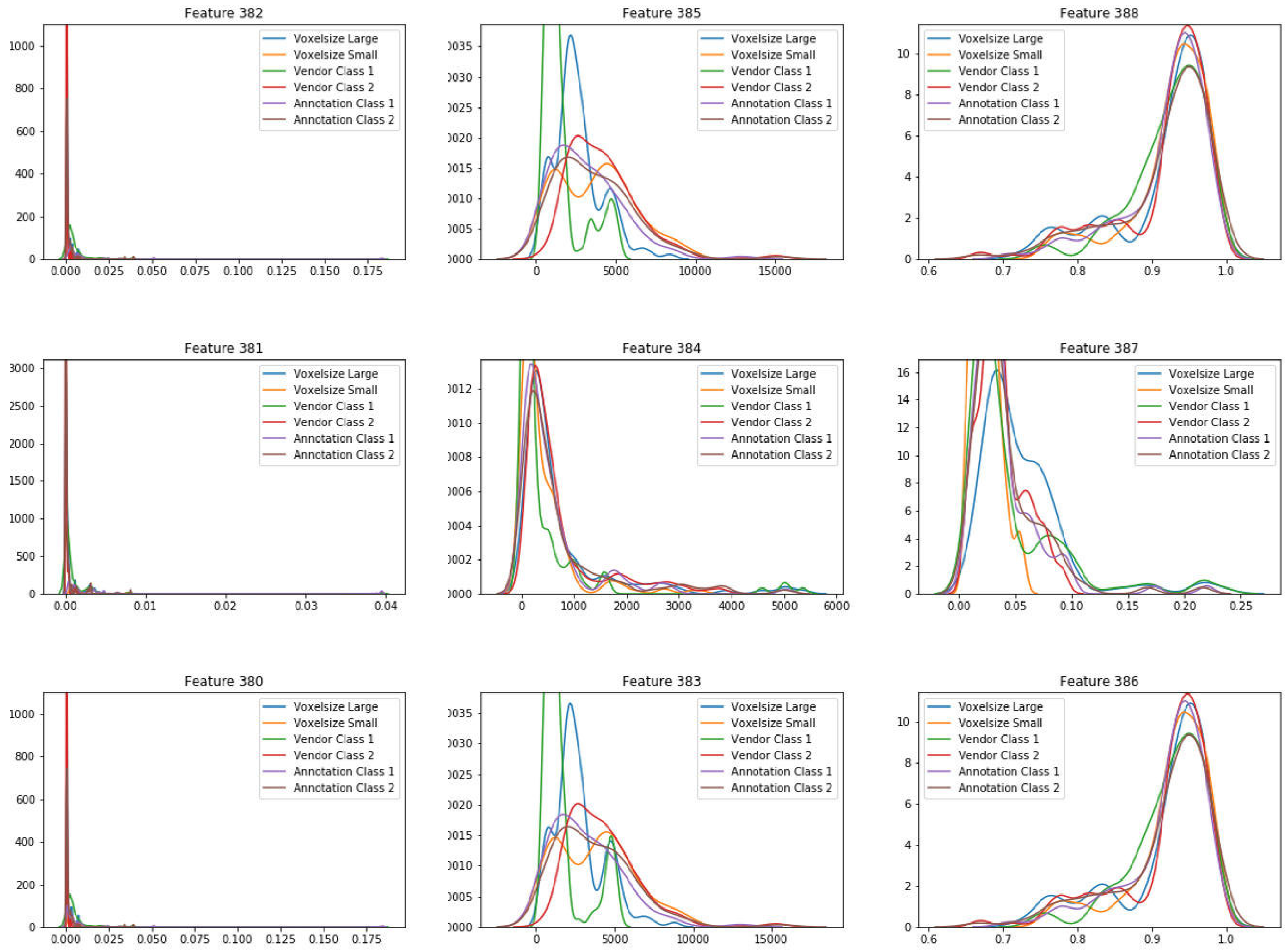
Feature 382
Feature 385
Feature 388
Feature 381
Feature 384
Feature 387
Feature 380
Feature 383
Feature 386
Voxelsize Large
Voxelsize Small
Vendor Class 1
Vendor Class 2
Annotation Class 1
Annotation Class 2

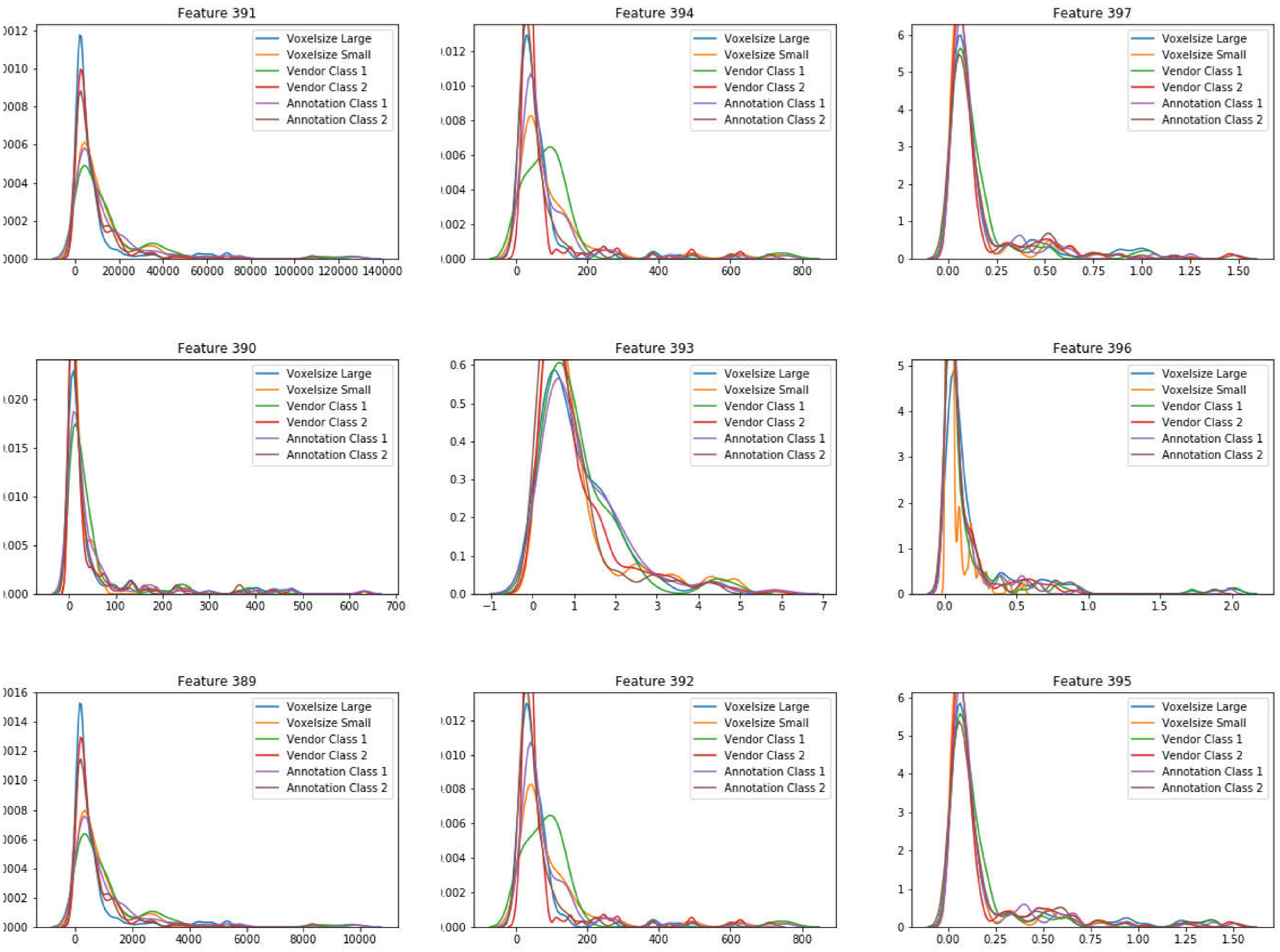
Feature 391
Voxelsize Large
Voxelsize Small
Vendor Class 1
Vendor Class 2
Annotation Class 1
Annotation Class 2
Feature 394
Voxelsize Large
Voxelsize Small
Vendor Class 1
Vendor Class 2
Annotation Class 1
Annotation Class 2
Feature 397
Voxelsize Large
Voxelsize Small
Vendor Class 1
Vendor Class 2
Annotation Class 1
Annotation Class 2
Feature 390
Voxelsize Large
Voxelsize Small
Vendor Class 1
Vendor Class 2
Annotation Class 1
Annotation Class 2
Feature 393
Voxelsize Large
Voxelsize Small
Vendor Class 1
Vendor Class 2
Annotation Class 1
Annotation Class 2
Feature 396
Voxelsize Large
Voxelsize Small
Vendor Class 1
Vendor Class 2
Annotation Class 1
Annotation Class 2
Feature 389
Voxelsize Large
Voxelsize Small
Vendor Class 1
Vendor Class 2
Annotation Class 1
Annotation Class 2
Feature 392
Voxelsize Large
Voxelsize Small
Vendor Class 1
Vendor Class 2
Annotation Class 1
Annotation Class 2
Feature 395
Voxelsize Large
Voxelsize Small
Vendor Class 1
Vendor Class 2
Annotation Class 1
Annotation Class 2

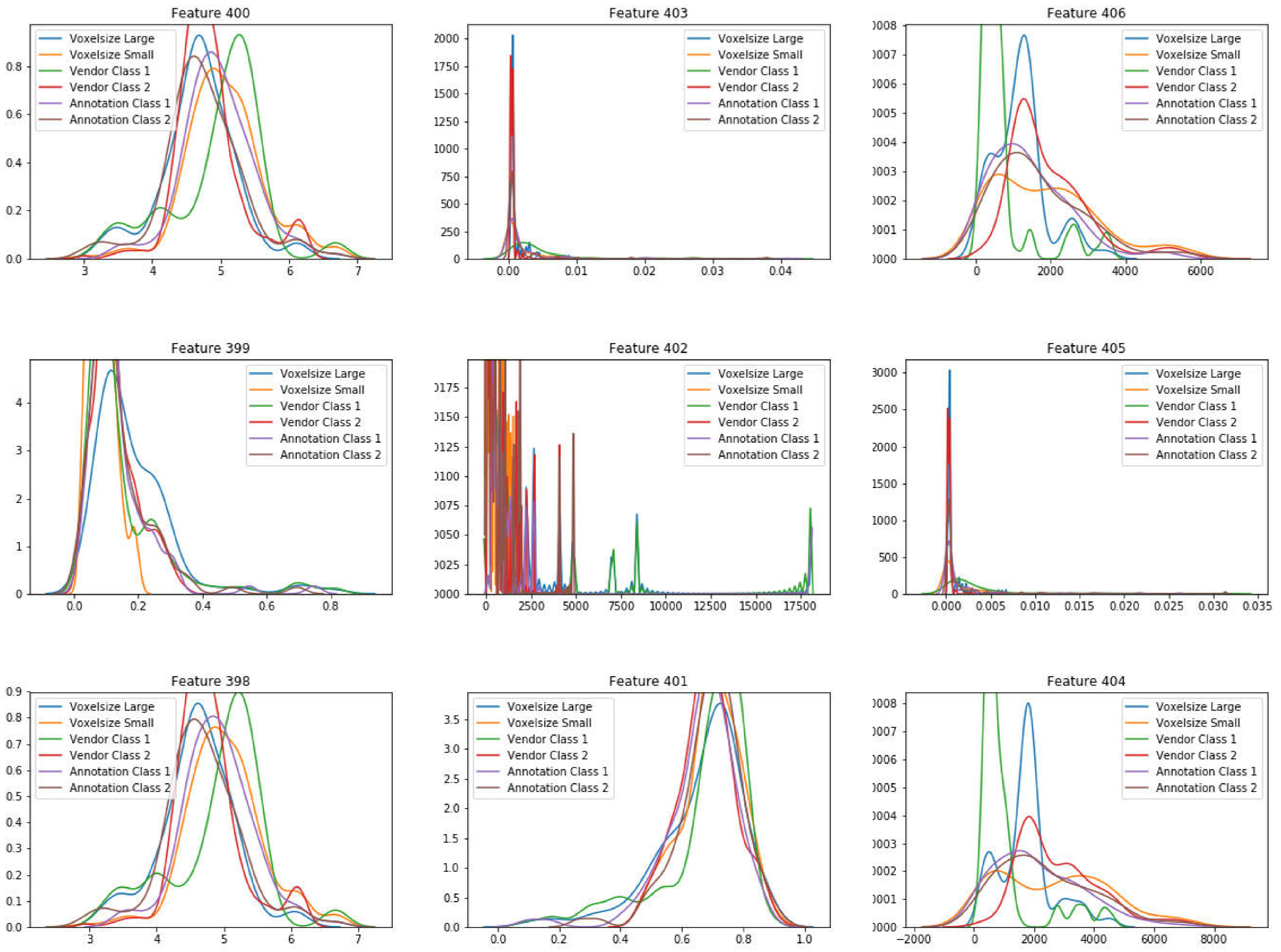
Feature 400
Voxelsize Large
Voxelsize Small
Vendor Class 1
Vendor Class 2
Annotation Class 1
Annotation Class 2
Feature 403
Voxelsize Large
Voxelsize Small
Vendor Class 1
Vendor Class 2
Annotation Class 1
Annotation Class 2
Feature 406
Voxelsize Large
Voxelsize Small
Vendor Class 1
Vendor Class 2
Annotation Class 1
Annotation Class 2
Feature 399
Voxelsize Large
Voxelsize Small
Vendor Class 1
Vendor Class 2
Annotation Class 1
Annotation Class 2
Feature 402
Voxelsize Large
Voxelsize Small
Vendor Class 1
Vendor Class 2
Annotation Class 1
Annotation Class 2
Feature 405
Voxelsize Large
Voxelsize Small
Vendor Class 1
Vendor Class 2
Annotation Class 1
Annotation Class 2
Feature 398
Voxelsize Large
Voxelsize Small
Vendor Class 1
Vendor Class 2
Annotation Class 1
Annotation Class 2
Feature 401
Voxelsize Large
Voxelsize Small
Vendor Class 1
Vendor Class 2
Annotation Class 1
Annotation Class 2
Feature 404
Voxelsize Large
Voxelsize Small
Vendor Class 1
Vendor Class 2
Annotation Class 1
Annotation Class 2

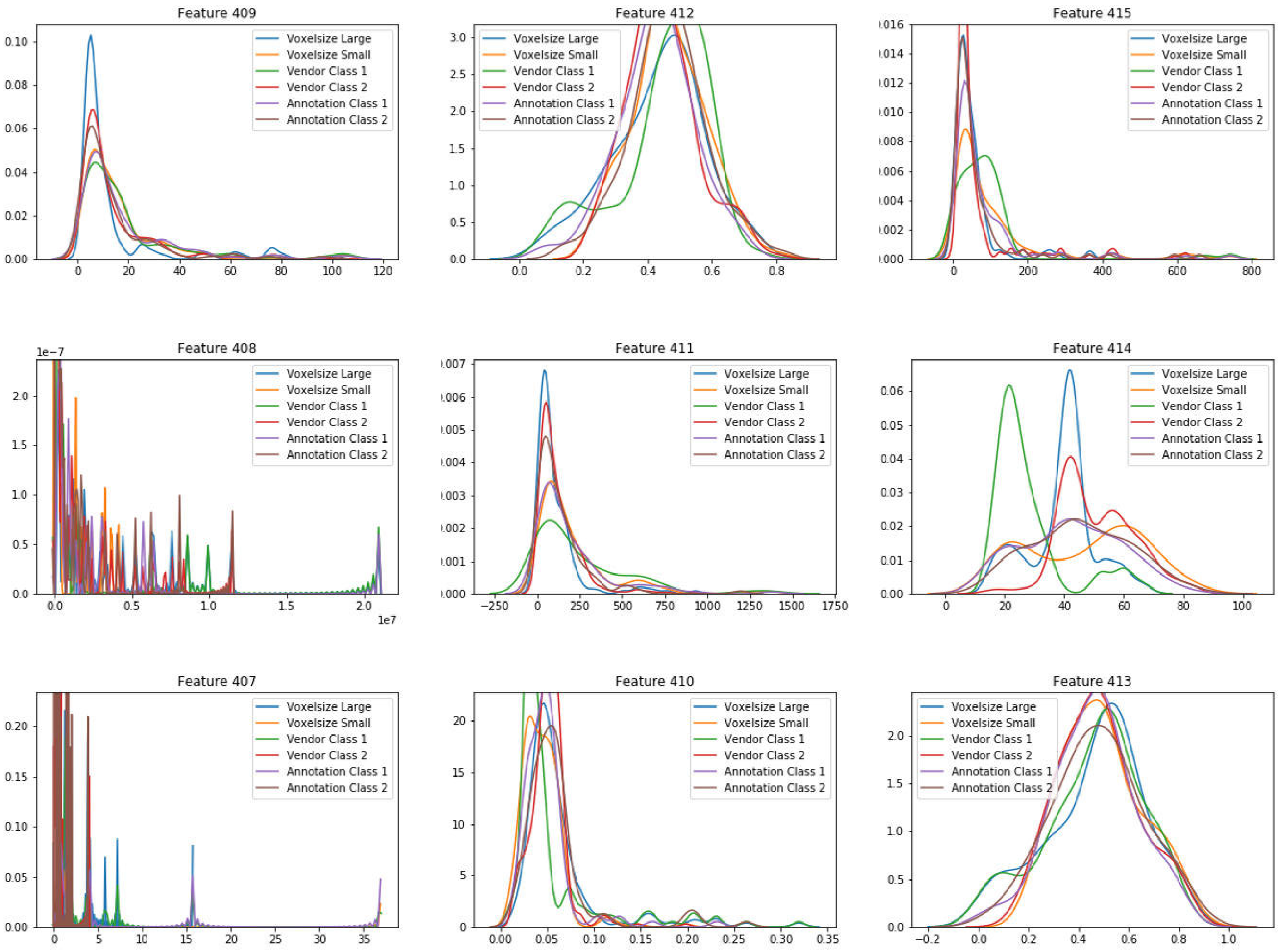
Feature 409
Feature 412
Feature 415
Feature 408
Feature 411
Feature 414
Feature 407
Feature 410
Feature 413
Voxelsize Large
Voxelsize Small
Vendor Class 1
Vendor Class 2
Annotation Class 1
Annotation Class 2

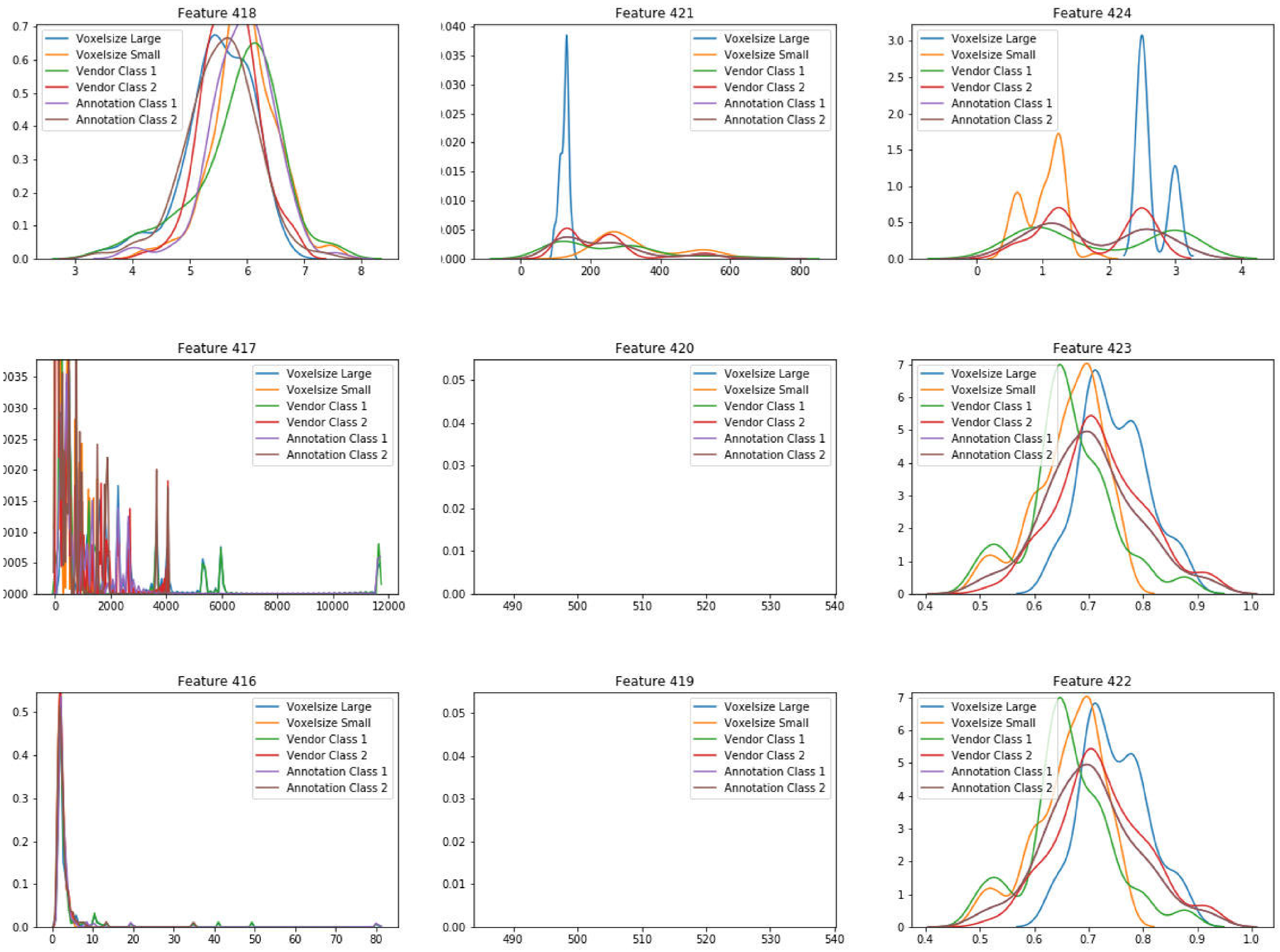
Feature 418
Feature 421
Feature 424
Feature 417
Feature 420
Feature 423
Feature 416
Feature 419
Feature 422
Voxelsize Large
Voxelsize Small
Vendor Class 1
Vendor Class 2
Annotation Class 1
Annotation Class 2

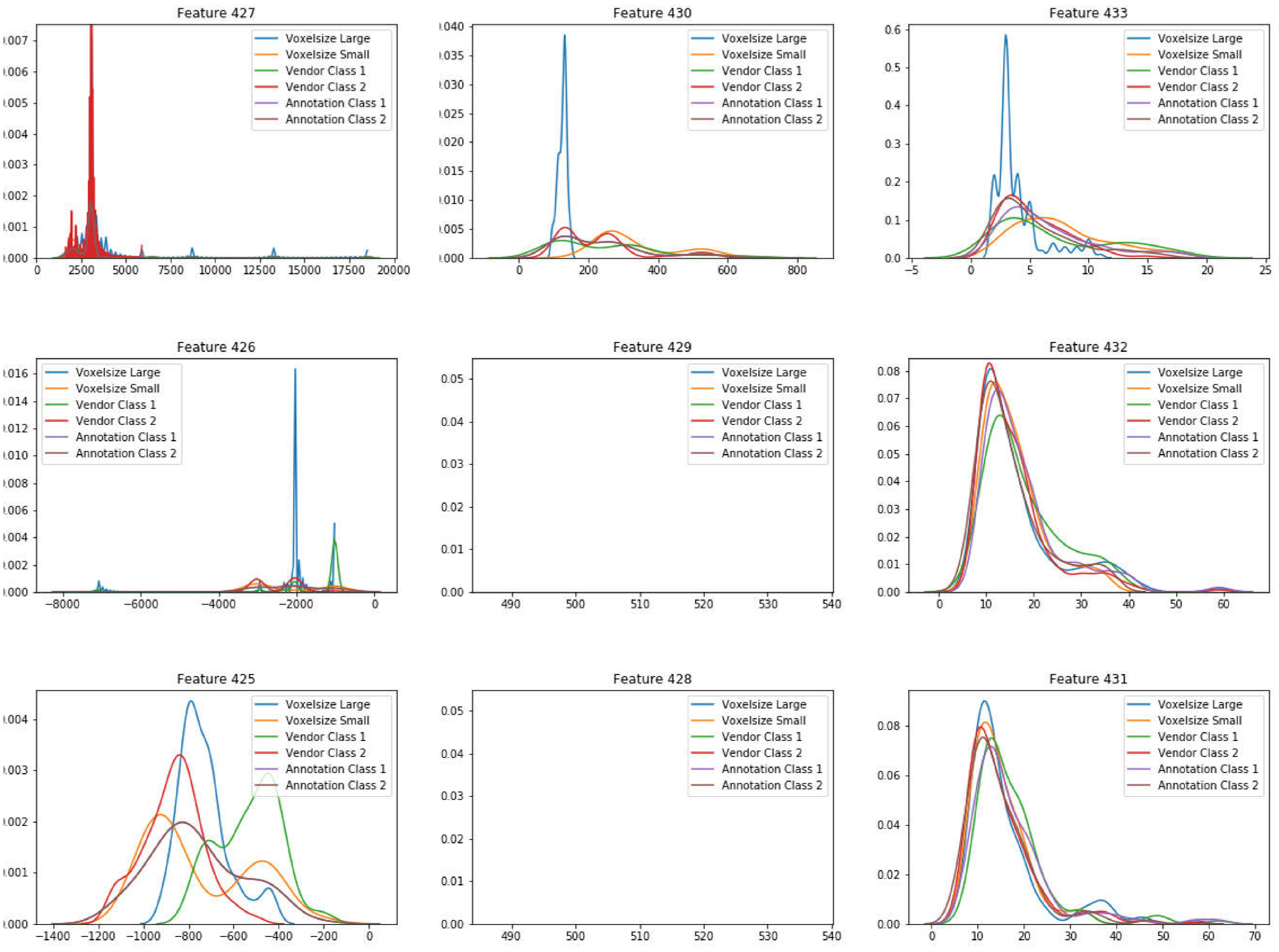
Feature 427
Feature 430
Feature 433
Feature 426
Feature 429
Feature 432
Feature 425
Feature 428
Feature 431
Voxelsize Large
Voxelsize Small
Vendor Class 1
Vendor Class 2
Annotation Class 1
Annotation Class 2

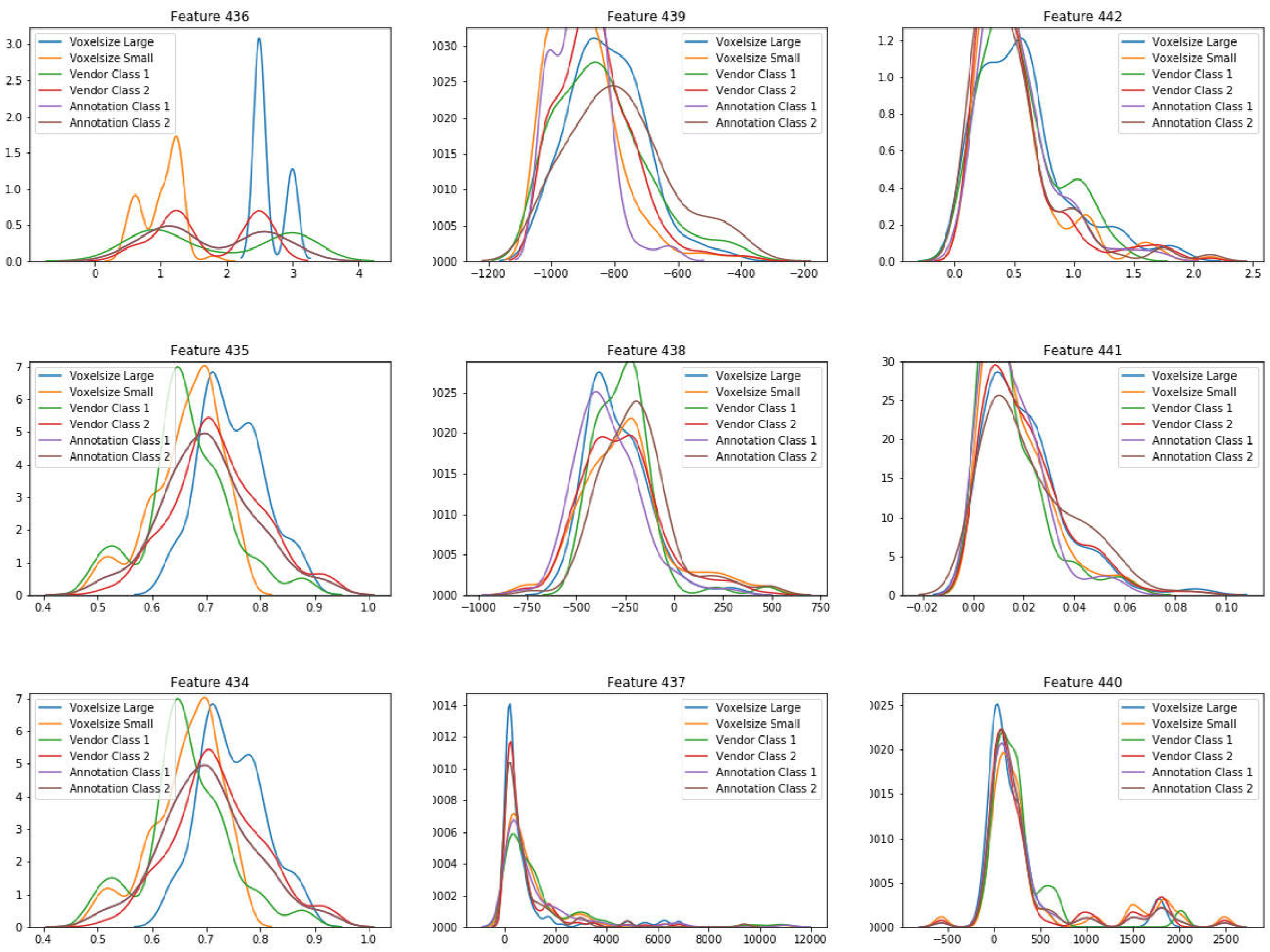
Feature 436
Feature 439
Feature 442
Feature 435
Feature 438
Feature 441
Feature 434
Feature 437
Feature 440
Voxelsize Large
Voxelsize Small
Vendor Class 1
Vendor Class 2
Annotation Class 1
Annotation Class 2

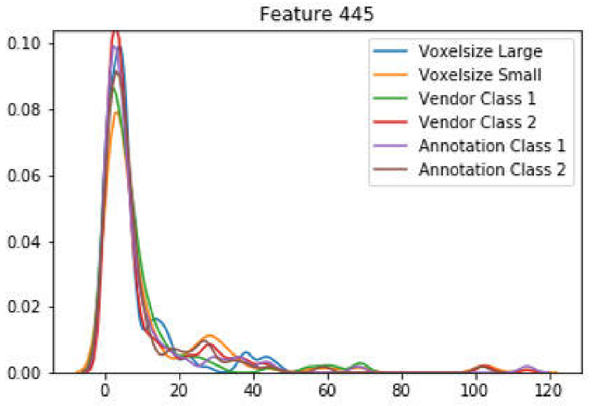
Feature 445
Voxelsize Large
Voxelsize Small
Vendor Class 1
Vendor Class 2
Annotation Class 1
Annotation Class 2

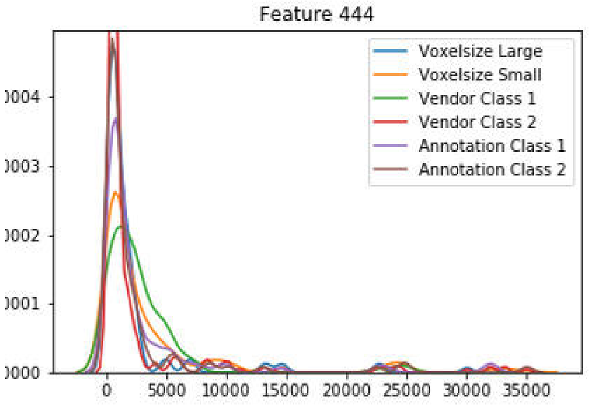
Feature 444
Voxelsize Large
Voxelsize Small
Vendor Class 1
Vendor Class 2
Annotation Class 1
Annotation Class 2

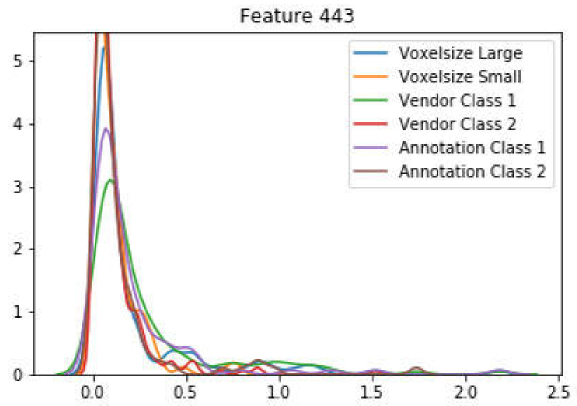
Feature 443
Voxelsize Large
Voxelsize Small
Vendor Class 1
Vendor Class 2
Annotation Class 1
Annotation Class 2
